# Proteome analysis of monocytes implicates altered mitochondrial biology in adults reporting adverse childhood experiences

**DOI:** 10.1038/s41398-023-02320-w

**Published:** 2023-02-01

**Authors:** Johannes C. S. Zang, Caroline May, Birte Hellwig, Dirk Moser, Jan G. Hengstler, Steve Cole, Markus Heinrichs, Jörg Rahnenführer, Katrin Marcus, Robert Kumsta

**Affiliations:** 1 Department of Genetic Psychology, Faculty of Psychology, Ruhr-University Bochum, Bochum, Germany; 2 Medizinisches Proteome-Center, Medical Proteome Analysis Center for Proteindiagnostics (PRODI), Ruhr-Universität Bochum, Bochum, Germany; 3 Department of Statistics, TU Dortmund University, Dortmund, Germany; 4 Leibniz Research Centre for Working Environment and Human Factors at the Technical University of Dortmund (IfADo), Dortmund, Germany; 5 David Geffen School of Medicine, University of California, Los Angeles, CA USA; 6 Department of Psychology, Laboratory for Biological and Personality Psychology, University of Freiburg, Freiburg, Germany; 7 Department of Behavioural and Cognitive Sciences, Laboratory for Stress and Gene-Environment Interplay, University of Luxemburg, Esch-Alzette, Luxemburg

**Keywords:** Psychology, Psychiatric disorders

## Abstract

The experience of adversity in childhood has been associated with poor health outcomes in adulthood. In search of the biological mechanisms underlying these effects, research so far focused on alterations of DNA methylation or shifts in transcriptomic profiles. The level of protein, however, has been largely neglected. We utilized mass spectrometry to investigate the proteome of CD14^+^ monocytes in healthy adults reporting childhood adversity and a control group before and after psychosocial stress exposure. Particular proteins involved in (i) immune processes, such as neutrophil-related proteins, (ii) protein metabolism, or (iii) proteins related to mitochondrial biology, such as those involved in energy production processes, were upregulated in participants reporting exposure to adversity in childhood. This functional triad was further corroborated by protein interaction- and co-expression analyses, was independent of stress exposure, i.e. observed at both pre- and post-stress time points, and became evident especially in females. In line with the mitochondrial allostatic load model, our findings provide evidence for the long-term effects of childhood adversity on mitochondrial biology.

## Introduction

Exposure to traumatic events in childhood, such as the experience of abuse, neglect, or sexual violence is prevalent [1, 2] and associated with increased child mortality and long-term vulnerability to mental or physical disorders and functional impairment [3, 4]. This raised the question of what biological processes are susceptible to adverse experiences and might be involved in mediating the observed associations. So far, research yielded the following insights.

First, there is compelling evidence linking the experience of adversity to dysregulations of the endocrine stress system [5, 6] and the immune system [7]. These manifest, e.g., in attenuated glucocorticoid responses following stress exposure [8, 9] and persistent low-grade inflammation [10, 11]. Both processes are closely related and mitochondria have been suggested as modulators integrating stress [12] and immune system related [13] signaling on the subcellular level [14–16].

Second, following experimental work in animal models [17] a growing body of research has investigated childhood adversity-related alterations of epigenetic modifications, particularly DNA methylation profiles, both for specific genes [18–21] and across the genome [22, 23]. These studies showed mixed results and although reported effects are overall rather small, they seem to indicate that early adversity potentially influences DNA methylation levels of genes involved in the regulation of stress-related glucocorticoid signaling [23] or immune system-relevant processes [22].

Third, more recently research started to focus on the transcriptome as an intermediate molecular level in the study of trauma and stress consequences. For example, in post-traumatic stress disorder (PTSD) patients, specific mRNA expression profiles were observed in individuals with a history of childhood abuse [24]. Further, a mega-analysis combining blood transcriptomic studies in PTSD patients demonstrated dysregulations of inflammatory and stress-related pathways across several types of traumatic experiences and associated interpersonal trauma with sex-specific expression alterations [25]. The majority of conducted transcriptomic studies relies on whole blood without taking into account cell heterogeneity, however, specific blood cell subtypes seem to be particularly sensitive to variation in the social environment.

Enhanced expression of pro-inflammatory immune response genes in leukocytes has been reported for people experiencing loneliness and chronic isolation [26] and among these immune cells, monocytes were identified as transcriptionally most sensitive to subjective social isolation [27], traumatic stress [28] or chronic stress in caregivers of terminally ill patients [29, 30].

Thus, findings on the molecular level underscore the relevance of immune and stress response system dynamics, and make a strong case for studying the molecular consequences of adverse childhood experience within the context of acute stress processing. Previously, we observed differential gene expression patterns in monocytes following exposure to social stress, with increased activity of pro-inflammatory upstream signaling in a sample of healthy adults reporting childhood adversity [31].

Yet, although research on DNA methylation or transcriptomic profiles yielded valuable insights, it remains elusive to what extend childhood adversity is reflected within a cell’s proteomic landscape. Currently, there is a lack of knowledge concerning the role of proteins within the biological processes potentially mediating the consequences of stressful or traumatic experiences. Given that it is proteins that carry out molecular functions and given the generally variable correspondence of expression states across the cellular cascade from mRNA to protein levels [32–34] it is highly promising to include a proteomics perspective to further unravel the mechanisms involved in the long-term effects of childhood adversity.

Here we provide insight into the proteome of CD14^+^ monocytes isolated before and after exposure to psychosocial stress in a sample of healthy adults with a history of childhood adversity and a matched control group using a label-free mass spectrometry approach. Based on the aforementioned evidence, we expected to observe differences in protein expression related to immune responses. However, given the imperfect correspondence between transcriptome and proteome level, we also aimed to explore proteins differentially expressed between the adversity and control groups.

## Material and methods

60 healthy adults (40 female) between 45 and 60 years of age participated in the study. Half of the participants reported the experience of moderate to severe childhood adversity (*n* = 30) assessed with the Childhood Trauma Questionnaire (CTQ), and further validated with the Early Trauma Inventory (ETI). The control group (*n* = 30) reported no experience of childhood adversity. None of the participants met criteria for mental disorders at the time of assessment or in the previous 12 months. Resilience levels were assessed using the Resilience Scale (RS-25) and groups were matched for gender, age, current socioeconomic status (SES), and SES in childhood (Table S1). Ethical approval for this study was obtained from Ethics Committee of the Albert-Ludwigs-University Freiburg (183/11). See Schwaiger et al. 2016 for findings on gene expression following stress exposure and further study details.

### Stress induction

All participants underwent the Trier Social Stress Test (TSST), a well-validated and standardized laboratory stress protocol [35]. Blood was taken at several points for stress hormonal analysis (relative to stress exposure: −45, −1, 1, 10, 20, 30, 45, and 90 min) and monocyte isolation (−45, 45, 180 min).

### Proteome analysis

Proteome analysis was performed on 118 monocyte samples taken at a baseline measurement 45 min before (t0) and 180 min following (t1) stress exposure utilizing liquid chromatography-tandem mass spectrometry (LC-MS/MS) analysis on a Q-Exactive hybrid quadrupole-Orbitrap mass spectrometer. LC-MS/MS analysis detected ~3500 protein groups. Proteins were included in the analysis when identified by at least two unique peptides with valid values in at least eighty percent of participants in at least one of the compared groups or time points. In graphical representations, the evidence level is color coded with yellow indicating the eighty percent criterion and red indicating proteins identified by a hundred percent of valid values. Proteins not meeting the eighty percent criterion as a minimum requirement are colored in grey. Applying these criteria, we identified 1129 proteins at t0 and 1206 proteins at t1. The intersection of both proteomes (the consensus proteome) contained 1119 proteins.

### Statistical analysis

All analyses were carried out using the R environment [36]. First, we focused on differential protein expression (DEP) and compared i) protein intensities between groups (early adversity vs. control: DEP_a_) before and after stress exposure as well as (ii) stress-related changes in protein intensities across time (DEP_s_) in both groups using the limma package [37]. Further, we utilized co-expression network analysis to detect modules of highly co-expressed proteins and analyzed stress-related module preservation or disruption within the consensus proteome by means of the WGCNA package [38]. The significance level for differential analysis was set to 5%, the cut-off for meaningful fold changes was set to FC = 1.3 based on technical variation (SI) and all reported effects are adjusted for the variable sex. Gene-ontology (GO) enrichment analysis was performed utilizing Enrichr [39] and adjusted Fisher’s exact test. Enrichment of DEP_a_ sets against the monocytes whole proteome universe was tested using the topGO package [40]. To estimate similarities between derived GO-Terms, we used semantic similarity analysis [41]. All DEP_a_ and hub proteins of interest (see below) were further referenced against mitochondrial-related proteins as provided in MitoCarta 3.0 [42].

Following WGCNA, we compared module expression differences between sex and condition using linear models based on calculated eigenvalues (eigenproteins) of each module (MEs) that were further correlated with clinical variables. Module preservation analyses across stress induction were carried out using a permutation-based preservation statistic with 500 random permutations of the data [43]. Modules were tested for protein enrichments using the in-package ImmunePathwayLists, brainLists, BloodAtlases, and CHDI lists for enrichment comparison. Resulting *p* values were corrected for multiple testing through the Bonferroni method. For each module, we defined hub proteins based on their intramodular kME (the correlation between each protein and each modules eigenprotein) and we fitted multiple regression models using the R package variancePartition [44] to characterize the percent of module and protein variance explained by variables of interest; including participants CTQ total and RS25 score, age, sex, or BMI. UniProt accessions were supplied to the STRING database [45] to investigate protein-protein interaction networks. We provide a more detailed description of the methods in the supplements.

## Results

### Childhood adversity-related differential protein expression

**Fig. 1:**
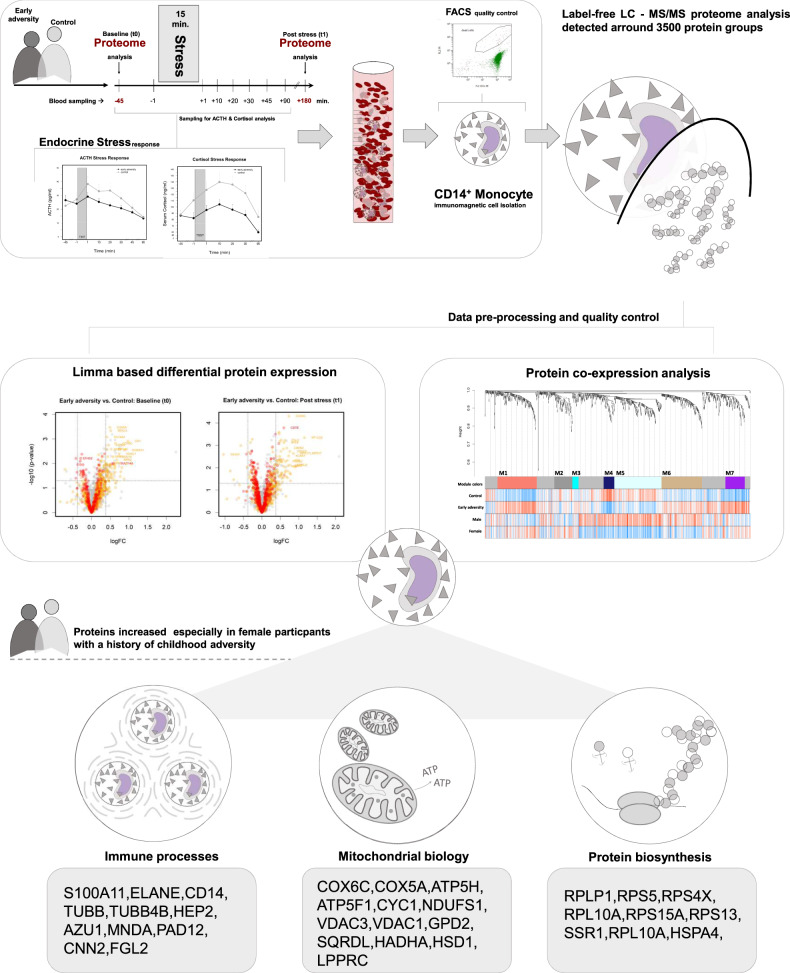
Study design and central results. Healthy adults with a history of childhood adversity and a matched control group were exposed to a 15 min. standardized laboratory stress protocol (Trier Social Stress Test: TSST). Levels of the stress hormones cortisol and ACTH were determined from plasma taken before and after stress exposure (see SI for details). CD14^+^ monocytes were isolated from blood samples 45 min before (t0 baseline measurement) and 180 min following stress (t1 post-stress measurement). Unlabeled LC-MS/MS analysis, followed by differential expression analysis and protein co-expression analysis, was performed to assess differences in protein expression before and after stress, and between groups. We observed higher abundances of proteins involved in the immune system-relevant processes, protein metabolic processes, and mitochondrial biology, especially in females with a history of childhood adversity.

We utilized mass spectrometry to investigate the proteome of CD14^+^ monocytes in healthy adults reporting childhood adversity and a control group before and after psychosocial stress exposure (See Fig. 1 for study design and central results).

Participants of the early adversity group often experienced three or more types of abuse and neglect in childhood and had significantly higher CTQ total scores compared to control individuals. We found no group differences in participant’s age, body mass index, waist-to-hip ratio, smoking, or baseline count of blood cells (Table S1). To investigate the influence of stress on the proteome, we compared protein abundance changes across time (t0 to t1), which showed little detectable effects of stress on the protein level across both groups (DEP_s_ see SI 11 and tables S4–S6 for details on stress effects).

**Fig. 2:**
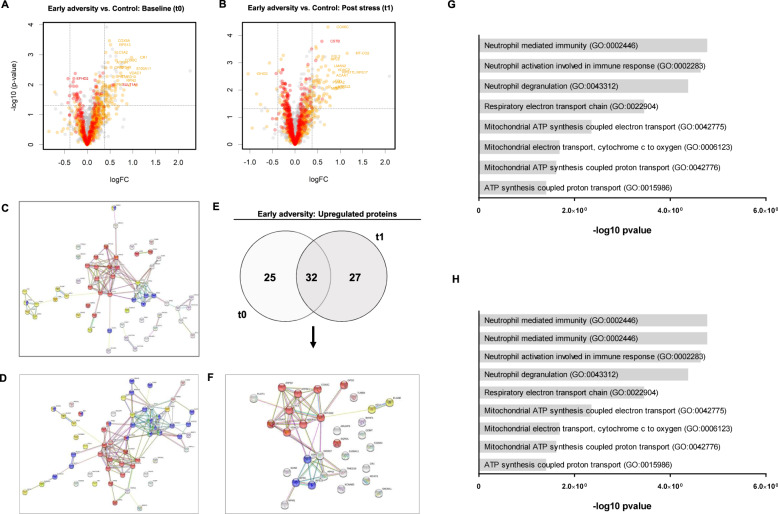
Differential protein expression between groups before and after stress exposure. Volcano plots compare the extent of log_2_ fold-changes (adversity vs. control) and -log_10_ *p* value for protein levels at the t0 baseline measurement (**A**: *n*_prot_ = 1129) and at the t1 post-stress measurement (**B**: *n*_prot_ = 1206). Dashed lines show the |1.3| FC cutoff criterion (vertical) and the 0.05 significance level (horizontal). Colors in volcano plots reflect the 80% (yellow) or 100% (red) validity threshold. Proteins colored in grey did not meet the 80% validity threshold and represent all other proteins. Please note that *p* values did not survive correction for multiple testing. Protein interaction network structures for proteins upregulated in the early adversity group at t0 (**C**) and t1 (**D**) are depicted. Of all upregulated proteins in the early adversity group, 32 were differentially expressed at t0 and t1 (**E**) and formed a consensus network structure (**F**). Proteins colored in red are associated with mitochondrial biology, and proteins in blue are related to the regulation of protein synthesis and transport. Yellow proteins relate to immune processes and neutrophil degranulation. Green proteins in the t1 network are implicated in viral transcription. Barplots represent significantly enriched GO-Terms (Top 10 selection for DEP_a_ based on adjusted p-values) for proteins upregulated in the early adversity at t0 (**G**) and t1 (**H**). For network illustrations in higher resolution, see SI.

In between group analyses, however, limma models revealed significant differences between participants with a history of childhood adversity and control participants in 61 proteins at t0 and 65 proteins at t1, respectively (Fig. 2A, B; Tables S2 and S3). All adversity-related differentially expressed proteins (DEP_a_) had fold changes|≥1.3|. We found 34 DEP_a_ to be differentially expressed at t0 and t1, leading to a total of 92 unique DEP_a_ overall. 92% of all DEP_a_ were upregulated in the early adversity group and a common set of 32 proteins was upregulated in the early adversity group at t0 and t1. (Fig. 2E, Table 1).

**Table 1:** Proteins differentially expressed (up - and downregulated) in participants with a history of early adversity before and after stress exposure.

UniProt ID	Gene	Protein	Gene ontolotgy		Pre-stress (t0)		Post-stress (t1)	
			Biological process	Cellular component	FC	*p*	FC	*p*
P08246	ELANE	Neutrophil elastase	Acute inflammatory response to antigenic stimulus [GO:0002438	Azurophil granule lumen [GO:0035578]	2.1	0.010	1.7	0.019
P17927	CR1	Complement receptor type 1	Complement activation, classical pathway [GO:0006958];	Cell surface [GO:0009986]	2.0	0.001	1.6	0.005
P00403	MT-CO2	Cytochrome c oxidase subunit 2	ATP synthesis coupled electron transport [GO:0042773	Integral component of membrane [GO:0016021]	1.9	0.010	2.2	<0.001
P31949	S100A11	Protein S100-A11	Negative regulation of DNA replication [GO:0008156]	Cell-cell adherens junction [GO:0005913]	1.9	0.003	1.5	0.042
P20160	AZU1	Azurocidin	Antimicrobial humoral response [GO:0019730]	Azurophil granule [GO:0042582]	1.8	0.043	1.6	0.028
P46782	RPS5	40 S ribosomal protein S5	SRP-dependent cotranslational protein targeting to the membrane [GO:0006614]	Cytosol [GO:0005829]	1.7	0.015	1.6	0.008
Q9UH99	SUN2	SUN domain-containing protein 2	Centrosome localization [GO:0051642]	Condensed nuclear chromosome [GO:0000794]	1.7	0.022	1.7	0.038
Q3ZCM7	TUBB8	Tubulin beta-8 chain	Microtubule cytoskeleton organization [GO:0000226]	Cytoplasm [GO:0005737]	1.7	0.034	1.9	0.009
P04844	RPN2	Dolichyl-diphosphooligosaccharide-protein glycosyltransferase subunit 2	Aging [GO:0007568]	Autophagosome membrane [GO:0000421]	1.6	0.007	1.5	0.027
P09669	COX6 C	Cytochrome c oxidase subunit 6C	Generation of precursor metabolites and energy [GO:0006091]	Integral component of membrane [GO:0016021]	1.6	0.002	1.7	<0.001
O75531	BANF1	Barrier-to-autointegration factor;	DNA integration [GO:0015074]	Condensed chromosome [GO:0000793]	1.5	0.012	1.4	0.018
P06748	NPM1	Nucleophosmin	CENP-A containing nucleosome assembly [GO:0034080]	Centrosome [GO:0005813]	1.5	0.014	1.5	0.009
P45880	VDAC2	Voltage-dependent anion-selective channel protein 2	Anion transport [GO:0006820]	Acrosomal vesicle [GO:0001669]	1.5	0.016	1.7	0.002
Q9Y6N5	SQRDL	Sulfide:quinone oxidoreductase, mitochondrial	Hydrogen sulfide metabolic process [GO:0070813]	Mitochondrial inner membrane [GO:0005743]	1.5	0.010	1.4	0.044
P49755	TMED10	Transmembrane emp24 domain-containing protein 10	COPI coating of Golgi vesicle [GO:0048205]	COPI-coated vesicle [GO:0030137]	1.5	0.005	1.4	0.005
Q13303	KCNAB2	Voltage-gated potassium channel subunit beta-2	NADPH oxidation [GO:0070995]	axon terminus [GO:0043679]	1.5	0.042	1.5	0.017
P61586	RHOA; RHOC	Transforming protein RhoA	G protein-coupled receptor signaling pathway [GO:0007186]	Apical junction complex [GO:0043296]	1.5	0.016	1.4	0.003
O75915	ARL6IP5	PRA1 family protein 3	L-glutamate transmembrane transport [GO:0015813]	cytoskeleton [GO:0005856]	1.4	0.028	1.4	0.021
P62277	RPS13	40 S ribosomal protein S13	SRP-dependent cotranslational protein targeting to membrane [GO:0006614]	Cytosol [GO:0005829]	1.4	0.001	1.3	0.005
P39656	DDOST	Dolichyl-diphosphooligosaccharide-protein glycosyltransferase 48 kDa subunit	T cell activation [GO:0042110]	Azurophil granule membrane [GO:0035577]	1.4	0.011	1.5	0.004
O14880	MGST3	Microsomal glutathione S-transferase 3	Glutathione derivative biosynthetic process [GO:1901687]	Endoplasmic reticulum [GO:0005783]	1.4	0.011	1.4	0.022
P28331	NDUFS1	NADH-ubiquinone oxidoreductase 75 kDa subunit, mitochondrial	ATP metabolic process [GO:0046034]	Mitochondrial intermembrane space [GO:0005758]	1.4	0.013	1.4	0.005
P21964	COMT	Catechol O-methyltransferase	Catecholamine catabolic process [GO:0042424]	Axon [GO:0030424]	1.4	0.050	1.5	0.006
O75947	ATP5H	ATP synthase subunit d, mitochondrial	ATP biosynthetic process [GO:0006754]	Mitochondrial inner membrane [GO:0005743]	1.4	0.002	1.4	0.001
P43307	SSR1	Translocon-associated protein subunit alpha	IRE1-mediated unfolded protein response [GO:0036498]	Endoplasmic reticulum [GO:0005783]	1.4	0.031	1.3	0.012
Q00325	SLC25A3	Phosphate carrier protein, mitochondrial	Phosphate ion transmembrane transport [GO:0035435]	Extracellular exosome [GO:0070062]	1.3	0.039	1.3	0.016
O75955	FLOT1	Flotillin-1	Axonogenesis [GO:0007409]	GABA-ergic synapse [GO:0098982]	1.3	0.027	1.3	0.006
Q9NTJ5	SACM1L	Phosphatidylinositide phosphatase SAC1	Phosphatidylinositol biosynthetic process [GO:0006661]	AMPA glutamate receptor complex [GO:0032281]	1.3	0.017	1.3	0.047
P08574	CYC1	Cytochrome c1, heme protein, mitochondrial	Mitochondrial ATP synthesis coupled proton transport [GO:0042776]	Integral component of membrane [GO:0016021]	1.3	0.021	1.4	0.001
P26447	S100A4	Protein S100-A4	Epithelial to mesenchymal transition [GO:0001837]	Collagen-containing extracellular matrix [GO:0062023]	1.3	0.018	1.3	0.008
P24539	ATP5F1	ATP synthase F(0) complex subunit B1, mitochondrial	ATP biosynthetic process [GO:0006754]	Membrane [GO:0016020]	1.3	0.031	1.4	0.002
P43304	GPD2	Glycerol-3-phosphate dehydrogenase, mitochondrial	Camera-type eye development [GO:0043010]	Glycerol-3-phosphate dehydrogenase complex [GO:0009331]	1.3	0.003	1.3	0.008
Q96C19	EFHD2	EF-hand domain-containing protein D2	-	Membrane raft [GO:0045121]	0.7	0.006	0.7	0.002
P01876	IGHA1	Ig alpha-1 chain C region	B-cell receptor signaling pathway [GO:0050853]	Blood microparticle [GO:0072562]	0.6	0.018	0.6	0.014

*FC* observed fold changes in proteins expression. *p* limma derived significance level. Evidence level indicating the percentage of proteins identified by valid value in at least one of the compared groups was ≥ 0.8 for all proteins.

Protein interaction networks generated from DEPa identified at t0 and t1 and their intersection indicate a conserved network structure of highly connected proteins that play a role in mitochondrial biology and protein synthesis (Fig. 2C–F).

Proteins related to mitochondrial biology include for example subunits of the cytochrome C oxidase (COX6), the terminal enzyme complex of the respiratory chain (complex IV) as well as subunits of the mitochondrial ATP synthase (ATP5H), the enzyme catalyzing the synthesis of ATP as universal intracellular energy carrier through phosphorylation. Of all 92 DEPa, 26 (28%) are referenced in MitoCarta 3.0 as mitochondrial (Table S7) and functionally linked to, e.g., the oxidative phosphorylation pathway (*n*_prot_= 9) or mitochondrial metabolism (*n*_prot_= 11). Among the proteins that associate with a role in protein-related processes, two are part of the 40 s ribosomal subunit (RPS13, RPS5) and function in protein synthesis, while DDOST, SSR1, and RPN2 play part in protein translocation. These results were stable when using different imputation methods applied to the data to replace missing values (Figure S3).

GO-terms associated with upregulated DEP_a_ identified at t0 (*n* = 57) and t1 (*n* = 59) are depicted in Fig. 2G, H.

Exploring pairwise semantic similarities between all GO-terms associated with t0 and t1 DEP_a_, we built ten cluster solutions of biological processes (Figure S4) upregulated in the early adversity group. Upregulated processes indicate that childhood adversity was associated with altered cell-type specific functions such as monocyte activation (GO: 0042117) and antimicrobial processes (GO: 0002777), accompanied by elevated signaling (GO: 0002777) and regulation of the cell’s motility (GO: 0002777), as well as with altered housekeeping processes such as homeostasis (GO: 0033762) and energy metabolism (GO: 0042773). Results further indicated that these effects were independent of stress exposure, i.e., likely reflect stable differences.

### Sex-specific effects

**Fig. 3:**
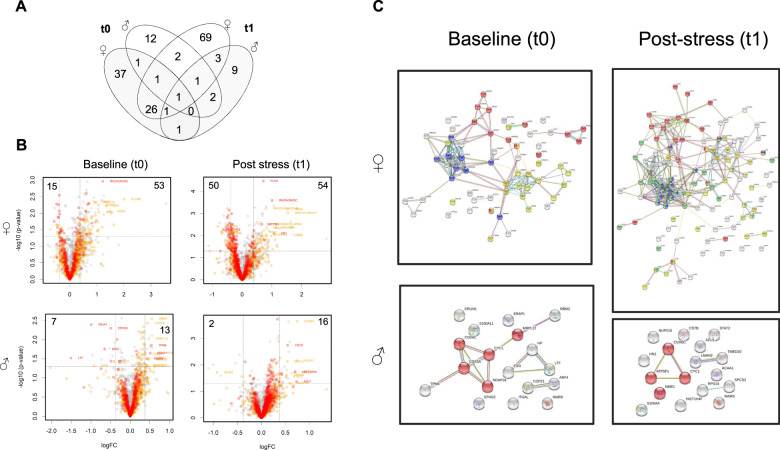
Sex specific protein expression differences before and after stress exposure. Across all groups compared, the largest overlap in differentially expressed proteins became evident in female participants (**A**). Depicting the underlying analysis, volcano plots show the extent of log_2_ fold-changes (adversity vs. control) and -log_10_ *p* value for protein levels at the t0 baseline measurement (*n*_prot_ = 1129) and at the t1 post-stress measurement (*n*_prot_ = 1206) for female (*n* = 40) and male (*n* = 19) participants. Please note that the imbalanced sex distribution might account to some extent for these effects. Dashed lines show the |1.3| FC cutoff criteria (vertical) and the 0.05 significance level (horizontal). Significantly up- (right quarter) or downregulated (left quarter) proteins are indicated by numbers. Please note that *p* values did not survive correction for multiple testing. Colors in volcano plots reflect the 80% (yellow) or 100% (red) validity threshold. Proteins colored in grey did not meet the 80% validity threshold and represent all other proteins (**B**). String networks were generated from proteins differentially expressed in male and female participants with a history of childhood adversity at t0 and t1. Colors indicate the biological function or cellular location. Blue proteins function in protein biosynthesis, red proteins map to mitochondria as a cellular component, yellow proteins play a role in the immune system relevant processes, and green proteins are associated with protein localization processes (**C**).

The imbalanced sex distribution in our sample and the smaller number of investigated males (*n* = 19 vs *n* = 40 females), likely account to some extend for sex-specific effects we found. Yet, since sex-specific effects of childhood adversity at the molecular level have already been reported, we did not want to withhold our observations, and thus display them with due caution. As such, comparing adversity-related expression differences separately for sex, we observed 58 DEP_a_ in female compared to 20 DEP_a_ in male participants. Following stress exposure, number of DEP_a_ was higher in female (DEP_a_ = 104), but lower in male participants (DEP_a_ = 18). Larger number of DEP_a_ in females was mainly driven by higher abundance of downregulated proteins (+35 DEP_a_) after stress exposure. The largest overlap of DEP_a_ between pre- and post-stress time points was observed for female participants $({n_{{\rm{DEP}}}}_{\rm{a}} = 29)$ (Fig. 3A, B). String networks generated from DEP_a_ show clear differences in structure, density, and functional annotation between female and male participants. As such, baseline DEP_a_ networks of female participants are comprised of interrelated but distinct clusters of proteins functionally involved in protein synthesis, mitochondrial biology, and immune system processes. Following stress, this tri-parted network is further enriched and complemented by a cluster of proteins that more specifically play a role within the establishment of protein localization. In contrast, networks generated from DEP_a_ of male participants are rather loosely connected and of minor density. The proteins interrelated within this assembly play a role in mitochondrial biology, which suggests that altered mitochondrial protein expression is associated with childhood adversity in both females and males.

### Protein co-expression and stress-related module preservation/disruption

**Fig. 4:**
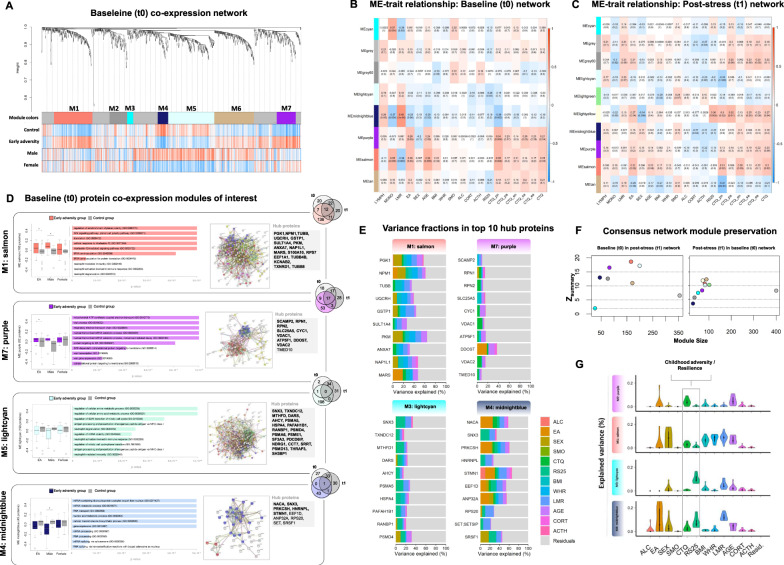
Consensus protein co-expression network analysis. Dendrogram (**A**) of the consensus baseline proteome comprising the 1119 proteins that represent the intersection of proteins identified at the t0 baseline measurement and the t1 post-stress measurement. Each line represents a protein (leaf) and each low-hanging cluster represents a group of proteins with similar network connections (branch) on the tree. The first band underneath the tree indicates the seven detected modules and subsequent bands indicate protein-trait correlation. Red indicates a strong positive relationship and blue indicates a strong negative relationship. Module eigenprotein (ME) correlation with clinical variables are depicted for modules derived from co-expression networks built upon baseline (t0) and post-stress (t1) measurements of the consensus proteome (*n*_prot_ = 1119) (**B**, **C**: LYMPH & MONO = count of lymphocytes and monocytes, LMR = lymphocyte to monocyte ratio, EA = early adversity group, SEX = biological sex, AGE = age in years at participation, BMI = body mass index, WHR = waist to hip ratio, SMO = smoking, ALC = consumption of alcohol, CORT & ACTH = Cortisol and ACTH base to peak ratio, RS25 = Resilience Scale 25 total score, CTQ_pn = CTQ physical neglect score, CTQ_pa = CTQ physical abuse score, CTQ_en = CTQ emotional neglect score, CTQ_sa = CTQ sexual abuse score, CTQ_ea = CTQ emotional abuse score, CTQ = Childhood trauma questionnaire total score). **D** the associations between ME values and early adversity (coloured boxplots) and control group (grey boxplots) are shown stratified by sex (*indicates *p* < 0.05; please note that *p* values did not survive correction for multiple testing). For each module, the 10 most significantly enriched biological processes are reported and ranked by -log10 *p* values). Venn Diagrams indicate the overlap of baseline module proteins and DEP_a_ identified at t0 and t1. String networks were generated for each of the presented modules. Protein colors indicate functional or topological associations within the salmon module (red: mitochondrion, blue: translation, yellow: immune response, pink: viral processes), the purple module (red: ATP metabolic processes, blue: translational initiation, yellow: immune response), the light cyan module (red: vesicle-mediated transport, yellow: immune system processes, blue: viral processes, green: mRNA splicing) and the midnight blue module (red: aerobic respiration, blue: gene expression). High-resolution STRING network illustrations for modules of interest and details about all other modules are provided in the SI. Preservation (*Z*_summary_) of baseline (t0) consensus modules in the post-stress (t1) consensus network and vice versa is depicted by plotting the module-specific Z_summary_ score against the module size (**F**). We report the top 10 and top 10 percent (bold) of the module-associated hub proteins in grey boxes. Bar plots (**E**) represent partitions of variance explained by a set of variables within the top 10 hub proteins of M1, M7, M4, and M3. Violin plots represent the overall distribution of variance explained by each variable within the top ten module-specific hub proteins. Note that residuals are not plotted (**G**).

Protein co-expression networks were constructed separately for t0 and t1 proteins based on an integrated dataset including proteins that met identification criteria at both time points (*n*_prot_ = 1119). The *Z*_summary_ statistic as a permutation-based preservation indicator was used to compare the preservation of identified t0 baseline modules in the t1 stress network and vice versa. Within the t0 network (Fig. 4A), WGCNA identified seven modules harboring altogether 765 proteins. These modules consist of proteins that are aggregated on the basis of similar expression profiles. Such co-expression structures describe biological processes, and their analysis provides a more systemic perspective toward molecular processes.

The majority of these modules were clearly preserved (*Z*_summary_ > 2) in the t1 network and we found the highest preservation scores (*Z*_summary_ > 15) for the salmon, the light cyan, and the purple module (Fig. 4C).

The purple module (M7: 82 proteins) is comprised of proteins implicated in mitochondrial biology and cellular respiration and further contains proteins involved immune responses and viral gene expression or translation (Fig. 4E). The salmon module (M1: 166 proteins) is implicated in immune system processes, enriched for proteins regulating translation through tRNA aminoacylation and involved in the Interleukin 12 and Wnt signaling pathway. Both, the purple and salmon modules were significantly stronger expressed in the early adversity group compared to the control group, and each contained proteins (*n*_DEPa salmon_ = 30, *n*_DEPa purple_ = 29) identified as differentially expressed between the groups using limma. Consequently, the module *eigenprotein* (as analog to *eigengene*) of both the salmon (*r* = 0.28, *p* < .05) and the purple module (*r* = 0.28, *p* < 0.05) were positively correlated with experience of early adversity (Fig. 4B). While the purple module showed a significant correlation with the CTQ total score (*r* = 0.27, *p* < 05), the salmon (*r* = 0.35, *p* < 05) and the purple module (*r* = 0.34, *p* < 05) correlated positively with participants' score on the CTQ physical neglect subdimension.

Both, the light cyan module (199 proteins) and the midnight blue module (45 proteins) were more strongly expressed in control individuals and particular male control participants showed significantly stronger expression of the midnight blue module. The light cyan module, among others, harbors a set of proteasome proteins implicated in the mediation of immune system processes such as antigen presentation and proteins involved in regulation of mRNA stability. The string network generated from this module showed a dense structure including proteins involved in vesicle-mediated transport and a cluster of proteins functioning in the context of RNA splicing (Fig. 4D). The midnight blue module includes two DEP_a_ is strongly conserved (*Z*_summay_ > 10) in the stress network, and mainly involved in gene expression and the regulation of RNA metabolic processes but as well contains a few proteins involved in cellular respiration. The module’s eigenprotein value was negatively associated with the experience of adversity and correlated negatively with the CTQ physical neglect subdimension (*r* = −0.39, *p* < .05).

We observed no significant expression differences between groups within the other baseline modules (see Fig. 4 for associated biological functions, protein-interaction networks, and hub proteins). The post-stress consensus network (Figure S16) comprised nine modules, which were less preserved (Mean *Z*_summay_ = 8.4) in the baseline network than the other way around (Mean *Z*_summay_ = 11.2) (Fig. 4B). In four of these post stress modules, significant expression differences between male participants with a history of childhood adversity and control individuals became evident (see Figure S16).

Next, we analyzed to what extent proteins of conserved baseline and post-stress co-expression networks overlapped with the DEP_a_ identified using limma analysis and paid particular attention to hub proteins. Hub proteins are part of highly correlated protein assemblies in modules and are likely important from a co-expression perspective. We focused our analysis on the four most conserved modules indicating differential activities between groups and report both, top ten and top ten percent of proteins with the highest kME values for each module in Fig. 4.

Three of the DEP_a_ identified before and after stress exposure were part of the midnight blue or the light cyan module and none of these proteins represented a hub protein. The salmon module on the other hand comprised altogether 30 DEP_a_. Among these, S100A11, a calcium-binding protein, is mechanistically involved in toll-like receptor-mediated inflammatory responses of macrophages [46]. Interestingly, 41% of the salmon modules hub proteins were differentially expressed between groups (TUBB, SULT1A4, TXNRD1, NAP1L1, RPS7, NPM1, and KCNAB2). The purple module includes altogether 29 DEP_a_ of which eight were hub proteins (VDAC1, RPN1, RPN2, CYC1, ATP5F1, VDAC2, TMED10, and DDOST). Note that half of these proteins locate in the mitochondrial membrane and all are highly connected in protein-protein interaction networks (see Fig. 4 and Table S8).

For the four modules of interest, we further fitted multiple regressions towards the module’s eigenproteins and the identified hub proteins to assess the individual influence of a set of variables including, e.g., CTQ & RS25 scores, age, sex, BMI, on each module and hub protein while simultaneously correcting for the other variables using the package variancePartition^35^. Variance fractions for the top ten module-specific hub proteins and the distribution of variance explained by each clinical variable within the respective protein sets are depicted in Fig. 4F.

On the level of module expression, analyses revealed participants’ CTQ total scores and RS25 scores as partly opposing sources of variance. As such, participant’s CTQ but not RS25 scores explained variance in the purple (3.95%) and the salmon (2.91%) modules expression. Vice versa, participants’ RS25 score explained 10.09% of variance of the light cyan modules expression, while the variance accounted for by the CTQ score was negligible. Both, CTQ (3.17%) and RS25 scores (7.85%) explained variance in the midnight blue module´s eigenprotein, as did biological sex (7.44%) and the experience of childhood adversity (18.63%) after controlling for all other variables. These relations mirror the results of our previous analyses and are as well reflected within the expression of the top 10 module hub proteins (Fig. 4F). Among these, the CTQ total score accounted for variation within VDAC1 (9.32%), NACA (8.99%), VDAC2 (7.34%), ANXA7 (6.22%), NAP1L1 (6.21%), RPN2 (5.64%), MARS (5.52%), while RS25 scores particularly accounted for substantial proportions of variation in expression of SNX3 (16.76%), SRSF1 (15.06%), MTHFD1 (14.22%), PSMAS (13.59%) and EEF1D (10.32%).

To summarize, utilizing WGCNA to calculate baseline (t0) and post-stress (t1) consensus protein-co-expression networks, we identified several modules representing specific aspects of monocytes cellular biology. These modules harbor, e.g., proteins relevant for immune response mechanisms or implicated in mitochondrial biology. Comparing protein co-expression with differential protein expression analyses, consistent results emerged. Proteins previously identified as differentially expressed between the early adversity and control condition were mostly co-expressed, predominantly assigned to distinct modules, often represent hub proteins, and are thus likely to play an important function within the co-expression network.

## Discussion

Exposure to adversity in childhood not only causes immediate harm but is also associated with a lifelong increased risk for a range of health problems, raising the question of how early psychosocial experiences are *biologically embedded*. Our proteome analysis of CD14^+^ monocytes revealed three major findings: First, we observed differentially expressed proteins in participants with a history of childhood adversity, which mapped to specific functional domains: mitochondrial biology, immune system processes, and protein synthesis. Second, we observed indications for sex-specific expression differences between groups suggesting stronger effects of adversity on protein expression within female participants while differentially expressed proteins in males were restricted to mitochondrial biology. Third, stress exposure was not associated with major changes in protein expression. The fact that we observed clear effects of stress on the RNA level in our previous work [31] and given the different biological domains which appeared as adversity related when investigating the RNA and protein level (we did not find indications for altered mitochondrial biology on the RNA level) both imply that the effects of adversity are not necessarily represented across molecular levels in the same way. The absence of a clear stress effect on the proteome level may reflect the longer duration required for an acute event to affect protein levels relative to (preceding) RNA levels. As such, the present proteomic differences should be interpreted as predominately reflecting stable differences in monocyte function.

Specifically, GO analysis of upregulated DEP_a_ revealed adversity-associated proteins to function within three particular domains of the monocytes biology: (i) immune relevant processes (e.g., ELANE, AZU1, S100A11), (ii) protein metabolic processes (e.g., RPS13, RPS5, SSR1) and (iii) mitochondrial biology and the cell’s energy metabolism (e.g., MT-CO2, COX5A, COX6C, CYC1, VDAC2, ATP5H or ATP5F1).

Of note, DEP_a_ identified at t0 and t1 were strongly enriched for GO terms indicating neutrophil degranulation and activation. This study analyzed isolated monocytes, so it is unlikely that these GO annotations reflect alterations in neutrophil biology per se. However, monocytes and neutrophils share a common developmental pathway as myeloid lineage immune cells, and both include an extensive system of granule proteins involved in cellular activation, chemotaxis, adhesion, reactive oxygen responses, and inflammatory biology. Hence, we believe that the identification of proteins previously associated with neutrophil activity in our isolated monocyte samples is indicative of the broader interplay of myeloid lineage innate immune cells involved in the orchestration of acute immune responses and inflammatory activity.

Further, protein interaction networks generated from t0 and t1 DEP_a_ showed distinct but interrelated clusters of interconnected proteins related to cell respiratory energy metabolism or to protein biosynthesis. These protein-interaction network structures were conserved across stress exposure and built around a set of 32 upregulated consensus DEP_a_. Importantly, proteins in this set are derived from two repeated measurements and can thus be considered technical replicates.

The number of DEP_a_ in female participants exceeded the numbers found in male participants. Furthermore, proteins found to be differentially expressed between females with a history of childhood adversity and control group participants form clearly structured interaction networks mirroring the functional triad described above. In contrast, proteins differentially regulated between groups of male participants form only loosely connected network structures including proteins involved in mitochondrial biology. The smaller number of investigated males (*n* = 19 vs *n* = 40 females), and thus lower statistical power, might explain these disparities. On the other hand, sex-specific regulation of immune-relevant processes was documented before on basis of a mega-analysis across several transcriptomic studies [25], supporting the potential for female-specific alterations on the molecular level, especially in the context of interpersonal trauma experiences.

Baseline protein co-expression analysis validated findings from the differential protein expression approach and revealed modules representing distinct aspect of monocytes function related to childhood adversity and resilience.

Childhood adversity-associated expression differences became evident within four modules. A salmon module (M1:166 proteins) was implicated in the regulation of immune system responses and harbors proteins involved within IL-12 signaling. This pathway promotes inflammation and facilitates innate as well as adaptive immune responses, modulating differentiation of T helper cells and NK cell toxicity. The salmon module was significantly stronger expressed in participants with a history of childhood adversity and correlated positively with the CTQ total score. S100A11, a member S100 protein family, is one of the proteins of this module. S100A11 has been reported to stimulate the production of the pro-inflammatory cytokine IL-6 by peripheral blood mononuclear cells (PBMC) and overexpression of S100A11 was associated with inflammation-related health problems such as osteoarthritis or rheumatoid arthritis [47].

Both a light cyan (ME 5: 199 proteins) and a midnight blue (ME 4: 45 proteins) module were more strongly expressed in male control participants. While the first module seems implicated in immune-relevant processes and harbors proteins involved in, e.g., vesicle-mediated transport, both modules contain proteins that play a role in gene regulation, particularly influencing RNA metabolic processes.

Notably, resilience scores explained substantial proportions in variation in the light cyan and the midnight blue modules top 10 hub proteins, while CTQ scores accounted for additional variance in the midnight blue modules top 10 hub proteins. Hence, both modules might represent promising targets for future studies regarding risk and resilience mediating processes and sex-specific responses to adversity on the molecular level.

Lastly, the purple module (M7: 82 proteins) harbors altogether 29 DEP_a_ (35% percent of the sizes of the modules), is significantly enriched with mitochondrial proteins, significantly stronger expressed in the early adversity group, and positively correlated with the CTQ total score. This is in line with a number of studies implicating alterations in mitochondrial biology associated with the experience of early adversity [22]. For example, Tyrka and colleagues [48] found higher mtDNA copy numbers in participants with childhood adversity and Boeck and colleagues [49] reported positive correlations between CTQ total scores and ATP-production-related respiration in PBMCs from women with a history of childhood adversity. Especially the mitochondrial allostatic load model emphasizes the central role of mitochondria in every cell’s energy metabolism and focuses on stress-related mitochondrial dysfunction that results, e.g., from elevated glucocorticoid levels and affects cellular and consequently organ or system functioning. Our findings provide evidence on the protein level supporting the mitochondrial allostatic load model [12, 14, 15] with results derived from CD14^+^ monocytes specifically.

Monocytes are antigens representing immune cells and progenitors of macrophages implicated in the first line of defense as part of the innate immune response and play a critical role in immune-to-brain communication processes [50, 51]. Further, monocytes have been identified as the cell type most responsive to socio-environmental conditions [27], and transcriptional change in monocytes was observed following traumatic [28] or chronic stress in caregivers of terminally ill patients [29, 30]. Altogether, there is reason to believe that functional shifts in CD14^+^ monocytes play a role in mediating the systemic effects of early adversity. On the subcellular level, mitochondrial recalibrations might indicate higher cellular energy demands due to such functional shifts.

Since childhood adversity is strongly linked to persistent low-grade inflammation, a greater amount of mitochondrial respiratory chain proteins in CD14^+^ monocytes could as such reflect rather stable adaptions towards the increase in physiological energy costs resulting from higher pro-inflammatory activities. As we observed an increase in proteins involved in mitochondrial biology in participants with a history of childhood adversity independently from acute psychosocial stress exposure, our results point in this direction. This is consistent with findings from Gumpp et al. [52], showing that exposure to childhood maltreatment was linked to higher mitochondrial respiration and density in females. Given that mitochondria are passed from mother to child, developmental programming of mitochondrial biology might also provide a model explaining the intergenerational effects of exposure to adversity. Yet, in contrast to direct effects on the mother, the maternal experience of childhood adversity showed no associations with newborn mitochondrial characteristics. Gyllenhammer and colleagues [53], however, reported higher levels of mitochondrial respiratory chain enzymatic activities and higher mitochondrial content in the offspring of mothers experiencing higher biological stress. Together this underlines the idea of mitochondria as cellular components particularly involved in developmental mechanisms programming biological systems towards the demands of stressful environments.

The following limitations need to be considered. The sample size was small. Nevertheless, 118 mass spectrometry analyses could be realized, which is relatively large and therefore quantitatively robust by the standards of global proteomics studies. Further limitations arise from the chosen methodology and the preparation strategies used. Here, we applied a rather generalist protocol for protein extraction, designed to collect a representative part of the monocytes protein assembly instead of targeting specific proteins bound to membranes or presented in distinct cell organelles. Consequently, the proteins identified represent a fraction of the monocytes proteome and give valuable insights but inevitably do not display the complete picture. Lastly, it is important to keep in mind that the significant findings presented did not survive correction for multiple testing. The fact that results from two analytical strategies showed convergent findings, and the fact that each sample was measured twice, lend confidence to our findings. However, future confirmatory studies will be required to follow up on these results, and the present findings should be considered hypothesis-generating rather than definitive. Further, reported results come from a correlational analysis and are subject to the short-fallings of this design. Thus, it is not clear whether the observed proteomic differences represent a causal effect of early life adversity, or stem from some other factor that is correlated with early adversity but was not controlled for in this study.

To conclude, we provide evidence for altered protein expression associated with psychosocial adversity in childhood, especially implicating proteins related to mitochondrial biology, cellular energy metabolism, and inflammatory biology. The present results suggest that these effects may have some degree of sex-specificity. Lastly, network-based approaches identified adversity-related differences in protein co-expression, which largely converged with differential expression analysis on the level of single proteins. Our results identify hub proteins that can be used in directed hypothesis testing in future studies, and they support the idea of mitochondria as sub-cellular targets for the effects of adverse psychosocial influences. These data also contribute an unbiased proteomics perspective towards a rich body of work trying to elucidate how long-lasting consequences of early adverse rearing conditions are biologically sustained.

## Supplementary Information

Supplementary Information

## Data Availability

The raw data will be made available via the Open Science Framework. In the meantime, the raw data are available upon reasonable request.

## Code availability

R code for the analysis is available upon reasonable request.
