# Supplementary material for: Proteome analysis of monocytes implicates altered mitochondrial biology in adults reporting adverse childhood experiences
Source: Transl Psychiatry. 2023 Feb 1;13:31. doi: 10.1038/s41398-023-02320-w (PMC9889346; doi:10.1038/s41398-023-02320-w)
Supplement: Supplementary file 1 — Supplementary Information [file 41398_2023_2320_MOESM1_ESM.pdf]

Supplementary Information for

Proteome analysis of monocytes implicates altered mitochondrial biology in adults reporting adverse childhood experiences

Johannes C.S. Zang, Caroline May, Birte Hellwig, Dirk Moser, Marion Schwaiger, Jan G. Hengstler, Steve Cole, Markus Heinrichs, Jörg Rahnenführer, Katrin Marcus, Robert Kumsta

Corresponding authors: Katrin Marcus, Robert Kumsta

Email: Katrin.Marcus@rub.de, Robert.Kumsta@rub.de

This PDF file includes:

Supplementary Methods S1 to S10
Supplementary Results S11
Figures S1 to S22
Tables S1 to S8
SI References

Supplementary Methods 2
S1. Sample recruitment & validation of early adverse experience. 3
S2. Psychosocial stress induction: 4
S3. Blood sampling and neuroendocrine measurement: 4
S4. Monocyte isolation: 4
S5. Sample preparation for proteome analysis: 4
S6. Concentration measurement by amino acid analysis. 5
S7. Proteome profiling by mass spectrometry: 5
S8. Data handling and quality control parameters: 6
S9. Analysis of differential protein expression. 7
S10. Protein co-expression analysis 7
Supplementary Results 9
S11. Stress effects. 10
Supplementary Figures 14
Figure S1. Study design, batch design and and within running control. 15
Figure S2. Identification of protein groups. 16
Figure S3. Comparison of imputation strategies and stability of central findings. 17
Figure S4. Semantic similarities of adversity related GO-Terms. 18
Figure S5. Endocrine stress response and subjective stress experience 19
Figure S6. Stress-related changes in protein abundance. 21
Figure S7. Stress-related changes in abundance of functional specific proteins between groups. 22
Figure S8. STRING derived protein-protein interaction network for proteins upregulated in the early adversity group at t0 23
Figure S9. STRING derived protein-protein interaction network for proteins upregulated in the early adversity group at t1 24
Figure S10. STRING derived protein-protein interaction network for proteins upregulated in the early adversity group at t0 and t1. 25
Figure S11. The salmon co-expression module (consensus baseline (t0) proteome). 26
Figure S12. The purple co-expression module (consensus baseline (t0) proteome). 27
Figure S13. The light cyan co-expression module (consensus baseline (t0) proteome). 28
Figure S14. The midnight blue co-expression module (consensus baseline (t0) proteome). 29
Figure S15. Further co-expression modules identified in the consensus baseline (t0) proteome. 30
Figure S16. Protein co-expression analysis in the post-stress (t1) proteome. 32
Figure S17. The cyan co-expression module (consensus post-stress (t1) proteome). 33
Figure S18. The light cyan co-expression module (consensus post-stress (t1) proteome). 34

Figure S19. The light yellow co-expression module (consensus post-stress (t1) proteome). 35
Figure S20. The salmon co-expression module (consensus post-stress (t1) proteome) 36
Figure S21. The tan co-expression module (consensus post-stress (t1) proteome). 37
Supplementary Tables 38
Table S1: Sample Characteristics. 39
Table S2: Baseline specific DEP_a 41
Table S3: Post-stress specific DEP_a 43
Table S4. Differentially expressed proteins after stress exposure (DEP_s). 45
Table S5: Adversity group: Cortisol response proteins. 46
Table S6: Control group: Cortisol response proteins. 52
Table S7: DEP_A as referenced in MitoCarta3.0. 71
Table S8: HUB proteins as referenced in MitoCarta3.0. 72
S11 References 73

Supplementary Methods

S1. Sample recruitment & validation of early adverse experience. We recruited a sample of 60 healthy adults between 45 and 60 years of age. The study was advertised in in local newspapers and community-posted flyers. Distributed information-material asked for interest in compensated participation in a scientific study concerned with the consequences of traumatic experiences in childhood. Eligibility for study participation was evaluated during a two-step process. First, interested individuals were given information about the general study procedure, during an initial phone interview. During this interview a first set of inclusion and exclusion criteria (see below) for study participation was checked and individuals where preliminarily included into the early adversity study group based on self-reported traumatic experiences in childhood. In a second step, all potential participants were invited to the laboratory.

Participants filled in the German 28-item version of the Childhood Trauma Questionnaire (CTQ) (1; 2) to assess five categories of childhood adversity (sexual, physical and emotional abuse, and physical and emotional neglect). CTQ results were validated in a structured interview, with the Early Trauma Inventory (ETI) (3; 4). All participants were screened for mental disorders at the time of assessment or during the preceding 12 months using the Structured Clinical Interview for DSM Disorders (SKID I & II) (5) . All participants filled in the Resilience Scale (RS-25) (6) and current psychopathological symptoms were assessed with the Brief Symptom Inventory (BSI) (7). SES at present and in childhood was assessed in reference to current and childhood family income as well as the participants' and their parents' educational level. Participants that met the following criteria were excluded from study participation: Smoking (more than 5 cigarettes a day), substance addiction, intake of psychotropic or endocrine active drugs or medication, acute or chronic state of disease as well as signs for acute psychological disorders including personality disorders. For female participants, use of oral contraceptives was a further exclusion criterion and all female participants took part in the study during the luteal phase of their menstrual cycle. Based on CTQ results, we applied a cut-off score representing moderate to severe exposure for group assignment (> 12 for emotional abuse; > 9 for physical abuse; > 7 for sexual abuse; > 14 for emotional neglect; and > 9 for physical neglect). Participants in the control group scored below cut-off on all CTQ subscales. Using a matched-pair procedure, control participants were matched to the early adversity group based on sex, age and current as well as childhood socioeconomic status (SES). None of the participants fulfilled the criteria for mental disorders at the time of assessment or during the preceding twelve months. Informed consent was obtained from all subjects prior to study participation and all subjects were compensated for participation by payment of 100€. The Ethics Committee of the Albert-Ludwig's-University Freiburg (183/11) approved the study.

S2. Psychosocial stress induction: All participants underwent the Trier Social Stress Test (TSST) protocol (8; 9). The TSST is a well-validated and often used standardized 15 min protocol, specifically developed to elicit a psychosocial stress response. The TSST consist of a simulated job interview during which participants have to perform a free speech and a mental arithmetic task in front of a camera and two judge's dressed in lab coats and remaining a neutral face expression. This constructed situation of social evaluation under perceived uncontrollability (10) has been reported to reliable activate the HPA axis (11), resulting in elevated ACTH and cortisol levels. Subjective stress experience was assessed at five time points: at − 2, +1, +10, +20, and +30 min relative to stress, using the Social Emotional Response Scale (unpublished). This scale asses the dimensions arousal (calm, jittery, tense, intense, relaxed, content), self-directed emotions (guilty, ashamed, blameworthy, angry at self, dissatisfied with self), and anxiety (fearful, worried) The TSST was performed blinded with respect to group allocation.

S3. Blood sampling and neuroendocrine measurement: Blood samples for the analyses of ACTH and cortisol were drawn by an indwelling catheter at 45 and 1 min prior and 1, 10, 20, 30, 45, and 90 min post exposure to the TSST. Plasma taken at -45, -1, 1, 10, 30, 45 and 90 min was used to determine ACTH concentration. Cortisol concentration was analysed from serum collected at 45, -1, 1, 10, 30 and 90 min relative to stress exposure. Total cortisol and ACTH concentrations were measured in duplicates with an enzyme-linked immunosorbent assay (IBL, Germany) at the University of Trier and hormonal response profile were calculated based on resulting mean concentrations. As part of the initial medical examination, a standardized complete blood count was established for each participant and count of lymphocytes and monocytes was used to calculate the monocyte-to lymphocyte ratio (LMR).

S4. Monocyte isolation: $CD14^+$ Monocytes were isolated from 10 mL EDTA blood samples by immunomagnetic cell separation (MACS; Miltenyi Biotec, Germany) following a standard-operating procedure developed for the purpose of the study. A total of 10 mL Blood collected with EDTA monovettes was transferred to 50 mL Falcon tube, mixed with 500 µL of MACS® Whole Blood $CD14^+$ MicroBeads and incubated for 15 min on ice. Plasma was separated from cellular blood parts by gentle centrifugation and removed using a pipette. Remaining cell pellet was resuspended in buffer (autoMACS® Running Buffer) and magnetically labelled monocytes were isolated using a two-step-column-purification approach to maximize purity of the resulting cell fraction. 14 mL of PBS were added to the final eluate and a fraction 4 2 mL was taken after resuspension and centrifuged. Supernatant was removed and the resulting cell pellet was shock frozen in liquid nitrogen. Frozen samples were stored at -80 °C. To control for purity of the gained cell fraction, flow cytometric analyses was conducted for all samples. For this, two samples of 100 µL PBS-cell suspension, one serving as an isotype control and one for monocyte specific staining were further processed according to the SOP. Overall, cytometric analysis showed a satisfactory purity of 92.63% We found no evidence for group differences in baseline blood cell counts or monocyte isolation quality before or after stress exposure (Table S 1).

S5. Sample preparation for proteome analysis: Isolated monocytes were homogenized by a six time repeated sequence of exposure to ultrasonic waves in a pre-cooled bath for 10 seconds. followed by incubation on ice for 10 seconds. Then, physical force was applied to the cell suspension for another two minutes by use of a pestle. Resulting solution was centrifuged at 4 °C for 15 minutes at 16.000 x g. Proteins contained in the supernatant underwent further analysis. Concentration of extracted proteins was determined in duplicates by amino acid analysis as described (see below). A total of 5 µg of extracted protein was further processed in preparation

for mass spectrometry. Samples were reduced by incubation with 1,4-dithiothreitol (DTT) for 20 minutes at 56 °C and subsequently alkylated with iodacetamid (IAA) for 30 minutes at room temperature in the dark. To generate peptides, trypsin was added at an enzyme-to-protein-ration of 1:6 at 37 °C. After 4 hours, digestion was stopped by addition of 10% trifluoracetic acid (TFA) and incubation for 30 minutes at 37 °C. All samples were air-dried using a vacuum centrifuge. Peptides were resolved in 30 µL of 0.1% TFA and concentration was determined in duplicates by amino acid analysis.

S6. Concentration measurement by amino acid analysis. Concentration of extracted proteins and generated peptides was determined in duplicates by amino acid analysis(12). In short, 5 µL of protein or peptide suspension (see sample preparation) where transferred to a glass vial and dried utilizing a vacuum centrifuge. Dried residuals were hydrolysed by incubating the vials for 1 hour at 150 °C in airtight tubes under argon atmosphere after adding 6 M hydrochloric acid and one or two phenol crystals. Following, 300 µLof 20 mM hydrochloric acid were added and 10 µL of the resulting solution were mixed with 30 µL borate buffer (inclusive 10 pmol N-valin for internal calibration) and 10 µL AccQ-Tag™ derivatization reagent. Following incubation at 56 °C for 10 minutes, amino acids were separated using an ACQUITY UPLC® system and quantified measuring UV-chromatogram peak areas. Absolute amount of amino acids and subsequent protein or peptide concentrations were calculated using the N-valin peak-area.

S7. Proteome profiling by mass spectrometry: For MS-analysis 450 ng of digested protein were air dried and adjusted to a volume of 16 µL by adding 0,1% TFA. To control for performance stability and technical variance, we measured 22 external (EC) and 14 sample-derived (SC) control standards additionally. Each SC sample contained a 450 ng mixture of peptides reassembled from equal parts of all 118 participant samples. The samples running order was developed to avoid sequence effects. Batch design was set as following: First, all samples were matched by gender and age. Second, samples were assigned to clusters of four, representing pre and post-stress measurements of two participants of similar age and different sex. Control standards were measured prior to every cluster. LC-MS/MS proteome analysis was conducted utilizing an QExactive™ hybrid quadrupole-Orbitrap mass spectrometer (Thermo Scientific) equipped with a Ultimate 3000 RSLCnano LC-system (Dionex) coupled to a nano-electrospray ion source (Thermo Scientific). Peptide mixtures were loaded on a 2 cm Acclaim PepMAP ® 100 microcapillary column (Thermo Scientific) packed with 5 µm C18 resin followed by 50 cm Acclaim PepMAP ® RSLC microcapillary column (Thermo Scientific) packed with 2 µm C18 resin by an auto sampler at a flow rate of 30 µL/ min at 60 °C and separated using a 120 min gradient of 5–40% acetonitrile gradient in 0.1% formic acid. Gradient was started with a mixture of 95% A (0.1% formic acid) and 5% B (84% acetonitrile, 0.1% formic acid). Subsequently B was increased over time to a concentration of 40%. In each data collection cycle, one full MS scan (350–1.400 m/z) was acquired in the Orbitrap with 70.000 resolution setting, an automatic gain control (AGC) setting of 3 x e6 and a maximum ion accumulation time of 80 ms. The subsequent MS2 analysis was performed with a top 10 setting. Most abundant ions were selected for fragmentation by high-energy collision induced dissociation (HCD). HCD was performed with a collision energy of 27%, an AGC setting of 1 x e6, an isolation window of 2.2 m/z and a maximum ion accumulation time of 120 ms. Previously analysed precursor ions were dynamically excluded for 30 sec. Fragment ion analysis was performed in an Orbitrap analyser with a resolution of 35.000 at 200 m/z, target of 1e6 and an accumulation time of 120 ms. After each run, columns were equilibrated using two washing steps, a short one followed by a washing step of 50 min.

Process performance was controlled in real-time by monitoring running parameters, chromatograms and protein-identification rates. For the latter, Thermo Raw files were analysed by means of the Proteome Discoverer v1.4. (Thermo Fisher Scientific, Bremen) software. We performed automatic spectra interpretation for peptide identification using MASCOT™V.2.2 (Matrixscience, London, UK) on an in-house Mascot server as soon as acquisition was completed. Enzyme specificity was set to trypsin, and two missed cleavages were tolerated. Carbamidomethylation of cysteine was set as a fixed modification and oxidation of methionine as a variable modification. The precursor ion mass tolerance was set to 5 ppm, and the product ion mass tolerance was set to 0.4 Da. As references for protein sequences, UniProt/SwissProt decoy database (release 2015_05) with a restriction to "Homo sapiens" (http://www.uniprot.org) was used. FDR threshold was set to 1%. Identification rates of >2000 proteins were applied as a threshold criteria indicating an acceptable analysis performance. Altogether 34 samples were measured repeatedly and selected for inclusion based on given identification rates.
To identify the most common peptide modifications present in our samples we performed a Preview (13) analysis on a subset of raw files of 10 samples and 13 IC samples. Raw files were searched against a complete UniProt protein database provided in in FASTA format. Carbamidomethylation was set as a fixed Cysteine modification, RK was selected as trypsin specific C-terminal cleavage site and a fully specific search was conducted with fragmentation type set to CID/HCD. Based upon Preview results we included Carbamidomethylation (+57.0215 Da) of cysteine as a fixed modification and oxidation of methionine (+15.9949 Da) as well as N-terminal conversation of glutamate to pyroglutamate (-17.02655 Da) as variable modifications when finally processing MS Raw data using Max Quant (v.1.5.3.30) with its integrated search engine Andromeda (14; 15). All further Max Quant settings represented software defaults. Max Quant analysis was performed separately for samples and controls. Respectively, data were normalized using the MaxLFQ algorithm implemented in Max Quant (15) over all samples and all subsequent analyses are based upon normalized LFQ-Data.

S8. Data handling and quality control parameters: Proteins were accepted as identified by LC-MS/MS and used in correlational and differential expression analysis when identified with a) at least two unique peptides and b) with valid values in 80% of a specific condition or in at least 80% in the of one of the conditions compared. These criteria where applied to increase validity of measurement on the one hand and simultaneously allow for possible on-off regulations. The 80% cut-off criterion results from curve inspection (Figure S3), is close to the turning point and represents a reasonable trade-off between a strict threshold and acceptable identification rates. Missing values are a challenge for every proteome analysis (16). Although in the data used for further analysis missing values seemed rather independent from abundance (Figure S4), missings might not be completely at random. Thus, we applied Deterministic Minimum Imputation (MinDet) that has been shown to be suitable for imputation of MNAR values on the protein level (17). To control for artificially introduced biases due to value imputation, we compared results from analyses based on data sets in which missing values were either a) replaced by zero (log2(x+1), b) regarded as NA value, c) replaced by a global intensity minimum or d) replaced by a protein specific minimum. Results differed slightly depending on how missing values where treated. However based on differential analyses, we detected protein subsets that found stable abundance differences between groups independent from the imputation method applied (Figure S.4). To avoid restriction of biological variance due to global imputation subsequent statistical analyses were carried out on data in which missing values were imputed through a protein specific minimum. Evidence level of identified proteins is indicated by the parameter Q representing the percentage of valid values given in the sample for the respective protein. In graphical representations, the evidence level is color coded with yellow indicating the eighty percent criterion and red indicating proteins identified by a hundred percent of valid values. Proteins not meeting the eighty percent criterion as a minimum requirement are colored in grey.

S9. Analysis of differential protein expression. Analysis of stress and trauma related differential protein expression was performed on log2 –transformed LFQ-Data using the limma package(33). This type of analysis is based on parametric empirical Bayes and includes global variability in the variance estimation, thus ensuring reliable and stable inference even in smaller samples. Significance level for differential analysis was set to 5%. All reported effects are adjusted for the variable sex. Based on technical variation found within IC samples, cut off for meaningful fold changes was set to FC =1.3. Technical variance was calculated on LFQ Data of proteins identified in 100% of all IC samples by dividing LFQ means through the standard deviation. Resulting coefficient of variation was 10%. Gene-ontology (GO) enrichment analysis was performed utilizing Enrichr(34) and adjusted Fishers exact test. Further, to build up protein-protein interaction networks UniProt accessions were supplied to the STRING database(35), with a combined STRING score of 40.4 (i.e. medium-to-high confidence interactions) as confidence parameter. Next, we tested enrichment of DEP_a sets against the monocytes whole proteome universe using the topGO package(36) and used semantic similarity analysis(37) to estimate functional similarities between derived GO-Terms. To account for graph location and the relation with ancestors terms semantic similarities were calculated using Wangs(38) method followed by cluster analysis to group GO-Terms based on similarity scores.

S10. Protein co-expression analysis. Network analysis was performed on a consensus set of proteins identified by at least 80% of valid values (n_{prot} = 1119) in adversity and control group participants before and after stress exposure using the WGCNA package(32) to detect modules of highly co-expressed proteins and analysed stress-related module preservation or disruption. We compared module expression differences between sex and condition using linear models based on calculated eigenvalues (eigenproteins) of each module (MEs) that were further correlated with clinical variables. Module preservation analyses across stress induction were carried out using a permutation-based preservation statistic with 500 random permutations of the data(39). Modules were tested for protein enrichments using the in-package ImmunePathwayLists, brainLists, BloodAtlases, and CHDI lists for enrichment comparison. Resulting p.values were corrected for multiple testing through the Bonferroni method. Module specific UniProt accessions were supplied to the Enrichr environment and the STRING database to gain further insights into module related biological processes (settings as reported above). For each module, we defined hub proteins (highly connected proteins) based on their intramodular kME (the correlation between each protein and each modules eigenprotein). First we calculated the kME values as indicator of the protein - ME relationship across all proteins of the consensus data set, and all baseline modules separately for pre- and post-stress protein identifications. Second, we selected proteins that displayed the highest kME values for each module in both, baseline and post-stress data sets. Thus, conserved hub proteins are characterized by high correlation between the baseline and post-stress data set and strong module relation. They contain predominately but not exclusively proteins previously assigned to the respective module. We report both, top ten and top ten percent of proteins with the highest kME in pre- and post-stress networks. Subsequently, we fitted multiple regression models using the R package variancePartition(40) to the identified hub proteins. The aim was to characterize the percent of protein variance explained by variables of interest; including participants CTQ total score, age, sex, or BMI.

Supplementary Results

S11. Stress effects.

On the endocrine level, psychosocial stress exposure led to significant hormone increases, with an attenuated ACTH and a significantly lower cortisol response in the early adversity group (Figure S5 A-D), an increased subjective stress experience was observed on the dimensions self-directed emotions, tense arousal, and anxiety (Figure S5 E-G). On the proteome level, we observed minor effects of stress on the overall variability of group-specific protein expression with high correlations of variation before and after stress exposure in the total of detected protein groups (Figure S6 C-E).

Limma analysis was used to investigate the influence of stress on protein abundance in participants with a history of childhood adversity and control individuals. We identified altogether eight differentially expressed proteins after stress exposure (DEP_s) (Figure S6 L & M, Table S4). All DEP_s were up-regulated at the post-stress measurement (t1) 180 min after stress exposure, with an exception of LTF that exerts i.a. antimicrobial and antiviral activity within innate immune functioning. In both conditions, DEP_s include proteins functionally related to adaptive immune processes and wound healing, e.g. FGA, FGB and FGG, whose up-regulation might represent a molecular resonate of the blood sampling procedure. Specifically up regulated in control individuals, ILK and PSMD1 function within the MAPK signaling pathway are involved in immune response related signal transduction, and stress-related maintenance of the cells proteome. The MAPK pathway mediates cellular stress, inflammation processes, and modulates the expression of the glucocorticoid receptor. Further, HK1 as an enzyme attached to the mitochondrial outer membrane is crucial for glucose metabolism, catalyzing the initial and rate-limiting first step in the cells main process of ATP generation (Table S4).

The subsequently performed variance partitioning analyses widely corroborated these findings. After controlling for the influence of other variables, including participants' sex, age, CTQ and RS25 scores, LMR, BMI, WHR, smoking and drinking habit as well as cortisol and ACTH base-to-peak values, the exposure to psychosocial stress explained up to 11% of variance in abundance of distinct proteins within both groups but did not support an overall effect of stress on protein expression. Altogether, stress accounted for more than 5% of variance in expression of respectively 23 proteins derived from participants with a history of childhood experience or control individuals and with an exception of FGA and FGB, both of these stress-related protein sets contained different analytes.
Of all stress-related proteins derived from the adversity group, stress explained most of variance in expression of FGA (10.6%), FGG (10.5%), CAB39 (10.5%) and SNX27 (9.25%). Within the control group stress accounted for the most variance after in G3BP1 (10.8%), SMAP2 (10.6%), SEPT9 (7.5%) and CCAR1 (7.4%) after controlling for the influence of all other variables. EnrichR analyses were performed on both protein sets and showed that the 23 stress-related proteins derived from either the adversity or the control group were both significantly enriched for GO-Terms associated with blood coagulation processes such as e.g. indicated an involvement in blood coagulation processes plasminogen activation (GO:0031639) or platelet aggregation (GO:0070527). In addition, particular stress-related proteins derived from control individuals were further associated with GO-Terms indicating an involvement in antimicrobial processes (GO: 0019731) and apoptotic signaling (GO: 1902042). Variance partitioning analyses were further conducted upon the 1119 proteins comprising the consensus data set in order to investigate to what extend stress-related changes in protein expression depend on participants' group (group x stress interaction). This interaction effect did not explain substantial parts of variance across all proteins and accounted in the following three proteins for more than 5% of variance: SMAP2 (6.7%), TPR (5.5l) and OLA1 (5.1%).

Analysis of adversity-related differences in protein abundance suggested mitochondria and immune related proteins as targets of interest and we interrogated stress effects regarding protein changes in these domains as well as hemostasis. Comparing stress-related fold changes o protein levels between the early adversity and control group, we observed substantial positive correlations of immune associated (r= 0.69, p< .001) and stress associated (r= 0.67, p< .001) proteins and moderate correlations of group specific fold changes in proteins playing a role in mitochondrial organization (r= 0.27, p< .001) (Figure S6 F-K).

Variation in post-stress (t1) protein expression was in general higher in the control group and compared to background changes more strongly correlated between groups in immune and stress-related proteins as well as in proteins functioning in vesicle-mediated transport or associated with secretion. In contrast, stress specific changes in proteins related to mitochondrial organization, the cells energy metabolism or cellular respiration (Figure S7) were less pronounced and rather weakly correlated between groups, which might indicate group-specific regulation of proteins involved in mitochondrial biology and the cells energy metabolism. Further, we investigated the association between participant's cortisol response and stress-related changes in protein abundance using Spearman's rang (Figure S6 A & B) and report the respective findings in Table S5 & S6.

Next, WGCNA was conducted upon the post-stress (t1) consensus proteome to investigate the relation between protein co-expression and early stressful experiences following stress. The resulting consensus network (Figure S16) comprised altogether nine modules. Five of these modules (the cyan module, the light cyan module, the light yellow module, the salmon module and the tan module) showed noticeable expression differences between male participants with a history of childhood adversity and control individuals. While all these five modules appeared as rather conserved within baseline co-expression network ($Z_{summary}$ > 5), overall differences between groups became only evident in expression of the salmon and the light-yellow module.

As such, the post-stress salmon module comprised 82 proteins which were among others associated with neutrophil degranulation (GO: 0043312) neutrophil mediated immunity (GO: 0002446) and viral process (GO: 0016032). This module contained altogether 27 DEPa and shared 70 proteins with the baseline salmon module and three proteins with the baseline purple module. STRING derived protein-interaction networks showed distinct clusters of related proteins functionally involved in e.g. ATP metabolic processes, protein transport, and viral processes (Figure S8). Eigenproteins of the post-stress salmon module were positively correlated with particpants history of childhood adversity (r = 0.31, p< .05), participants CTQ total score (r = 0.30, p< .05) as well as participants'' scores on the CTQ subdimensions physical neglect (r = 0.31, p< .05) and emotional neglect (r = 0.29, p< .05). Further, expression of this module reflected largely expression of the baseline salmon module.
Thus, the experience of childhood adversity (β = .25, p< .05), but not participants'' sex (β = - 0.18, p=.10) predicted post-stress module expression F(3,55)= 3.01, p< .05, R2 = .094.
Group comparisons supported these findings and indicated significant higher expression of the salmon module in participants' with a history of childhood adversity F(1,55)= 8.13, p<.01, η = .13. There was no overall difference between female and male participants F(1,55)= 0.42, p=.52, η = .008 that met statistical significance and no significant interaction between participants' group and sex F(1,55)= 2.31, p=.13, η = .04. Yet, plotting eigenproteins stratified by group and sex showed particularly pronounced expression levels in male participants with a history of childhood trauma in comparison to male control individuals t(17) = 3.47 ,p<01. After controlling for the influence of other covariates, such as e.g. participants sex, age, BMI as well as CTQ & RS25 scores, participants`` CTQ score accounted for 12.9% of variance in the salmons´ module expression.

The post-stress light yellow module contained altogether 73 proteins. 49 of these proteins were shared with the baseline purple module, two were shared with the baseline light cyan module and 23 of the modules proteins were identified as differentially expressed between groups (DEPa) in baseline or post-stress comparisons or at both time points. GO Terms associated with proteins of the light yellow module implicated a functional involvement in mediation of immune processes (e.g. neutrophil degranulation (GO:0043312) or neutrophil mediated immunity (GO:0002446)), and protein translocation processes (e.g. protein transport (GO:0015031) or cellular protein transport (GO:0034613)).Further STRING derived protein cluster were functionally related to protein secretion, protein transport an immune response and there was a distinct cluster of proteins involved in mitochondrial functioning. Expression of the post-stress light yellow module correlated positively with the experience of childhood adversity ($r = 0.27$, $p < .05$), participants' CTQ total score ($r = 0.27$, $p < .05$) as well as the scores on the CTQ subdimensions physical neglect ($r = 0.30$, $p < .05$) and physical abuse ($r = 0.32$, $p < .05$). Correlational analyses further indicated a relation between module expression and participants' sex ($r = -0.34$, $p < .01$) and showed higher module expression rates in participants with higher RS25 scores ($r = 0.36$, $p < .01$). Both, a history of childhood adversity ($\beta = -.07$, $p < .05$) and participants sex ($\beta = .09$, $p < .05$) predicted expression of the post-stress light yellow module $F(2,56) = 6.77$, $p < .01$, $R2 = .16$ and group comparisons supported both, an overall effect of group indicating a higher module expression in participants with a history of childhood adversity $F(1,55) = 5.10$, $p < .05$, $\eta = .08$ and sex, indicating an overall lower expression rate in male participants $F(1,55) = 8.42$, $p < .01$, $\eta = .13$. There was no significant interaction between both factors $F(1,55) = 0.10$, $p = .75$, $\eta = .753$. After controlling for further variables using variance partitioning analysis, participants' CTQ total score accounted for 5.28% participants sex accounted for 3.55% and participants' RS25 scores accounted for 3.7% of variance in module expression.

The post-stress cyan module contained 52 proteins functionally related to protein targeting processes (e.g. GO: 0006613), peptide biosynthetic processes (GO: 0043043) or viral gene expression (GO: 0019080). None of the modules proteins were differentially expressed between groups and the post-stress cyan module shared proteins with the baseline purple module (n = 2), the baseline light cyan module (n = 12) and the baseline midnight blue module (n = 7). Derived STRING networks showed clusters of proteins associated with translation initiation, RNA processing, gene expression, and leukocyte activation. There was no significant correlation between this modules expression and any of the participants' characteristics. Although male participants did show significant higher module expression levels compared to male control individuals (17) = 3.47, p<01, neither childhood adversity ($\beta = 0.21$, $p = .10$) nor sex ($\beta = 0.22$, $p = .051$) predicted module expression across all participants $F(3,55) = 1.656$, $p = .19$, $R2 = .031$. Further, group comparisons did not show overall differences between groups $F(1,55) = 1.26$, $p = .022$, $\eta = .04$ or male and female participants $F(1,55) = 2.00$, $p = .163$, $\eta = .035$) and there was no significant interaction explaining variation in expression of the cyan module $F(1,55) = 2.38$, $p = .128$, $\eta = .042$. After controlling for all other variables, variance partitioning analyses showed that participants' CTQ score accounted for 1.7% of variance observed in the post-stress cyan modules expression.

The post-stress light cyan module contained 83 proteins. 51 of these proteins where shared with the baseline light cyan module and 12 of these where assigned to the midnight blue module in baseline analyses. GO-Term associations linked proteins of the light cyan module mainly to mRNA processes (e.g. GO: 0000398, GO: 0006396). STRING networks based on these proteins corroborated this associations and distinct protein-protein interaction clusters involved in e.g. mRNA splicing and protein transport. The light cyan modules expression was negatively correlated with participants' score on the CTQ physical neglect subdimension. The experience of

childhood adversity ($\beta = 0.25, p<.05$) but not participants' sex ($\beta = 0.18, p=.011$) predicted expression of the light cyan module $F(3,55)= 1.515, p= .22, R2 = .025$. Although male participants' with a history childhood adversity showed significant lower module expression $t(17) = 2.34$,$p<.05$, variance analyses did neither support an overall effect of group $F(1,55)= 3.12, p=.083, \eta = .054$ or sex $F(1,55)= 0.03, p=.085, \eta < .01$, nor an significant interaction of both these factors $F(1,55)= 3.03, p = .086, \eta = .05$.

Finally, 89 co-expressed proteins built up the post-stress tan module. 55 of these proteins were present in the baseline light cyan module and 17 of these proteins were shared with the baseline midnight blue module. The post-stress tan modules proteins where among others, functionally associated with e.g. protein modification processes (e.g. GO: 0016579, GO: 0070646) or molecular processes involved the processing and presentation of exogenous peptide antigen via MHC class I (GO: 0042590). Further, STRING derived network clusters indicated a functional involvement in regulation of cytoskeleton organization, leukocyte activation, protein deubiquitination and mRNA processing.

Expression of the post-stress tan module was negatively associated with participants' history of childhood adversity ($r = 0.26, p= .05$) and correlated significantly negatively with participants' BMI ($r = -0.27, p< .05$) and participants' score on the CTQ physical neglect subscale ($r = -0.45, p< .01$). Both, the experience of childhood adversity ($\beta = -.52, p<.01$) and sex ($\beta = 0.34, p<.011$) significantly predicted expression of the post-stress tan module, as did the interaction of group and sex ($\beta = -0.17, p<.05$) $F(3,55)= 3.92, p < .05, R2 = .11$. Variance analysis showed overall expression differences between participants with a history of childhood adversity and controls $F(1,55)= 7.90, p<01, \eta = .13$ but no overall effect of participants sex $F(1,55)=.001, p= .99, \eta <.01$. Particular male participants with experiences of childhood adversity showed reduced expression of the post-stress tan module (Figure S16) reflected by a significant interaction effect of group and sex $F(1,55)= 5.79, p<.05, \eta = .010$. After controlling for the influence of all other variables, participants' CTQ total score accounted for 7.3% of variance in expression of the post-stress tan module.

To investigate the effect of stress on protein co-expression more directly, WGCNA was further performed upon the consensus proteome, a set including both, the baseline and post-stress derived protein expression levels of the consensus proteome ($n = 1119$). The emerging network harbored altogether five modules of co-expressed proteins. We did not observe significant correlations between the point of measurement (baseline and post-stress) or participants'' cortisol and ACTH levels and any of the modules eigenproteins. Similar, neither stress, nor the interaction between stress and group did explain noticeable parts of variance in module expression after controlling for the influence of other variables. Yet, three of the identified modules were significantly correlated with the experience of childhood adversity as well as participants' CTQ total score. These modules tended to share proteins with adversity associated co-expression modules identified before and after the exposure to psychosocial stress, which further points to the presence of stress-independent but trauma-associated protein expression profiles.

Supplementary Figures

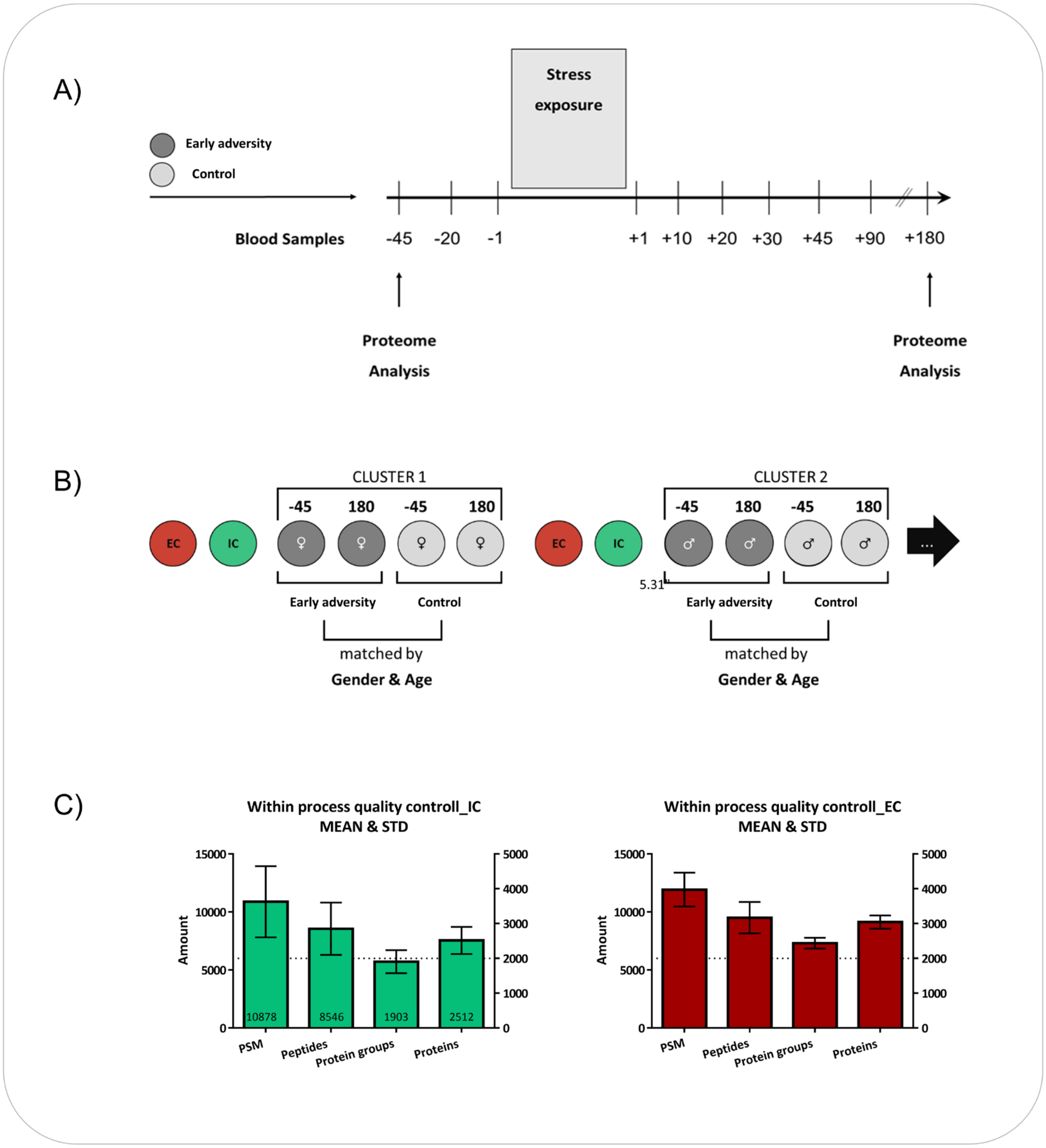

Figure S1. Study design, batch design and within running control.
Shown is the experimental time course, with sequential blood collection (A). All participants underwent the TSST. Monocytes isolated from blood taken at the baseline measurement (t0) 45 min before and at the post-stress measurement (t1) 180 min after stress induction were used for proteome analysis. Based on the study design, the sample running order was developed (B). Peptide samples were clustered according to condition and time and matched by gender and age. Internal and external control samples were measured before and after every cluster. Resulting parameters from MS analysis of internal (C) and external (D) control samples are depicted by means of a bar plot representing mean values and standard deviation.

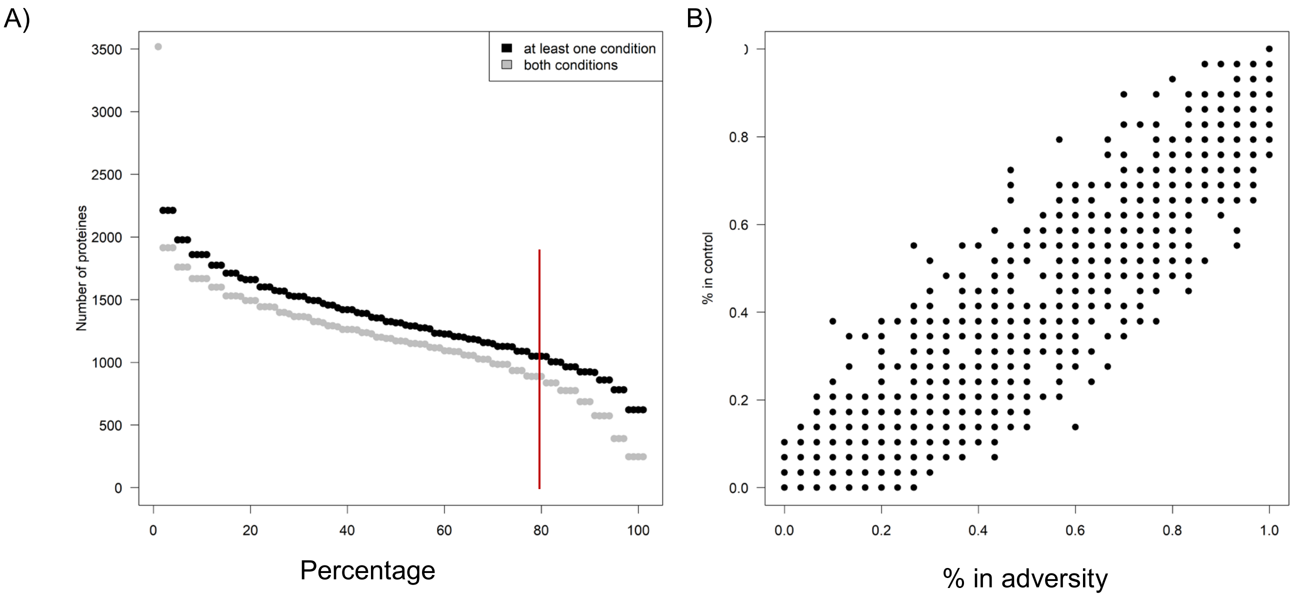

Figure S2. Identification of protein groups.
Number of protein groups identified in at least one or both of compared groups (adversity vs. control) (A). Protein groups that were identified by valid values in the early adversity group were likely to show the same identification ratio in the control group (B).

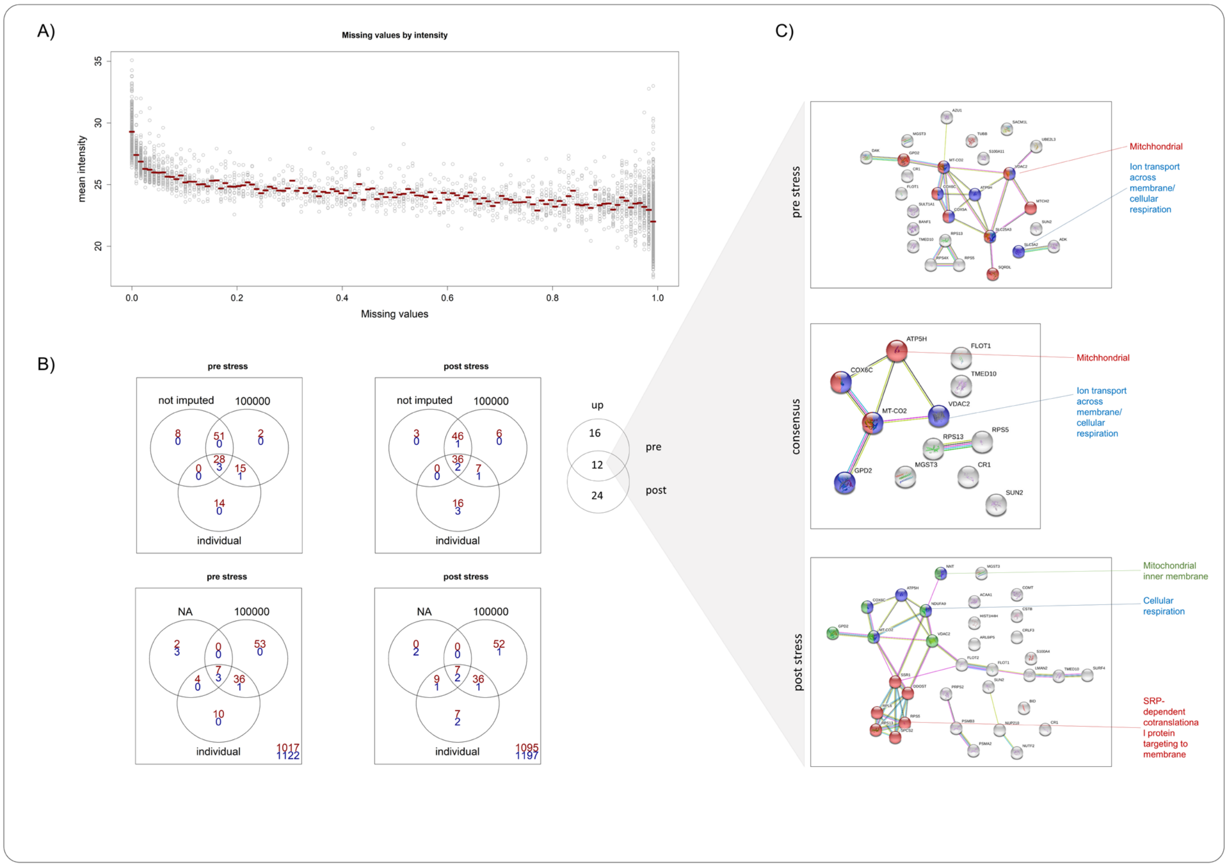

Figure S3. Comparison of imputation strategies and stability of central findings.
Missing values plotted against mean protein intensity (A). Missing values were imputed either by a) zero (log2(x+1), regarded as NA value, replaced by a global intensity minimum or replaced by a protein specific minimum. Venn diagram depict overlap in DEP$_a$. Red marks proteins with higher abundance in the early adversity group, blue marks proteins with higher abundance in the control group. Stable expression differences between groups independent from imputation method became evident in adversity associated proteins before stress (n_{prot} = 28) and after stress (n_{prot} = 38). Protein-interaction networks were generate form these protein sets and intersection of both (n_{prot} =12). In all networks, proteins related to cellular respiration and mitochondrial functioning formed interconnected structures, showing that central results were stable across imputation methods.

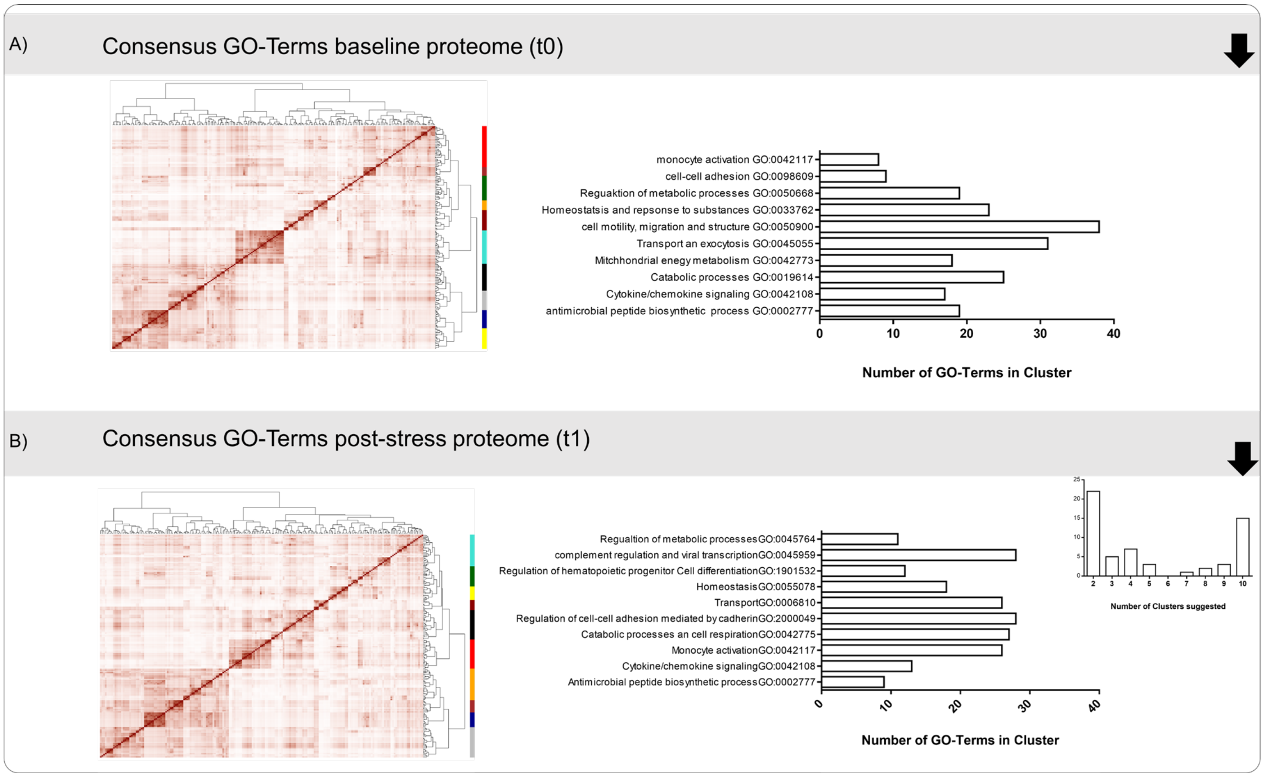

Figure S4. Semantic similarities of adversity related GO-Terms.
Semantic similarities of DEP_a associated GO-Terms were calculated using Wangs (18) method. Clustered semantic similarity scores are represented as heat maps, bands indicate ten cluster solutions. A ten cluster solution was suggested by the majority of fit indices (19) for the baseline network and applied as well to the post-stress data set. Number of GO-Terms comprised in each cluster are depicted in the bar plot and each cluster is labelled by one representative GO-Term.

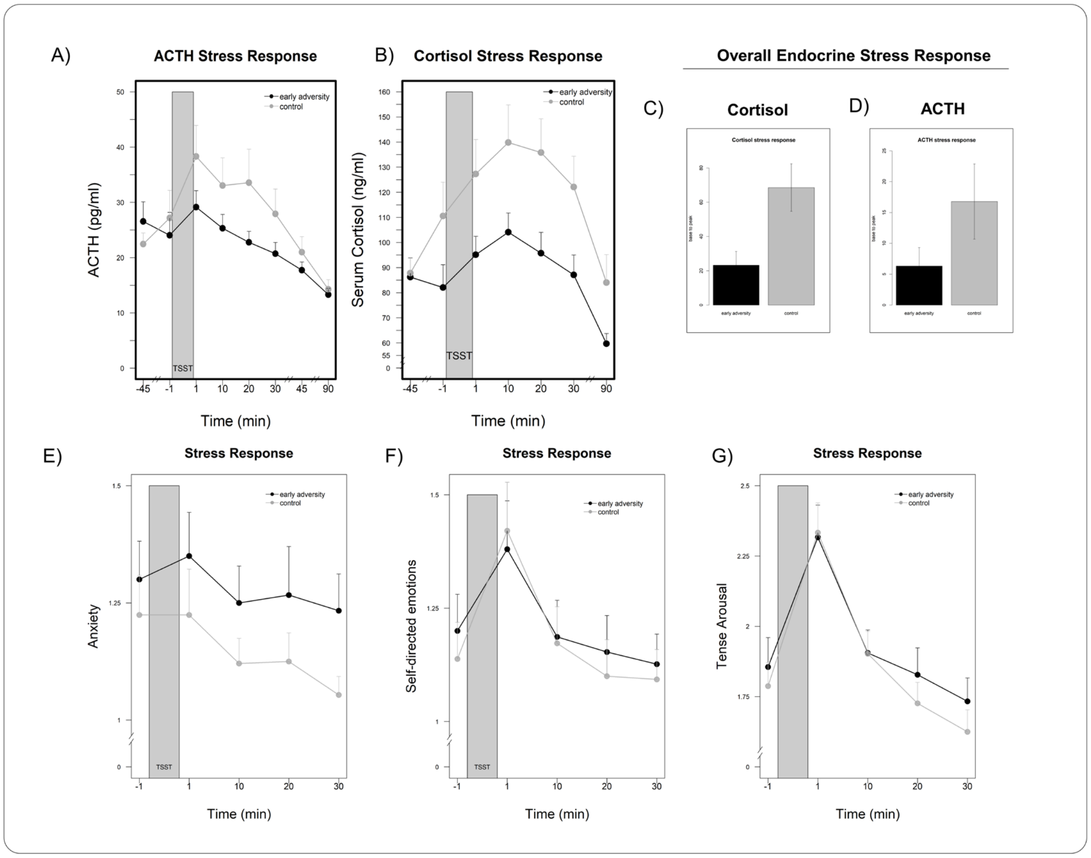

Figure S5. Endocrine stress response and subjective stress experience

Exposure to the Trier Social Stress Test led to significant increases of ACTH and cortisol in both groups (main effect time: ACTH: F(2.07,105.53) = 11.89, p< 0.001, η^2 = 0.9, Cortisol: F(3.28,183) = 21.88, p< 0.001, η^2 = 0.9).The early adversity group displayed an attenuated ACTH and a significantly lower cortisol response compared to the control group (A and B: ACTH: main effect group: F(1,51) = 0.89, p = 0.35, η^2 = 0.01, interaction time by group: F(2.07,105.53) = 2.54, p = 0.08, η^2 = 0.02. Cortisol: main effect group: F(1,56) = 5.16, p = 0.03, η^2 = 0.7; interaction time by group: F(3.28,186.96) = 3.13, p = 0.02, η^2 = 0.01). Participants with a history of childhood adversity had lower ACTH (D: base- to-peak response MT = 6.31 (2.99), MC = 16.78 (6.08), t(57) = 1,56, p= 0.12) and significant lower cortisol (C: base- to-peak response MT = 23.27 (8.02), MC = 68.56 (13.83), t(57) = 2,85, p < 0.01, d = .74) responses. Error bars and values in brackets represent SEM. Following stress exposure, participants reported higher levels of self-directed emotions, ,tense arousal and anxiety (A-C), Self-directed emotions: main effect condition: F(1,56) = 0.84, p = 0.84, η2 = 0.0005; main effect time; F(1.52,84.91) = 12.79, p< .0001, η2= .06; interaction effect conditon:time: F(1.52, 84.91)=0.47, p= 0.57, η^2= .002; Tense arousal: main effect condition: F(1,56) = 0.23, p= 0.63, η^2= 0.002; main effect time; F(2.62,146.94) = 28.18, p< .0001, η^2 = 0.17; interaction effect conditon:time; F(2.62,146.94) = 0.40, p = 0.72, η^2 = 0.003; Anxiety: main effect condition: F(1,56,1.69), p = 0.20, η2 = 0.02; main effect time; F(3.17,177.41) = 3.17, p = 0.02, η2 = 0.02; interaction effect conditon:time: F(3.17,177.41) = 0.36, p = 0.80, η^2 = 0.02).

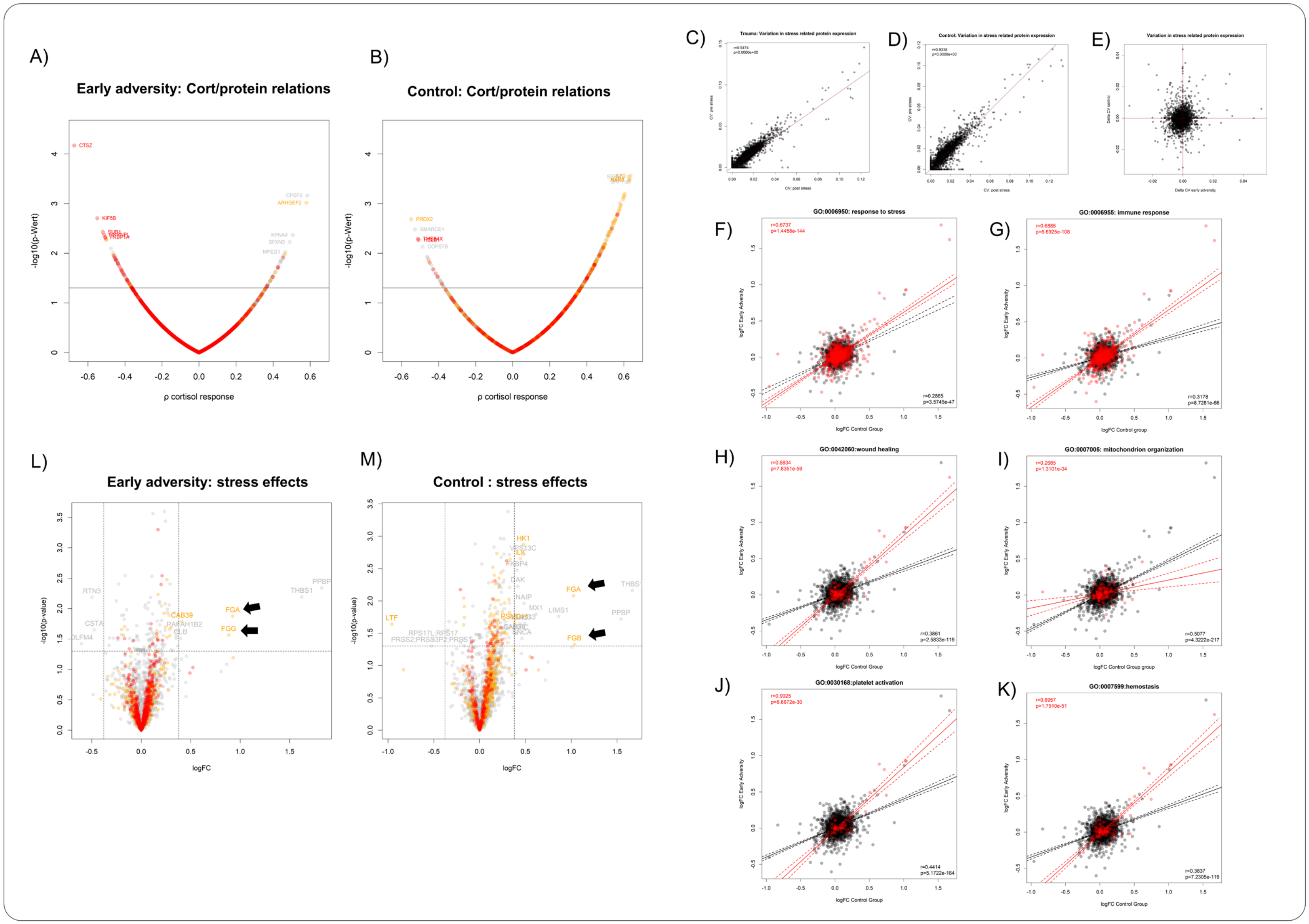
A)
Early adversity: Cort/protein relations
-log10(p-Wert)
ρ cortisol response
B)
Control: Cort/protein relations
-log10(p-Wert)
ρ cortisol response
C)
Trauma: Variation in stress related protein expression
D)
Control: Variation in stress related protein expression
E)
Variation in stress related protein expression
F)
GO:0006950: response to stress
G)
GO:0006955: immune response
H)
GO:0042060:wound healing
I)
GO:0007005: mitochondrion organization
J)
GO:0030168:platelet activation
K)
GO:0007599:hemostasis
L)
Early adversity: stress effects
-log10(p-value)
logFC
M)
Control : stress effects
-log10(p-value)
logFC

Figure S6. Stress-related changes in protein abundance.

Spearman's correlation between stress-related differences in protein abundances and participants' cortisol response in the early adversity and control group are depicted as volcano-plots (A & B). Across all detected protein groups, variation in protein expression before and after stress exposure was highly correlated in the early adversity ($r(3517)=.95$, $p <.001$) and the control group ($r(3517)=.94$, $p <.001$), while changes variability over time were mostly uncorrelated between conditions (C-E). Relation of stress induced fold change differences between groups for stress (F), immune (G) and wound healing (H) associated proteins as well as proteins functioning within mitochondria (I) platelet aggregation (J) and hemostasis (K) Red dots represent proteins associated with the respective GO-Term, black dots represent background expression changes. Further, volcano plots compare the extent of $\log_2$ fold-changes (pre vs. post-stress) and − log10 p-value significance for protein in the early adversity (L) and the control group (M). Dashed lines show the |1.3 | FC cut-off criteria (vertical) and the .05 significance threshold (horizontal). Black arrows mark FGA, FGG and FGG as hemostasis related proteins. Colors in volcano plots reflect the 80% (yellow) or 100% (red) validity threshold.

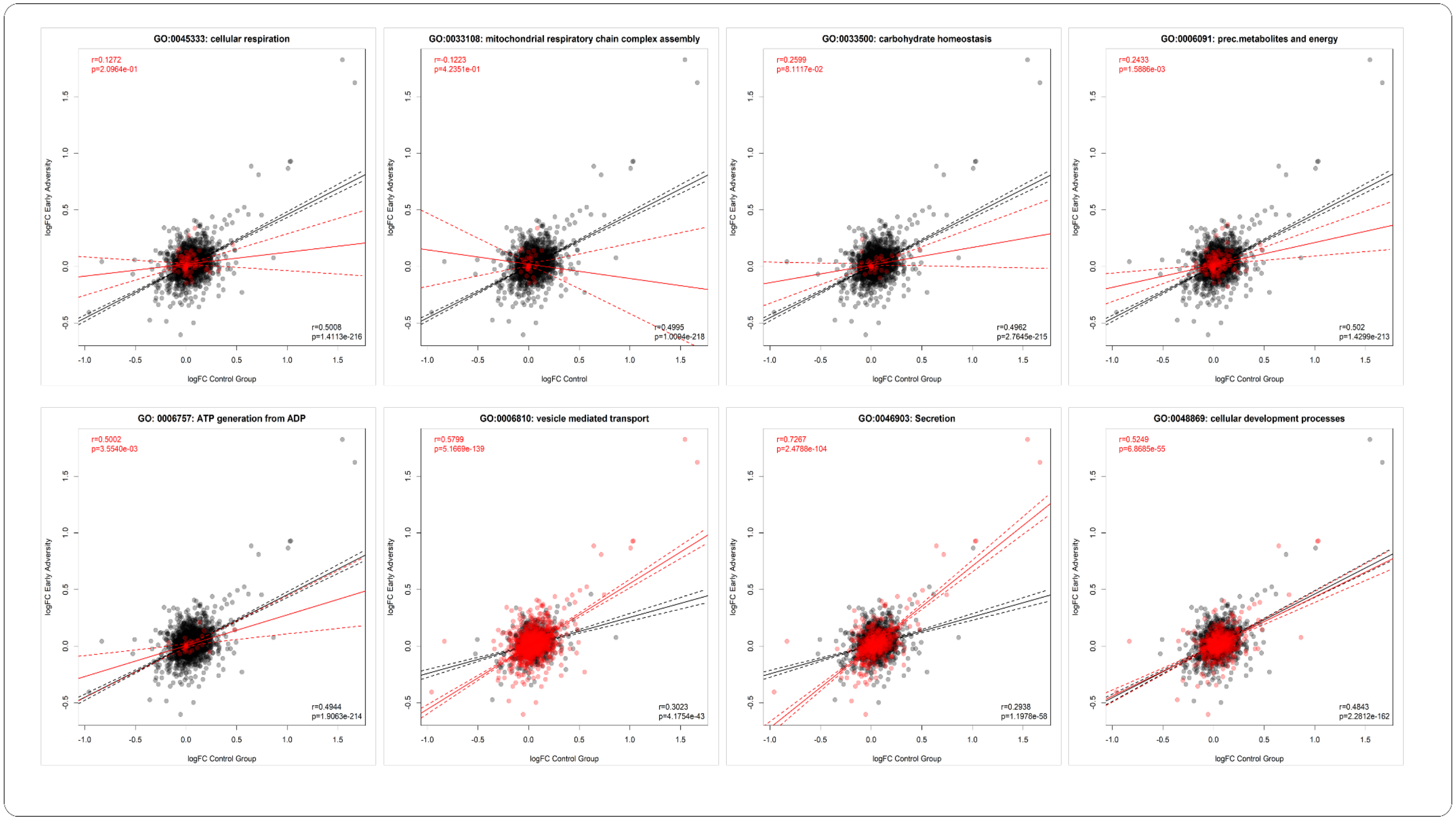

Figure S7. Stress-related changes in abundance of functional specific proteins between groups. Relation of stress induced fold change differences between groups for proteins with a relation to cellular energy metabolism, immune functions and cellular development processes. Red dots represent proteins associated with the respective GO-Term, black dots represent background expression changes.

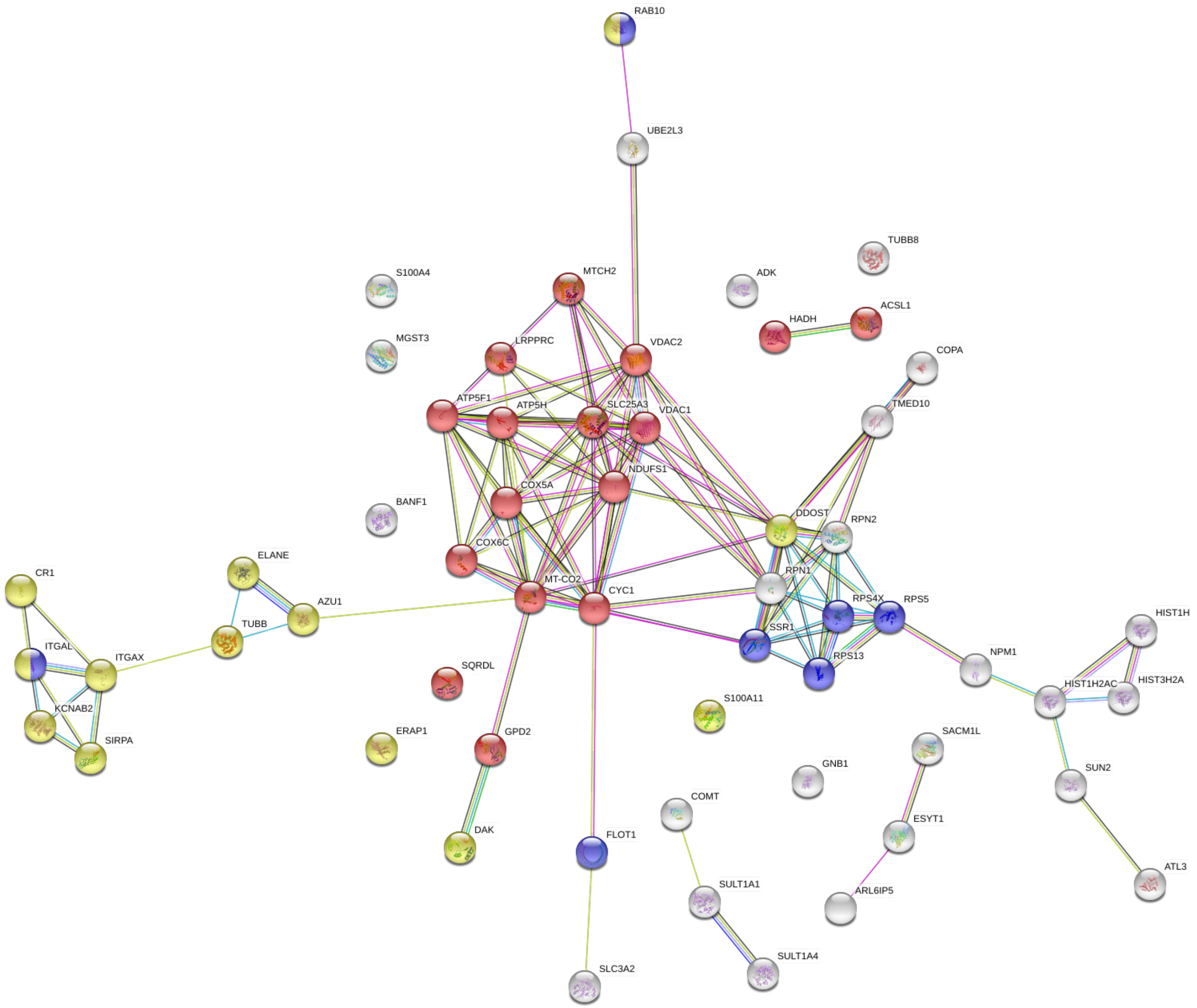

Figure S8. STRING derived protein-protein interaction network for proteins upregulated in the early adversity group at t0. Proteins colored in red are associated with mitochondrial biology, and proteins in blue are related to regulation of protein synthesis and transport. Yellow proteins relate to immune processes and neutrophil degranulation.

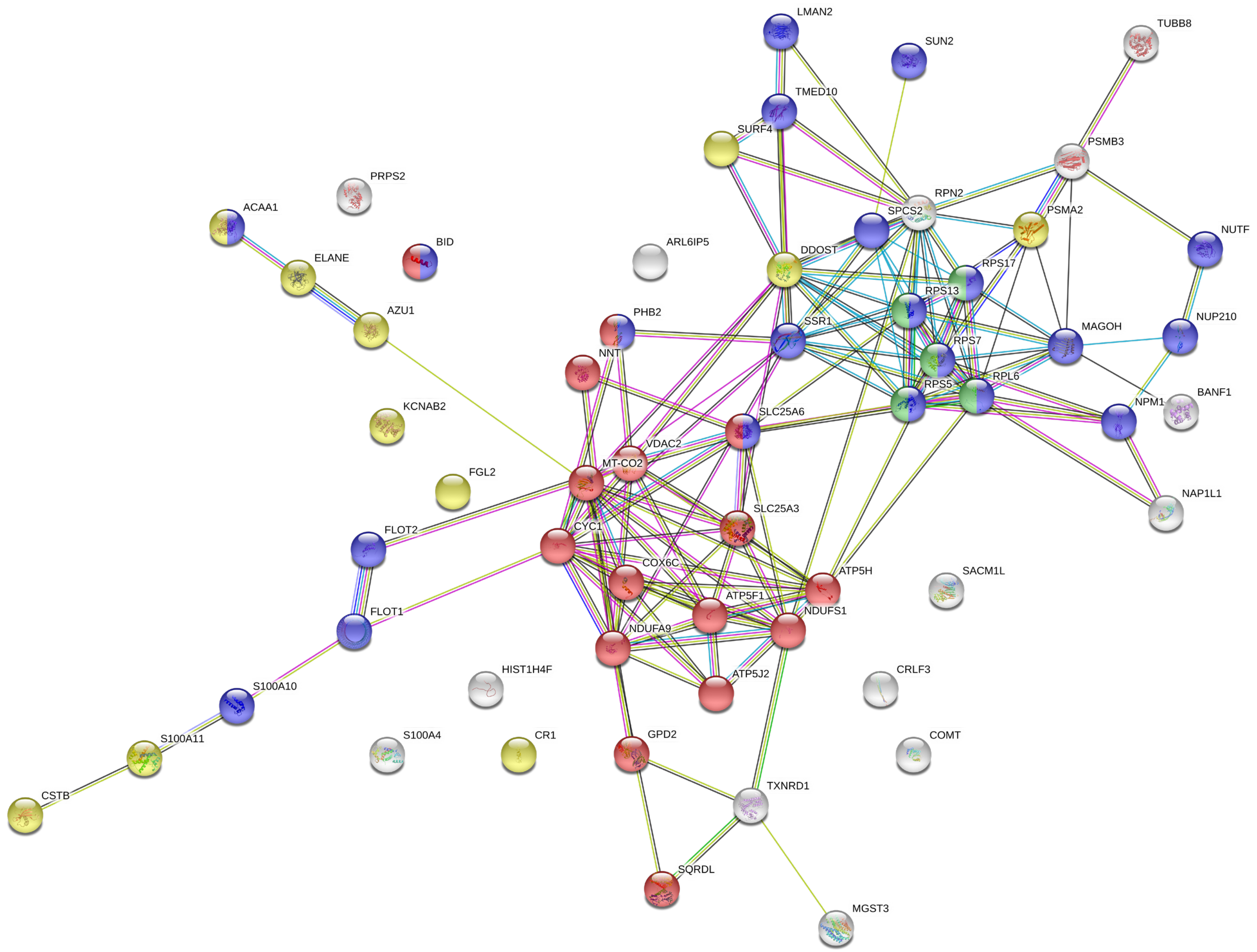

Figure S9. STRING derived protein-protein interaction network for proteins upregulated in the early adversity group at t1. Proteins colored in red are associated with mitochondrial biology, and proteins in blue are related to regulation of protein synthesis and transport. Yellow proteins relate to immune processes and neutrophil degranulation. Green proteins are implicated in viral transcription.

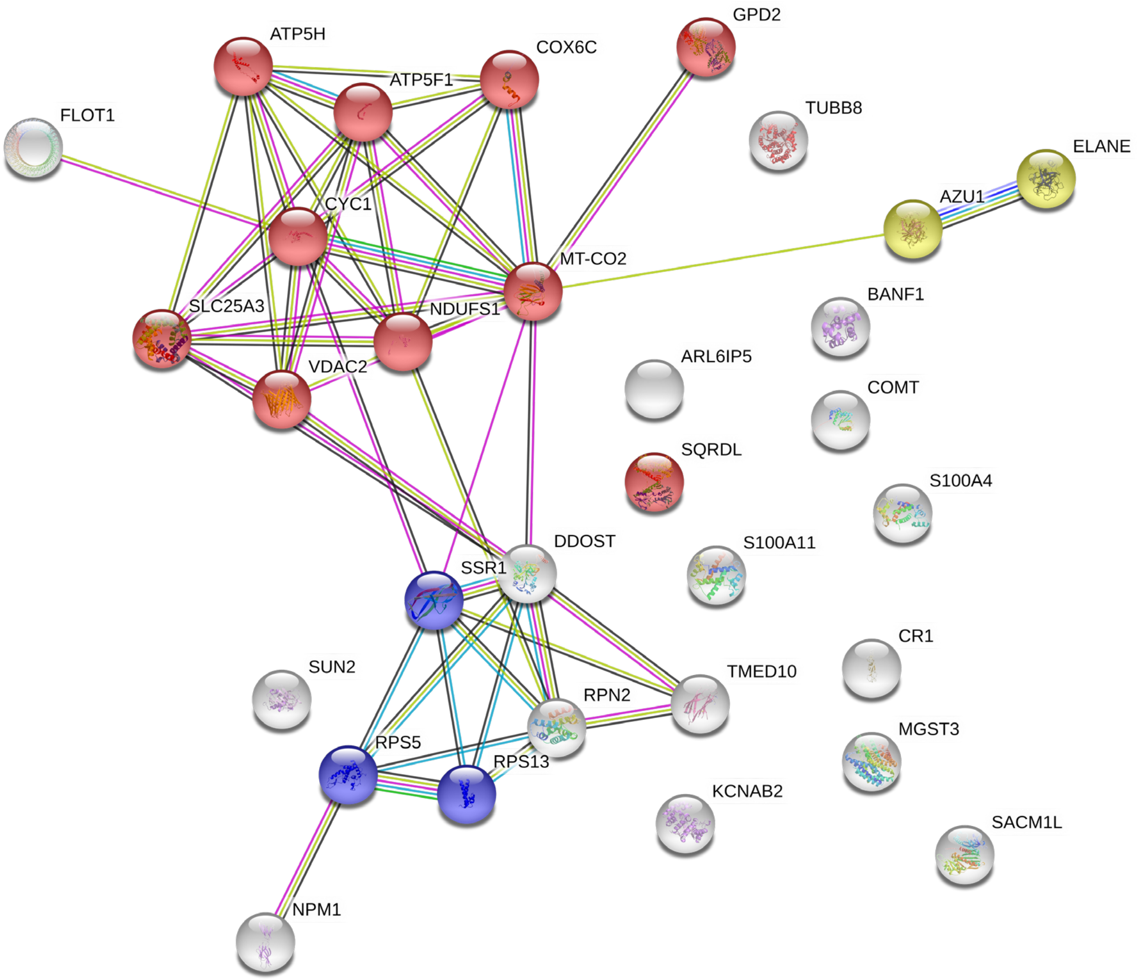

Figure S10. STRING derived protein-protein interaction network for proteins upregulated in the early adversity group at t0 and t1. Proteins colored in red are associated with mitochondrial biology, and proteins in blue are related to regulation of protein synthesis and transport. Yellow proteins relate to immune processes and neutrophil degranulation.

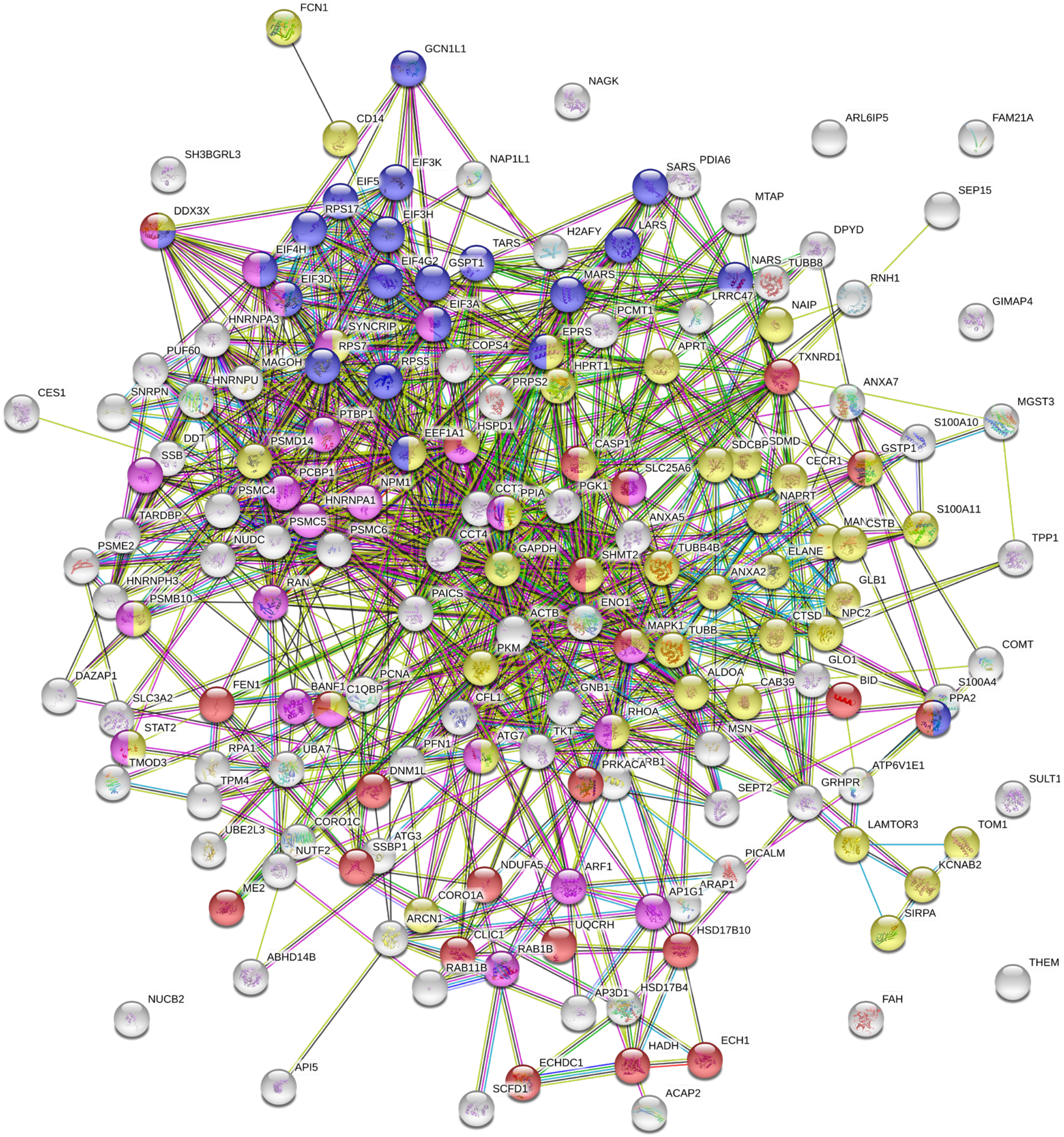

Figure S11. The salmon co-expression module (consensus baseline (t0) proteome). String derived protein-protein interaction networks. Protein colors indicate functional or topological associations within the salmon module (red: mitochondrion, blue: translation, yellow: immune response, pink: viral processes).

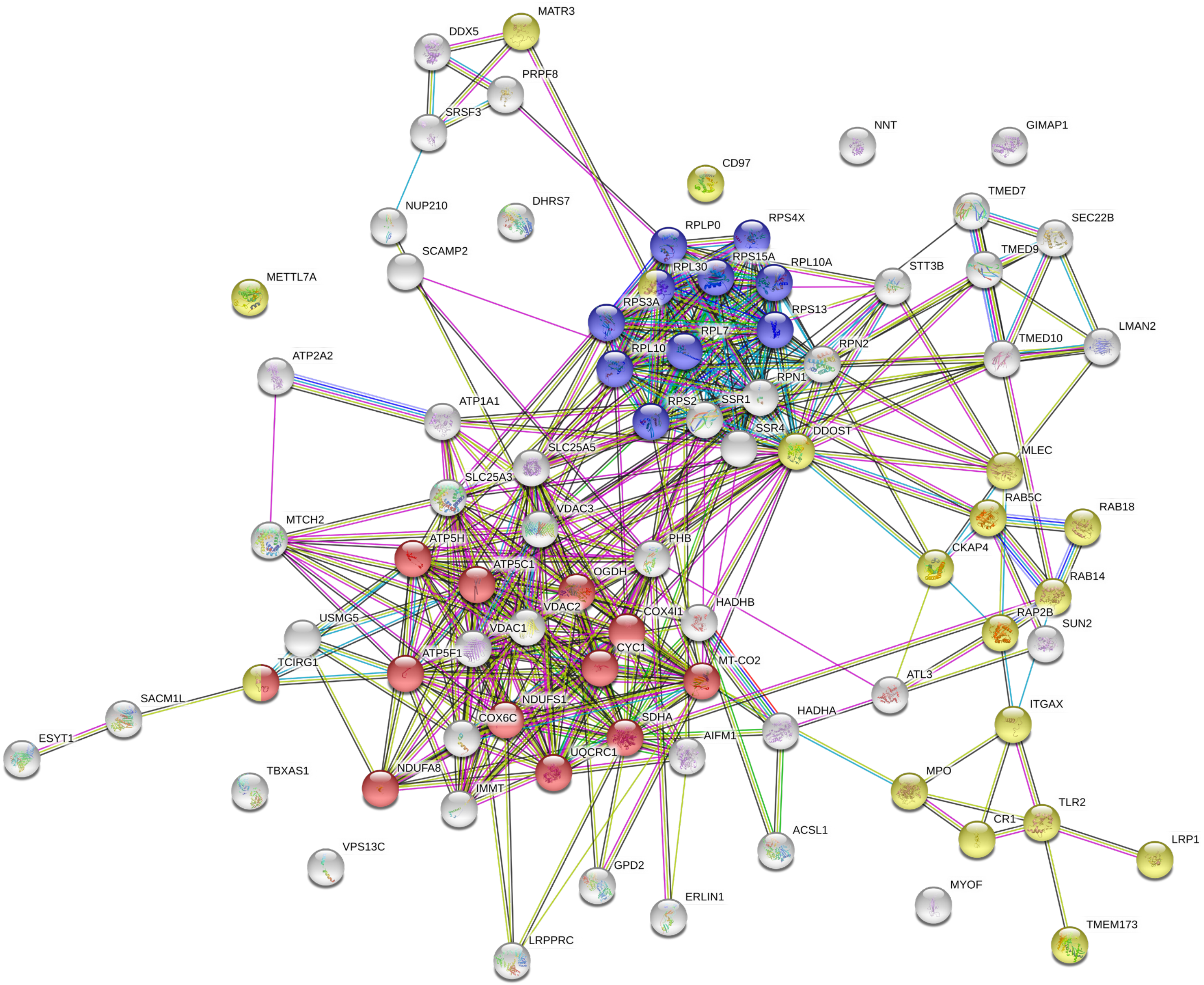

Figure S12. The purple co-expression module (consensus baseline (t0) proteome). Displayed is a String derived protein-protein interaction networks. Protein colours indicate functional or topological associations within the purple module (red: ATP metabolic processes, blue: translational initiation, yellow: immune response).

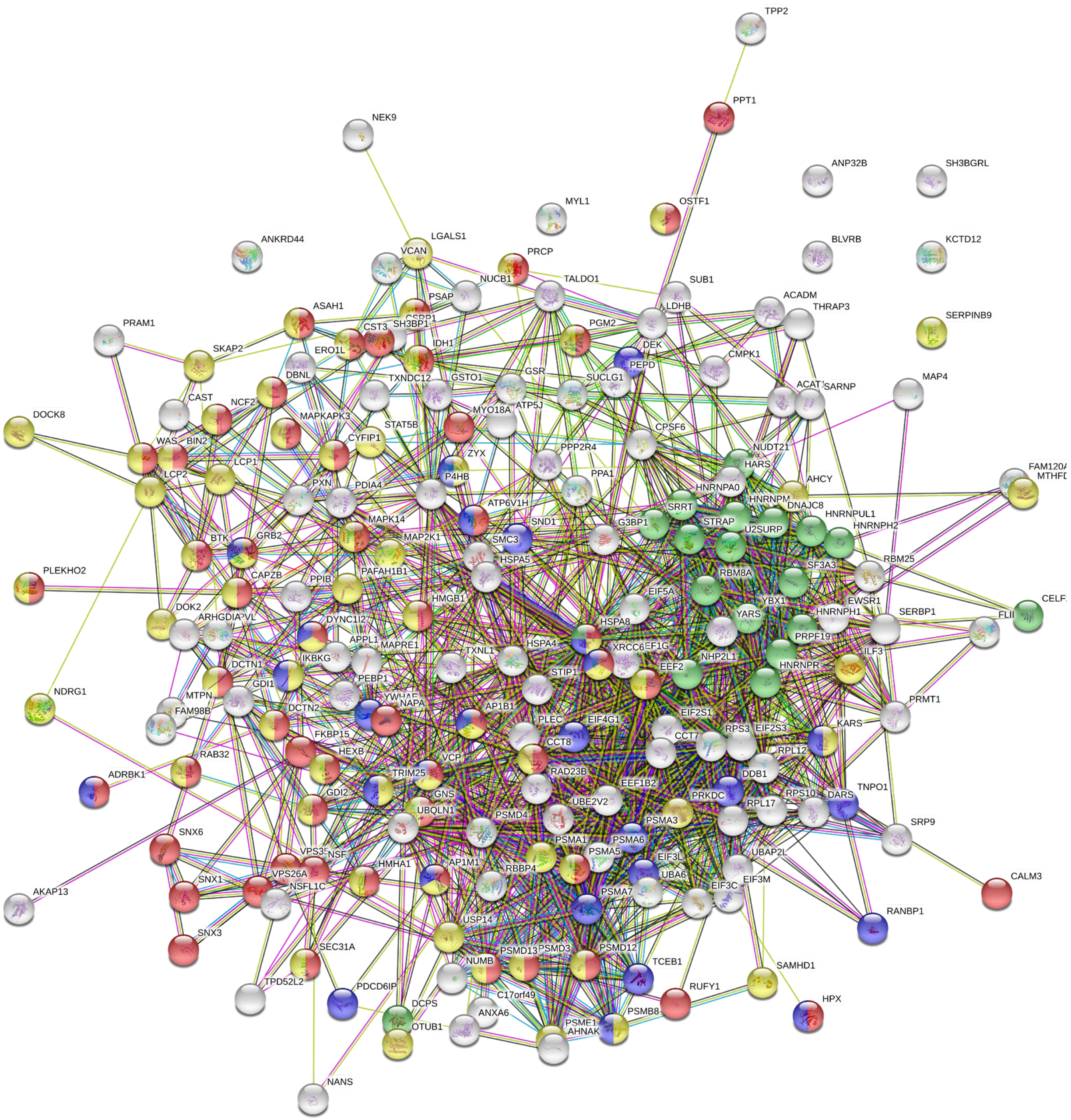

Figure S13. The light cyan co-expression module (consensus baseline (t0) proteome). Displayed is a String derived protein-protein interaction networks. Protein colours indicate functional or topological associations within the light cyan module (red: vesicle mediated transport, yellow: immune system processes, blue: viral processes, green: mRNA splicing).

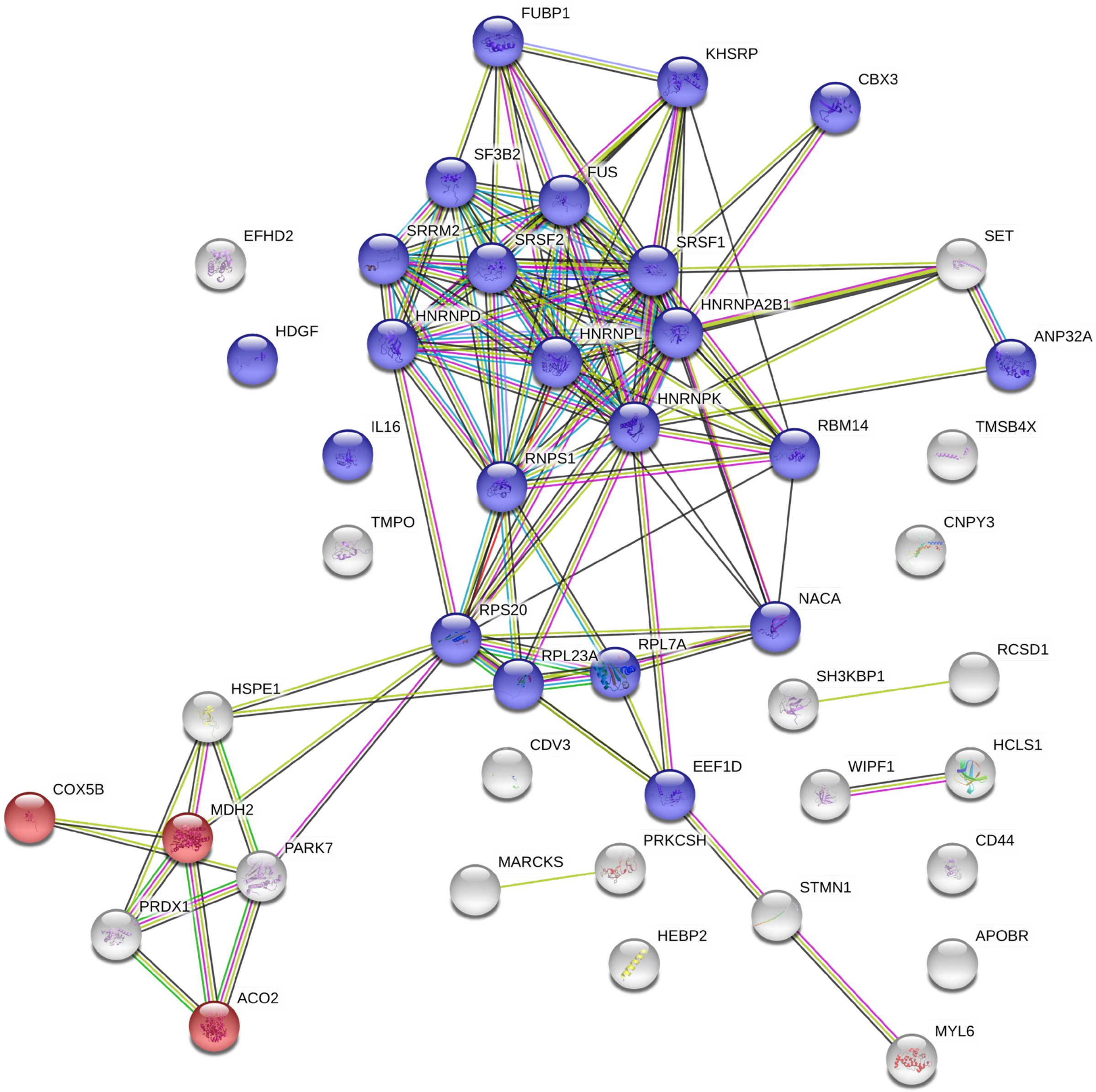

Figure S14. The midnight blue co-expression module (consensus baseline (t0) proteome). Displayed is a String derived protein-protein interaction networks. Protein colours indicate functional or topological associations within the midnight blue module (red: aerobic respiration, blue: gene expression).

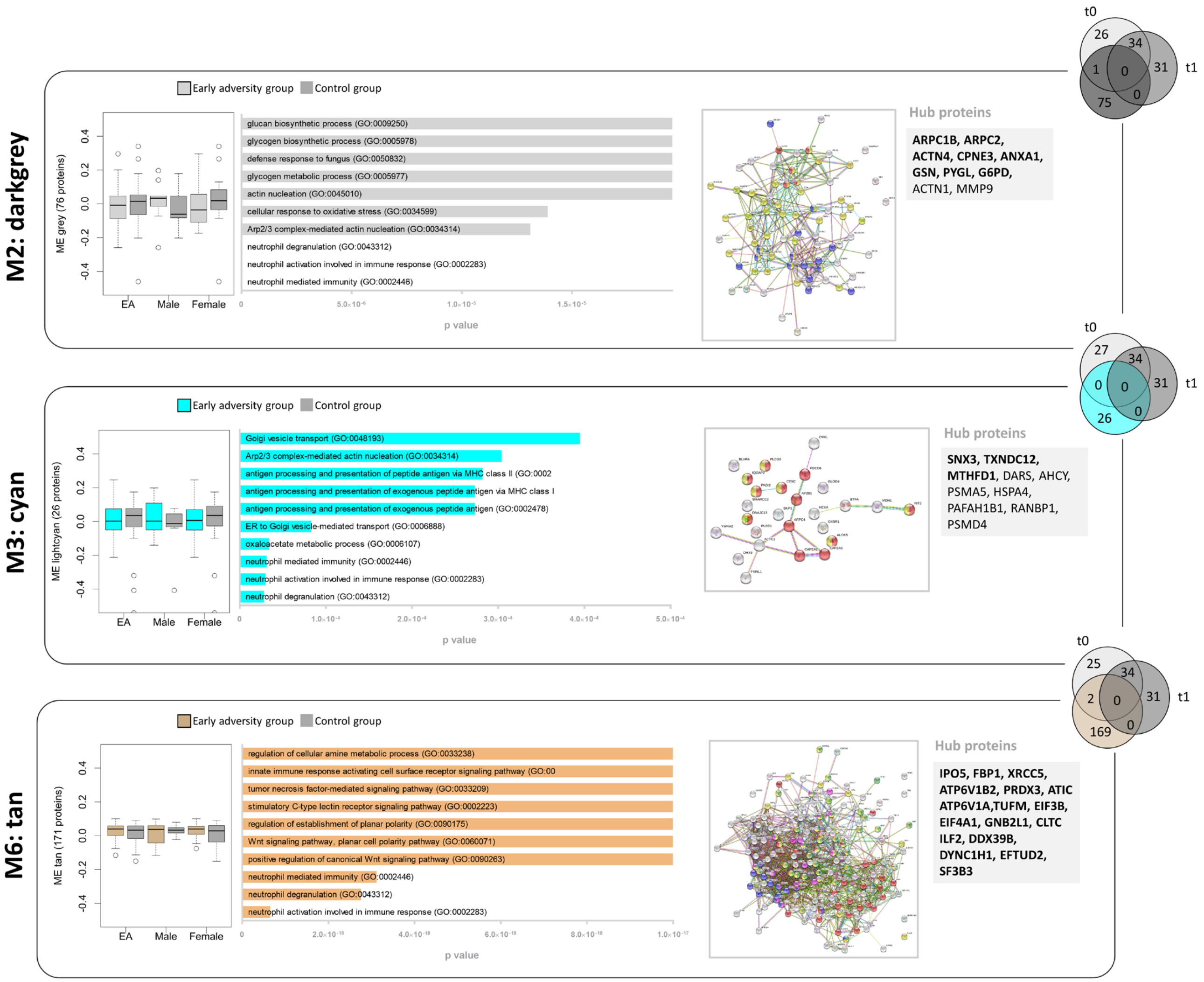

Figure S15. Further co-expression modules identified in the consensus baseline (t0) proteome.

Boxplots depict Module eigenprotein (ME) expression stratified by group (early adversity, control) and sex. Colored boxes represent values of participants with early adverse experiences. For each module, the ten most significantly enriched biological processes are reported and ranked by – log10 *p*-values). Venn Diagrams indicate the overlap of baseline module proteins and DEP_a identified at t0 and t1. String networks were generated for each of the presented modules. Protein colors indicate functional or topological associations within the cyan module (red: vesicle mediated transport, yellow: leukocyte activation involved in immune response), the dark grey module (yellow: leukocyte mediated immunity, blue: phagocytosis, red: ATP biosynthetic processes) and the tan module (green: exocytosis, yellow: immune response, pink: viral processes; blue: translation).

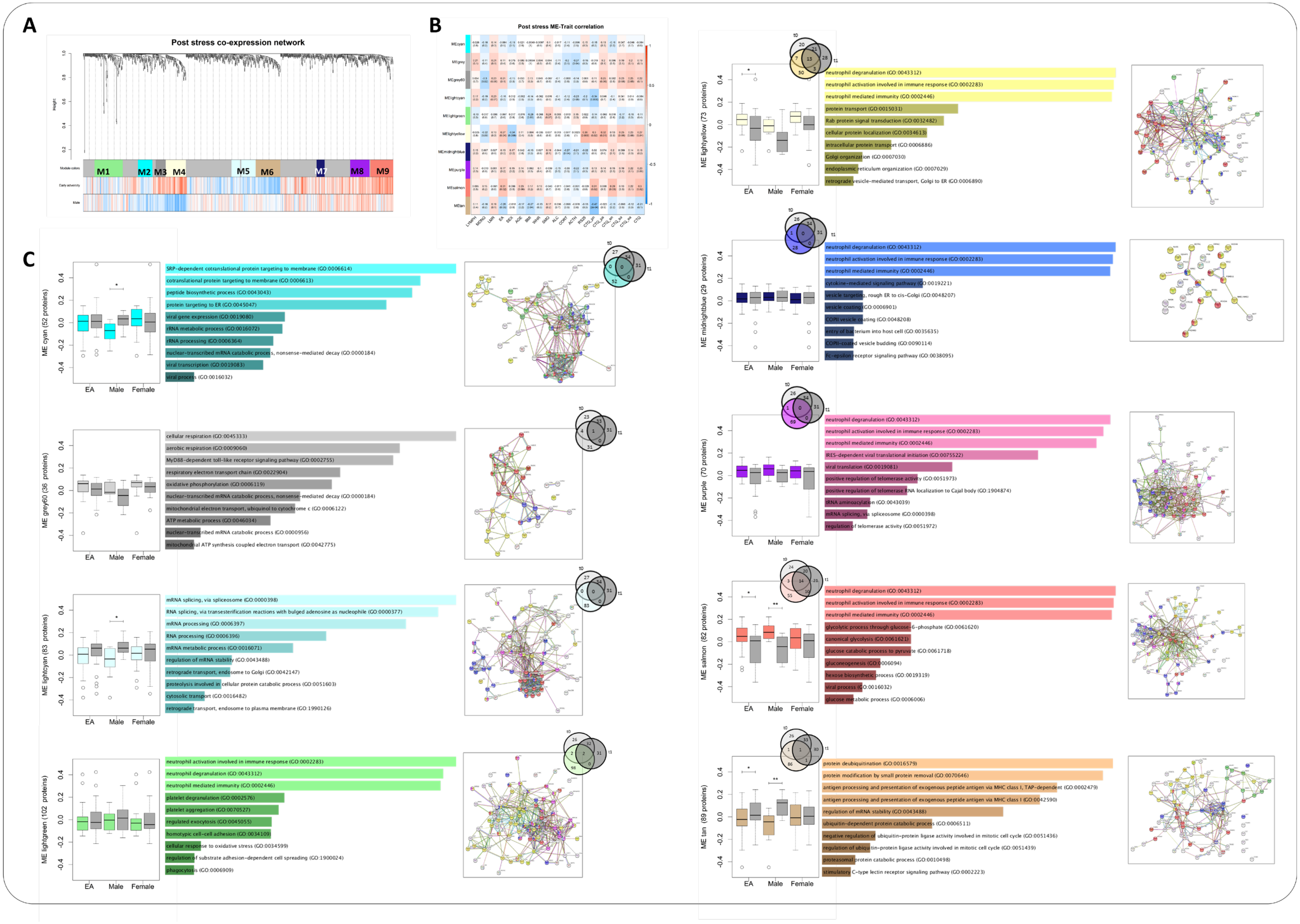
A
Post stress co-expression network
M1 M2 M3 M4 M5 M6 M7 M8 M9
B
Post stress ME-Trait correlation
C
SRP-dependent cotranslational protein targeting to membrane (GO:0006614)
cotranslational protein targeting to membrane (GO:0006613)
peptide biosynthetic process (GO:0043043)
protein targeting to ER (GO:0045047)
viral gene expression (GO:0019080)
rRNA metabolic process (GO:0016072)
rRNA processing (GO:0006364)
nuclear-transcribed mRNA catabolic process, nonsense-mediated decay (GO:0000184)
viral transcription (GO:0019083)
viral process (GO:0016032)
cellular respiration (GO:0045333)
aerobic respiration (GO:0009060)
MyD88-dependent toll-like receptor signaling pathway (GO:0002755)
respiratory electron transport chain (GO:0022904)
oxidative phosphorylation (GO:0006119)
nuclear-transcribed mRNA catabolic process, nonsense-mediated decay (GO:0000184)
mitochondrial electron transport, ubiquinol to cytochrome c (GO:0006122)
ATP metabolic process (GO:0046034)
nuclear-transcribed mRNA catabolic process (GO:0000956)
mitochondrial ATP synthesis coupled electron transport (GO:0042775)
mRNA splicing, via spliceosome (GO:0000398)
RNA splicing, via transesterification reactions with bulged adenosine as nucleophile (GO:0000377)
mRNA processing (GO:0006397)
RNA processing (GO:0006396)
mRNA metabolic process (GO:0016071)
regulation of mRNA stability (GO:0043488)
retrograde transport, endosome to Golgi (GO:0042147)
proteolysis involved in cellular protein catabolic process (GO:0051603)
cytosolic transport (GO:0016482)
retrograde transport, endosome to plasma membrane (GO:1990126)
neutrophil activation involved in immune response (GO:0002283)
neutrophil degranulation (GO:0043312)
neutrophil mediated immunity (GO:0002446)
platelet degranulation (GO:0002576)
platelet aggregation (GO:0070527)
regulated exocytosis (GO:0045055)
homotypic cell-cell adhesion (GO:0034109)
cellular response to oxidative stress (GO:0034599)
regulation of substrate adhesion-dependent cell spreading (GO:1900024)
phagocytosis (GO:0006909)
neutrophil degranulation (GO:0043312)
neutrophil activation involved in immune response (GO:0002283)
neutrophil mediated immunity (GO:0002446)
protein transport (GO:0015031)
Rab protein signal transduction (GO:0032482)
cellular protein localization (GO:0034613)
intracellular protein transport (GO:0006886)
Golgi organization (GO:0007030)
endoplasmic reticulum organization (GO:0007029)
retrograde vesicle-mediated transport, Golgi to ER (GO:0006890)
neutrophil degranulation (GO:0043312)
neutrophil activation involved in immune response (GO:0002283)
neutrophil mediated immunity (GO:0002446)
cytokine-mediated signaling pathway (GO:0019221)
vesicle targeting, rough ER to cis-Golgi (GO:0048207)
vesicle coating (GO:0006901)
COPII vesicle coating (GO:0048208)
entry of bacterium into host cell (GO:0035635)
COPII-coated vesicle budding (GO:0090114)
Fc-epsilon receptor signaling pathway (GO:0038095)
neutrophil degranulation (GO:0043312)
neutrophil activation involved in immune response (GO:0002283)
neutrophil mediated immunity (GO:0002446)
IRES-dependent viral translational initiation (GO:0075522)
viral translation (GO:0019081)
positive regulation of telomerase activity (GO:0051973)
positive regulation of telomerase RNA localization to Cajal body (GO:1904874)
tRNA aminoacylation (GO:0043039)
mRNA splicing, via spliceosome (GO:0000398)
regulation of telomerase activity (GO:0051972)
neutrophil degranulation (GO:0043312)
neutrophil activation involved in immune response (GO:0002283)
neutrophil mediated immunity (GO:0002446)
glycolytic process through glucose-6-phosphate (GO:0061620)
canonical glycolysis (GO:0061621)
glucose catabolic process to pyruvate (GO:0061718)
gluconeogenesis (GO:0006094)
hexose biosynthetic process (GO:0019319)
viral process (GO:0016032)
glucose metabolic process (GO:0006006)
protein deubiquitination (GO:0016579)
protein modification by small protein removal (GO:0070646)
antigen processing and presentation of exogenous peptide antigen via MHC class I, TAP-dependent (GO:0002479)
antigen processing and presentation of exogenous peptide antigen via MHC class I (GO:0042590)
regulation of mRNA stability (GO:0043488)
ubiquitin-dependent protein catabolic process (GO:0006511)
negative regulation of ubiquitin-protein ligase activity involved in mitotic cell cycle (GO:0051436)
regulation of ubiquitin-protein ligase activity involved in mitotic cell cycle (GO:0051439)
proteasomal protein catabolic process (GO:0010498)
stimulatory C-type lectin receptor signaling pathway (GO:0002223)
ME cyan (52 proteins)
ME grey60 (36 proteins)
ME lightcyan (83 proteins)
ME lightgreen (102 proteins)
ME lightyellow (73 proteins)
ME midnightblue (29 proteins)
ME purple (70 proteins)
ME salmon (82 proteins)
ME tan (89 proteins)
EA Male Female

Figure S16. Protein co-expression analysis in the post-stress (t1) proteome.
Dendrogram (A) of the consensus post-stress proteome comprising 1119 proteins. Each line represents a protein (leaf) and each low-hanging cluster represents a group of proteins with similar network connections (branch) on the tree. The first band underneath the tree indicates the nine detected modules and subsequent bands indicate protein-trait correlation. Red indicates a strong relationship and blue indicates a strong negative relationship. Module eigenproteins (ME) correlation with clinical variables are depicted (B), (EA=early aversive experience, CRQ= CTQ total score, RS25=RS25 total score, CORT &ACTH= base to peak ratio, BMI= Body mass index, WHR= waist to hip ratio, SEX= biological sex, AGE= age in years at participation, SMO= smoking, ALC= consumption of alcohol, LYMPH & MONO = count of lymphocytes and monocytes, LMR= lymphocyte to monocyte ratio. Linear models were used on ME values to test for significance between early adversity –control status across biological sex and * indicates p <0.05. Colored boxes represent values of participants with early aversive experiences. For each module, the ten most significant biological processes are reported and ranked by p-value. Venn Diagrams indicate the overlap of module proteins and DEP_a identified before (t0) and following (t1) stress exposure. String networks were generated for each of the presented modules. Protein colours indicate functional or topological associations within the salmon module (red: ATP metabolic processes, blue: protein transport, pink: viral processes, yellow: immune processes), the purple module (red: RNA splicing, blue: translational, pink: viral processes), the light cyan module (red: mRNA splicing via spliceosome, blue: protein transport, pink: viral processes, yellow: leukocyte activation),the midnight blue module (red: proteolysis, yellow: immune system processes, blue: entry of bacterium into host cell), the cyan module (red: translation initiation, blue: RNA processing, green: gene expression, yellow: leukocyte activation), the dark grey module (yellow: leukocyte mediated immunity, blue: translation initiation, green: cellular respiration, red: mitochondrial part), the tan module (red: regulation of cytoskeleton organization, blue: protein deubiquitination, yellow: leukocyte activation, green: mRNA processing),the light green module (yellow: leukocyte activation, pink: regulation of cytoskeleton organization, red: regulation of cell adhesion),the light yellow module (blue: secretion by cell, green: protein transport, yellow: immune response, red: mitochondrial part).

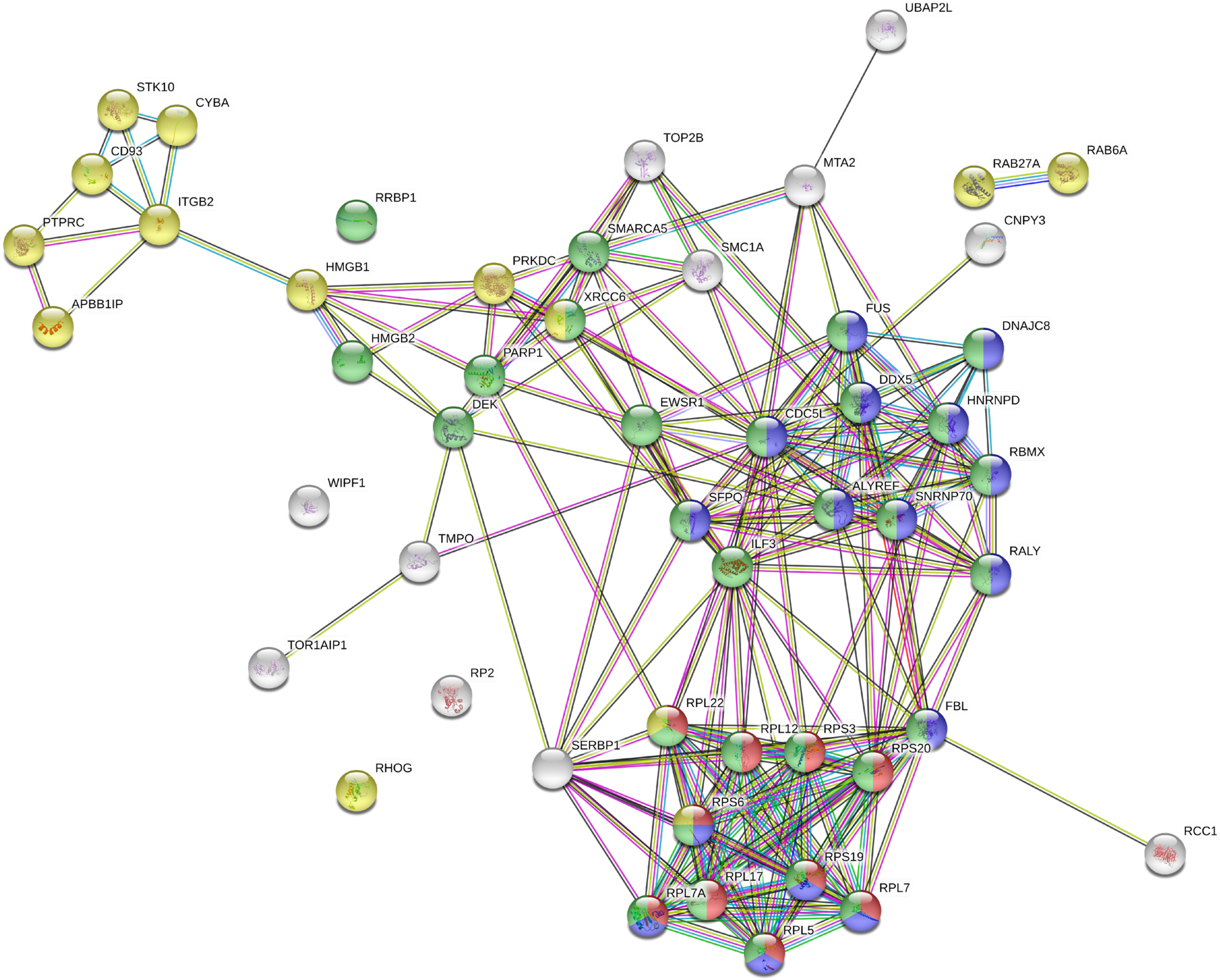

Figure S17. The cyan co-expression module (consensus post-stress (t1) proteome). Displayed is a String derived protein-protein interaction networks. Protein colors indicate functional or topological associations within the cyan module (red: translation initiation, blue: RNA processing, green: gene expression, yellow: leukocyte activation).

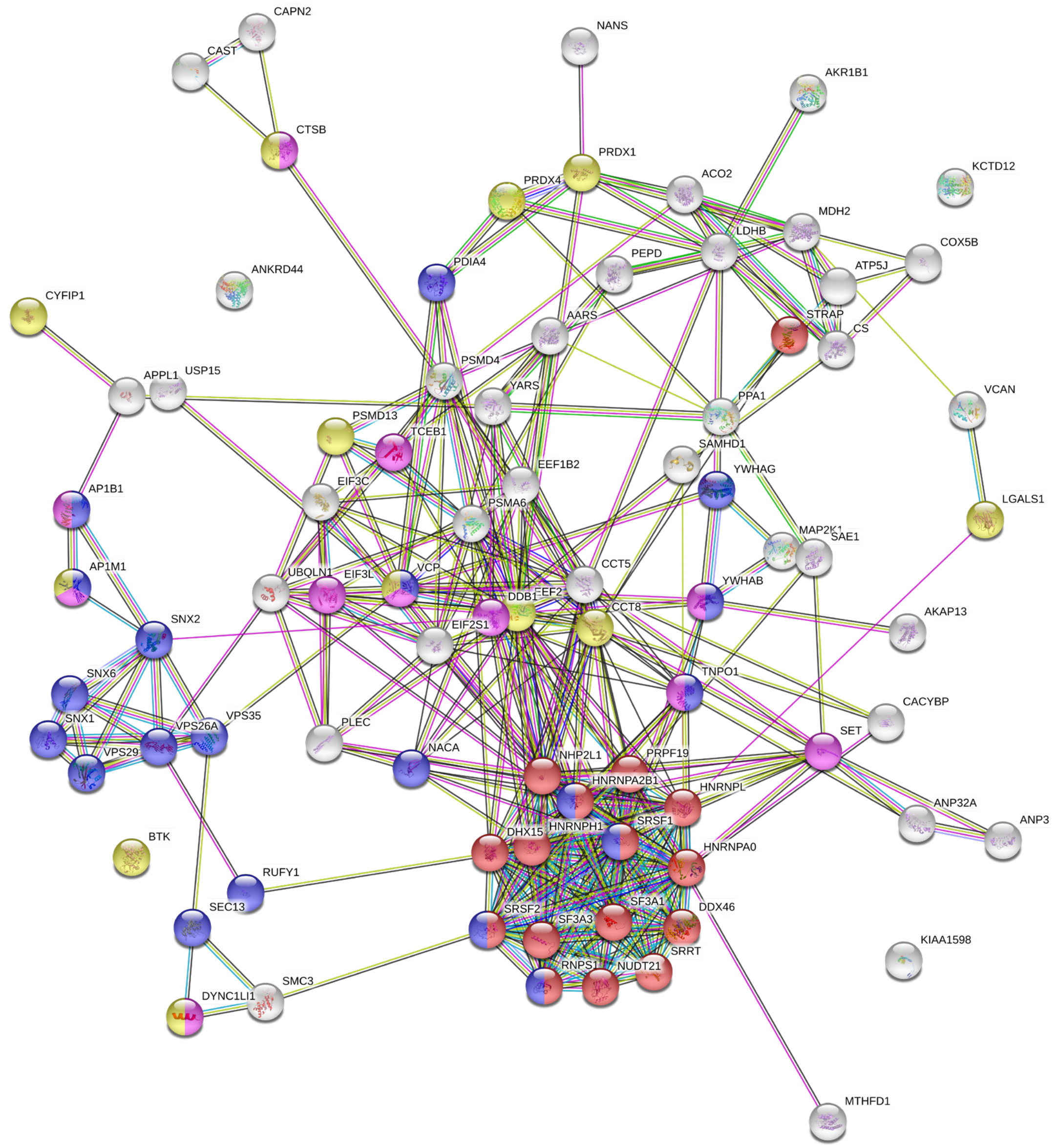

Figure S18. The light cyan co-expression module (consensus post-stress (t1) proteome). Displayed is a String derived protein-protein interaction networks. Protein colours indicate functional or topological associations within the light cyan module (red: mRNA splicing via spliceosome, blue: protein transport, pink: viral processes, yellow: leukocyte activation).

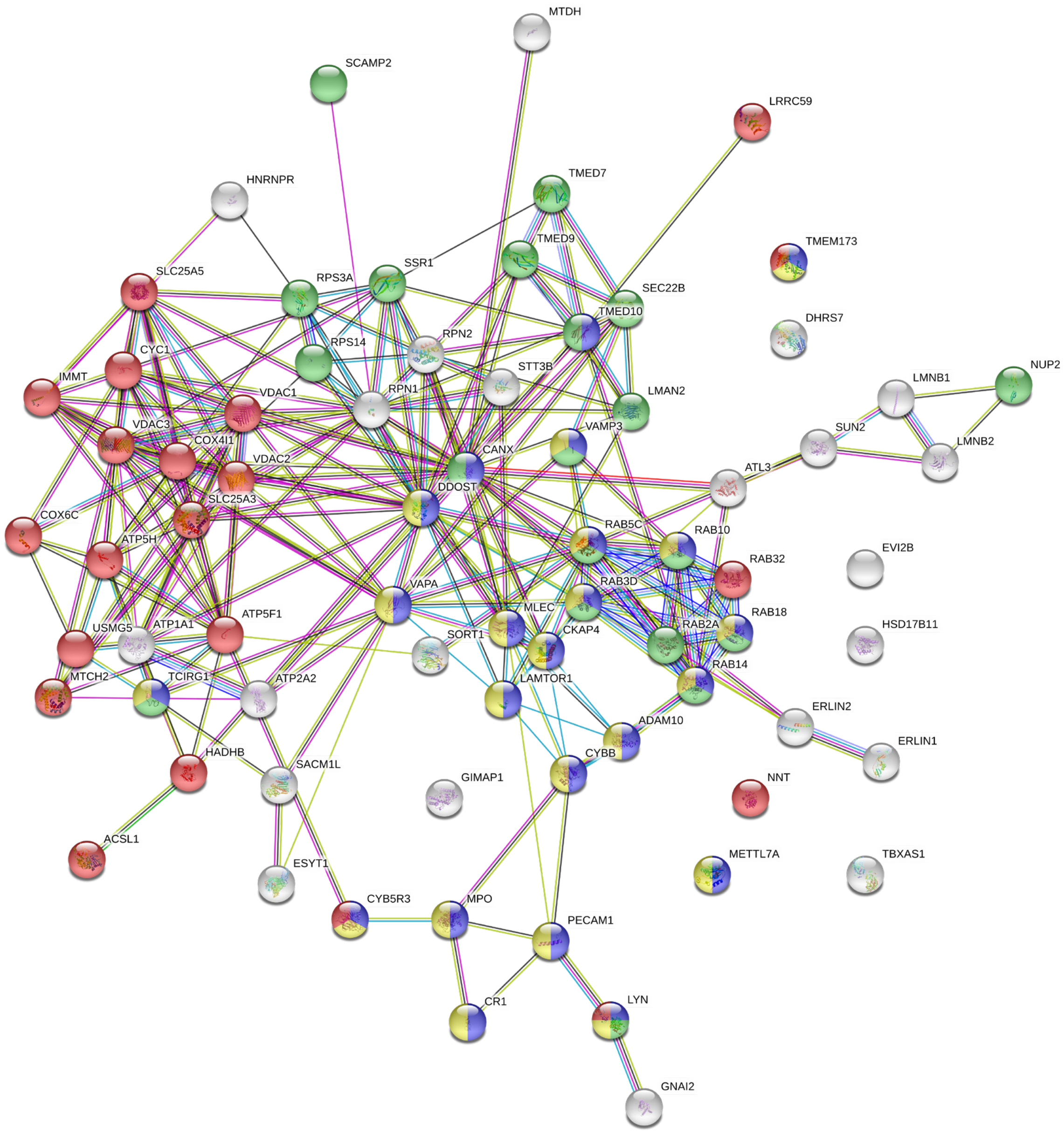

Figure S19. The light yellow co-expression module (consensus post-stress (t1) proteome). Displayed is a String derived protein-protein interaction networks. Protein colours indicate functional or topological associations within th light yellow module (blue: secretion by cell, green: protein transport, yellow: immune response, red: mitochondrial part).

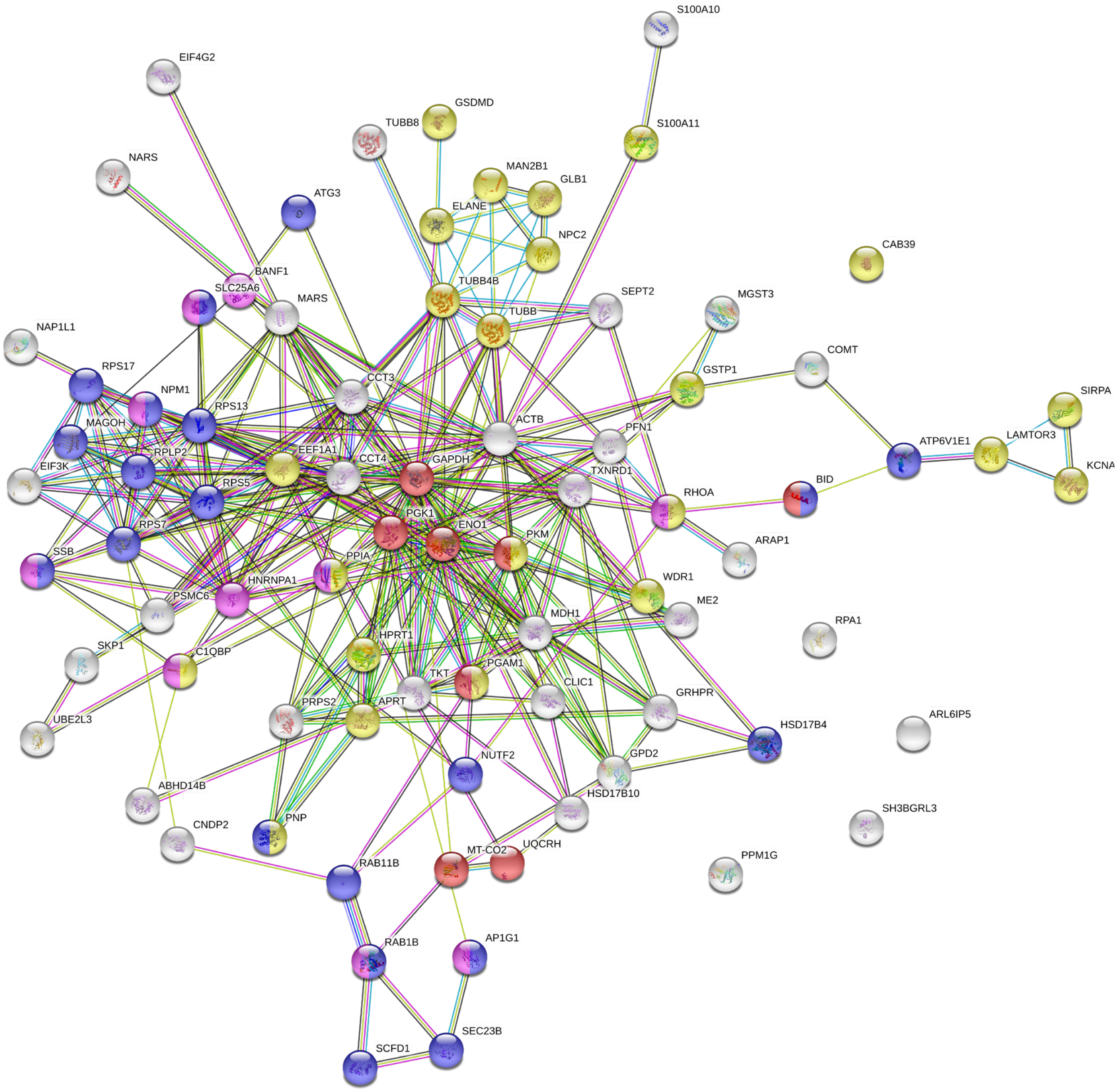

Figure S20. The salmon co-expression module (consensus post-stress (t1) proteome). Displayed is a String derived protein-protein interaction networks. Protein colors indicate functional or topological associations within the salmon module (red: ATP metabolic processes, blue: protein transport, pink: viral processes, yellow: immune processes).

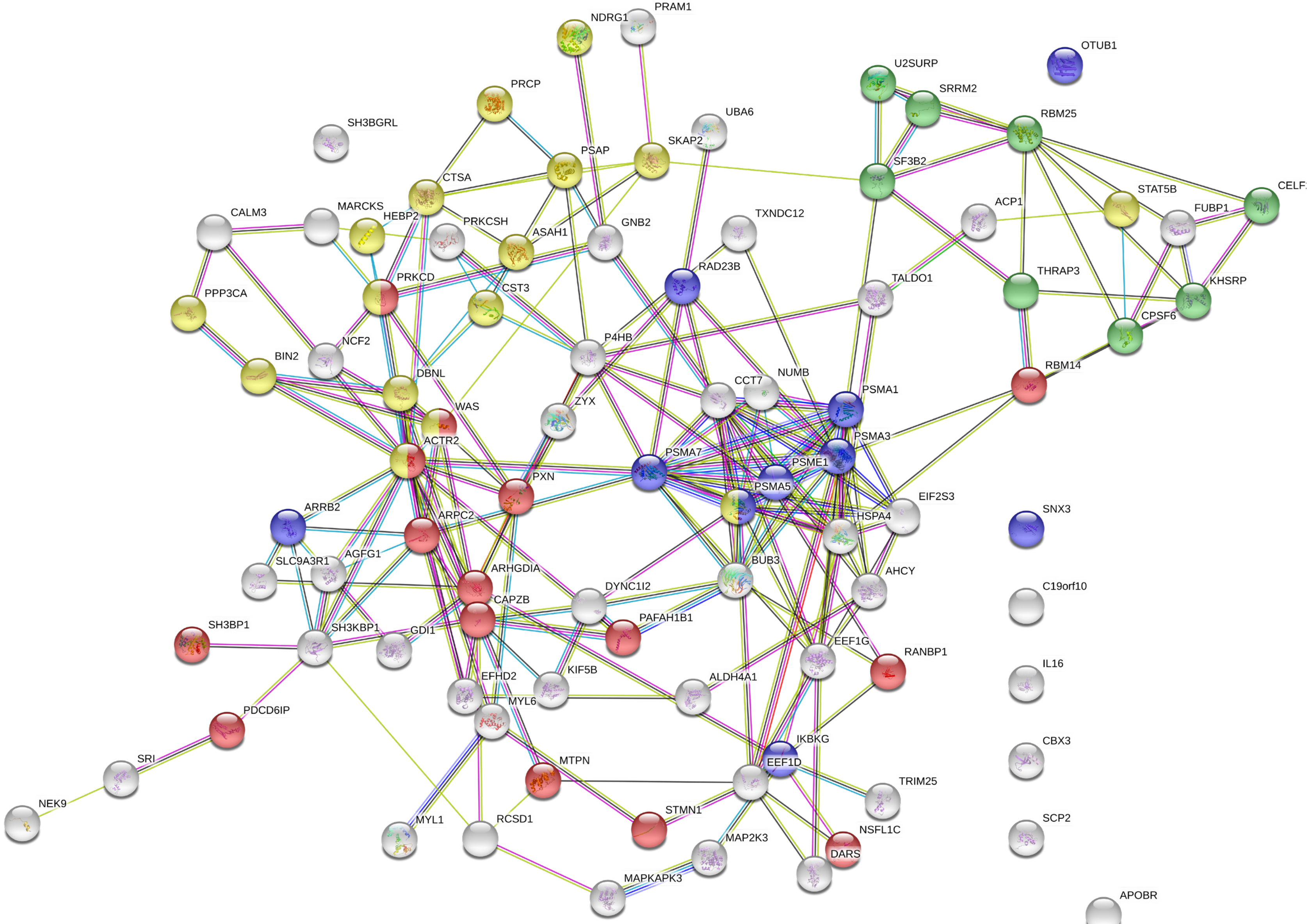

Figure S21. The tan co-expression module (consensus post-stress (t1) proteome). Displayed is a String derived protein-protein interaction networks. Protein colours indicate functional or topological associations within the tan module (red: regulation of cytoskeleton organization, blue: protein deubiquitination, yellow: leukocyte activation, green: mRNA processing).

Supplementary Tables

Table S1: Sample Characteristics. In the early adversity group, 76.6% of participants reported experience of three or more types of abuse and neglect. Table S1 shows the CTQ total scores as well as the number of participants that met cut-off criteria for moderate-serve exposure to the five CTQ categories. In the early adversity group, participants reported a higher number of previous mental disorders. Groups did not differ in resilience (RS-25), current psychopathological symptoms (BSI), baseline blood cell counts, their monocyte to lymphocyte ratio (MLR) or further social and bio-behavioral characteristics**.** Based on flow cytometric assessment, we detected no significant differences in purity of isolated CD14+ monocytes between groups. Income was assessed on a 1-4 scale (1 = up 1000€, 2= up to 2000€, 3= up to 3000€, 4= more than 3000€).

		**Early adversity group (n=30)**	**Control group (n = 29)**	***p***
Age	mean ± SD	52.57 ± 5.52	51.41 ± 4.81	0.40
Sex	number of females (%)	20 (66.7)	20 (68.9)	1
CTQ total score	mean ± SD	67.10 ± 16.04	35.34 ± 5.94	< .001
CTQ categories	n (%)			
Sexual abuse		15 (50.0)	-	
Physical abuse		15 (50.0)	-	
Emotional abuse		22 (73.3)	-	
Emotional neglect		26 (86.7)	-	
Physical neglect		21 (70.0)	-	
Sexual or physical abuse		23 (76.7)	-	
History of mental disorder	n (%)	18 (60)	7 (24.1)	0.01
Depression history	n (%)	12 (40)	3 (10.4)	0.02
BSI global severity index	mean ± SD	0.46 ± 0.41	0.33 ± 0.31	0.19
RS-25 total	mean ± SD	136.9 ± 26.7	137 ± 25.62	0.88
Childhood family income	mean ± SD*	1.67 ± 0.99	1.78 ± 0.8	0.65
Current family income	mean ± SD*	2.43 ± 1.1	2.52 ± 1.05	0.77
In a relationship	n (%)	11 (36.7)	11 (37.9)	0.97
No. of children		1.8 ± 1.24	1.37 ± 1.24	0.68
No. of persons in household		2.13 ± 1.31	1.93 ± 1.14	0.53
Body mass index	mean ± SD	25.29 ± 4.81	24.96 ± 3.66	0.77
Waist to hip ratio	mean ± SD	0.88 ± 0.1	0.85 ± 0.07	0.21
Smoking	*n* (%)	7 (23.3)	3 (10.4)	0.33
Baseline blood count				
Leukocytes	mean ± SD	6.82 (1.6)	6.64 (1.67)	0.17
Lymhocytes	mean ± SD	30.29 (7.6)	32.23 (7.0)	0.35
Monocytes	mean ± SD	6.08 (1.6)	6.61 (1.9)	0.26

		Early adversity group (n=30)	Control group (n = 29)	*p*
Neutrophils	mean ± SD	60.78 (7.8)	57.86 (7.8)	0.16
MLR	mean ± SD	5.33 ± 2.08	5.15 ± 1.47	0.72
Flow cytometric control				
T0 Monocyte isolation	mean ± SD purity %	93.92 (5.06)	91.8 (6.58)	0.17
T1 Monocyte isolation	mean ± SD purity %	93.02 (9.46)	93.38 (7.51)	0.91

Table S2: Baseline specific DEP$_a$. 27 proteins were baseline specific and differentially expressed between groups before stress exposure (t0). With an exception of MTPN and HP, all proteins showed significant higher abundances in participants with a history of childhood adversity. EA: early adversity. CG: control group. Q: evidence level indicating the percentage of proteins identified by valid value in at least one of the conditions compared. FC: observed fold changes in proteins expression, p: limma derived significance level.

Uniprot ID	Gene	Protein	Gene ontolotgy		Q	FC	p
			Biological process	Cellular component			
**Q9BSJ8**	**ESYT1**	Extended synaptotagmin-1	endoplasmic reticulum-plasma membrane tethering [GO:0061817]	endoplasmic reticulum [GO:0005783]	0.8	1.3	.022
**P08195**	**SLC3A2**	4F2 cell-surface antigen heavy chain	L-alpha-amino acid transmembrane transport [GO:1902475]	amino acid transport complex [GO:1990184]	0.8	1.3	.001
**P07437**	**TUBB**	Tubulin beta chain	G2/M transition of mitotic cell cycle [GO:0000086]	azurophil granule lumen [GO:0035578]	1.0	1.5	.010
**P61026**	**RAB10**	Ras-related protein Rab-10	Golgi to plasma membrane protein transport [GO:0043001]	Golgi apparatus [GO:0005794]	0.8	1.3	.012
**P53621**	**COPA**	Coatomer subunit alpha;Xenin;Proxenin	endoplasmic reticulum to Golgi vesicle-mediated transport [GO:0006888]	COPI vesicle coat [GO:0030126]	0.8	1.3	.034
**P55263**	**ADK**	Adenosine kinase	AMP salvage [GO:0044209]	cytosol [GO:0005829]	0.8	1.4	.049
**Q9NZ08**	**ERAP1**	Endoplasmic reticulum aminopeptidase 1	adaptive immune response [GO:0002250]	cytoplasm [GO:0005737]	0.8	1.4	.023
**P20674**	**COX5A**	Cytochrome c oxidase subunit 5A, mitochondrial	mitochondrial electron transport, cytochrome c to oxygen [GO:0006123]	mitochondrial inner membrane [GO:0005743]	0.8	1.4	.000
**P20701**	**ITGAL**	Integrin alpha-L	T cell activation via T cell receptor contact with antigen bound to MHC molecule on antigen presenting cell [GO:0002291]	cell surface [GO:0009986]	0.8	1.5	.027
**P62873**	**GNB1**	Guanine nucleotide-binding protein G(I)/G(S)/G(T) subunit beta-1	G protein-coupled acetylcholine receptor signaling pathway [GO:0007213]	cell body [GO:0044297]	0.8	1.4	.005
**P68036**	**UBE2L3**	Ubiquitin-conjugating enzyme E2 L3	cell cycle phase transition [GO:0044770]	cytoplasm [GO:0005737]	0.8	1.5	.036
**P0DMN0**	**SULT1A4;SULT1A3**	Sulfotransferase 1A4;Sulfotransferase 1A3	3'-phosphoadenosine 5'-phosphosulfate metabolic process [GO:0050427]	cytosol [GO:0005829]	0.8	1.4	.020
**Q3LXA3**	**DAK**	Bifunctional ATP-dependent dihydroxyacetone kinase/FAD-AMP lyase (cyclizing)	carbohydrate phosphorylation [GO:0046835]	cytosol [GO:0005829]	0.8	1.4	.012
**P42704**	**LRPPRC**	Leucine-rich PPR motif-containing protein, mitochondrial	mRNA transport [GO:0051028]	condensed nuclear chromosome [GO:0000794]	0.8	1.5	.011
**P21796**	**VDAC1**	Voltage-dependent anion-selective channel protein 1	anion transport [GO:0006820]	extracellular exosome [GO:0070062]	0.8	1.7	.004
**P20702**	**ITGAX**	Integrin alpha-X	animal organ morphogenesis [GO:0009887]	cell surface [GO:0009986]	0.8	1.4	.038
**Q6DD88**	**ATL3**	Atlastin-3	Golgi organization [GO:0007030]	endoplasmic reticulum [GO:0005783]	0.8	1.4	.024
**Q9Y6C9**	**MTCH2**	Mitochondrial carrier homolog 2	positive regulation of apoptotic process [GO:0043065]	integral component of membrane [GO:0016021]	0.8	1.4	.030
**P78324**	**SIRPA**	Tyrosine-protein phosphatase non-receptor type substrate 1	cell adhesion [GO:0007155]	cell surface [GO:0009986]	0.8	1.3	.011

Uniprot ID	Gene	Protein	Gene ontolotgy		Q	FC	p
			Biological process	Cellular component			
**P62701**	**RPS4X**	40S ribosomal protein S4, X isoform	SRP-dependent cotranslational protein targeting to membrane [GO:0006614]	cytoplasmic ribonucleoprotein granule [GO:0036464]	0.8	1.4	.003
**P04843**	**RPN1**	Dolichyl-diphosphooligosaccharide--protein glycosyltransferase subunit 1	cellular protein modification process [GO:0006464]	cytosol [GO:0005829]	0.8	1.3	.010
**Q93077**	**HIST1H2AC;HIST3H2A;HIST1H2AB**	Histone H2A type 1-C;Histone H2A type 3;Histone H2A type 1-B/E	chromatin organization [GO:0006325]	extracellular exosome [GO:0070062]	0.8	1.5	.023
**P33121**	**ACSL1**	Long-chain-fatty-acid--CoA ligase 1	adiponectin-activated signaling pathway [GO:0033211]	endoplasmic reticulum [GO:0005783]	0.8	1.5	.046
**P50225**	**SULT1A1**	Sulfotransferase 1A1	3'-phosphoadenosine 5'-phosphosulfate metabolic process [GO:0050427]	cytosol [GO:0005829]	1.0	1.5	.010
**Q16836**	**HADH**	Hydroxyacyl-coenzyme A dehydrogenase, mitochondrial	fatty acid beta-oxidation [GO:0006635]	cytoplasm [GO:0005737]	0.8	1.3	.015
**P00738**	**HP**	Haptoglobin	acute inflammatory response [GO:0002526)	blood microparticle [GO:0072562];	1.0	0.7	.040
**P58546**	**MTPN**	Myotrophin	catecholamine metabolic process [GO:0006584]	F-actin capping protein complex [GO:0008290)	1.0	0.8	.023

Table S3: Post-stress specific DEPa. 31 proteins were post-stress specific and differentially expressed between groups only at the post-sress measurement (t1). With an exception of MARCKS, ASM, IGHG3 and FKBP4, all proteins showed significant higher abundances in participants with a history of childhood adversity. EA: early adversity. CG: control group. Q: evidence level indicating the percentage of proteins identified by valid value in at least one of the conditions compared. FC: observed fold changes in proteins expression, p: limma derived significance level.

Uniprot ID	Gene	Protein	Gene ontolotgy		Q	FC	p
			Biological process	Cellular component			
Q14314	FGL2	Fibroleukin	T cell activation via T cell receptor contact with antigen bound to MHC molecule on antigen presenting cell [GO:0002291]	collagen-containing extracellular matrix [GO:0062023]	0.8	1.5	.001
P0CW22	RPS17L;RPS17	40S ribosomal protein S17-like;40S ribosomal protein S17	-	-	0.8	1.8	.002
Q16795	NDUFA9	NADH dehydrogenase [ubiquinone] 1 alpha subcomplex subunit 9, mitochondrial	circadian rhythm [GO:0007623]	mitochondrial matrix [GO:0005759]	0.8	1.3	.047
O15260	SURF4	Surfeit locus protein 4	Golgi organization [GO:0007030]	Golgi membrane [GO:0000139]	0.8	1.5	.028
P11908	PRPS2	Ribose-phosphate pyrophosphokinase 2	5-phosphoribose 1-diphosphate biosynthetic process [GO:0006015]	cytoplasm [GO:0005737]	0.8	1.3	.003
Q8IUI8	CRLF3	Cytokine receptor-like factor 3	G1/S transition of mitotic cell cycle [GO:0000082]	cytoplasm [GO:0005737]	0.8	1.4	.000
P49720	PSMB3	Proteasome subunit beta type-3	Fc-epsilon receptor signaling pathway [GO:0038095]	cytoplasm [GO:0005737]	0.8	1.4	.000
P55957	BID	BH3-interacting domain death agonist;BH3-interacting domain death agonist p15;BH3-interacting domain death agonist p13;BH3-interacting domain death agonist p11	activation of cysteine-type endopeptidase activity involved in apoptotic process [GO:0006919]	cytosol [GO:0005829]	0.8	1.6	.014
P01860	IGHG3	Ig gamma-3 chain C region	B cell receptor signaling pathway [GO:0050853]	blood microparticle [GO:0072562]	0.8	0.5	.003
P55209	NAP1L1	Nucleosome assembly protein 1-like 1	DNA replication [GO:0006260]	cytoplasm [GO:0005737]	0.8	1.4	.007
P12236	SLC25A6;SLC25A4	ADP/ATP translocase 3;ADP/ATP translocase 3, N-terminally processed;ADP/ATP translocase 1	apoptotic process [GO:0006915]	TIM23 mitochondrial import inner membrane translocase complex [GO:0005744]	0.8	1.4	.004
Q02878	RPL6	60S ribosomal protein L6	SRP-dependent cotranslational protein targeting to membrane [GO:0006614]	cytoplasmic ribonucleoprotein granule [GO:0036464]	0.8	1.5	.001
Q12907	LMAN2	Vesicular integral-membrane protein VIP36	Golgi organization [GO:0007030]	COPII-coated ER to Golgi transport vesicle [GO:0030134]	0.8	1.6	.001
P29966	MARCKS	Myristoylated alanine-rich C-kinase substrate	actin crosslink formation [GO:0051764]	actin cytoskeleton [GO:0015629]	0.8	0.7	.001
P61970	NUTF2	Nuclear transport factor 2	mRNA transport [GO:0051028]	cytosol [GO:0005829]	0.8	1.4	.016
P62081	RPS7	40S ribosomal protein S7	SRP-dependent cotranslational protein targeting to membrane [GO:0006614]	cytosol [GO:0005829]	0.8	1.4	.004
P09110	ACAA1	3-ketoacyl-CoA thiolase, peroxisomal	alpha-linolenic acid metabolic process [GO:0036109]	cytosol [GO:0005829]	0.8	1.7	.003
Q16881	TXNRD1	Thioredoxin reductase 1, cytoplasmic	cell population proliferation [GO:0008283]	cytoplasm [GO:0005737]	0.8	1.3	.005
Q13423	NNT	NAD(P) transhydrogenase, mitochondrial	NADPH regeneration [GO:0006740]	integral component of membrane [GO:0016021]	0.8	1.6	.026
P60903	S100A10	Protein S100-A10	membrane raft assembly [GO:0001765]	collagen-containing extracellular matrix [GO:0062023]	0.8	1.7	.017

Uniprot ID	Gene	Protein	Gene ontolotgy		Q	FC	p
			Biological process	Cellular component			
P01023	A2M	Alpha-2-macroglobulin	blood coagulation, intrinsic pathway [GO:0007597]	blood microparticle [GO:0072562]	0.8	0.6	.034
P61326	MAGOH;MAGOHB	Protein mago nashi homolog;Protein mago nashi homolog 2	RNA export from nucleus [GO:0006405]	catalytic step 2 spliceosome [GO:0071013]	0.8	1.4	.001
Q8TEM1	NUP210	Nuclear pore membrane glycoprotein 210	intracellular transport of virus [GO:0075733]	endoplasmic reticulum membrane [GO:0005789]	0.8	1.5	.017
Q14254	FLOT2	Flotillin-2	anterograde dendritic transport [GO:0098937]	acrosomal membrane [GO:0002080]	0.8	1.5	.001
Q15005	SPCS2	Signal peptidase complex subunit 2	protein targeting to ER [GO:0045047]	endoplasmic reticulum membrane [GO:0005789]	0.8	1.6	.014
P56134	ATP5J2	ATP synthase subunit f, mitochondrial	ATP biosynthetic process [GO:0006754]	integral component of membrane [GO:0016021]	0.8	1.7	.008
Q99623	PHB2	Prohibitin-2	cellular response to hypoxia [GO:0071456]	GABA-ergic synapse [GO:0098982]	0.8	1.4	.026
P25787	PSMA2	Proteasome subunit alpha type-2	Fc-epsilon receptor signaling pathway [GO:0038095]	P-body [GO:0000932]	0.8	1.6	.005
P62805	HIST1H4A	Histone H4	CENP-A containing nucleosome assembly [GO:0034080]	extracellular exosome [GO:0070062]	1.0	1.5	.022
P04080	CSTB	Cystatin-B	adult locomotory behavior [GO:0008344]	collagen-containing extracellular matrix [GO:0062023]	1.0	1.5	.000
Q02790	FKBP4	Peptidyl-prolyl cis-trans isomerase FKBP4;Peptidyl-prolyl cis-trans isomerase FKBP4, N-terminally processed	androgen receptor signaling pathway [GO:0030521];]	cytoplasm [GO:0005737]	0.8	0.8	.009

Table S4. Differentially expressed proteins after stress exposure (DEP_s). All DEP_s were up-regulated at the post-stress measurement (t1), with an exception of LTF. In both groups, DEP_s include proteins functionally related to adaptive immune processes and wound healing, e.g. FGA, FGB and FGG, whose up-regulation might represent a molecular resonate of the blood sampling procedure. EA: early adversity. CG: control group. Q: evidence level indicating the percentage of proteins identified by valid value in at least one of the conditions compared. FC: observed fold changes in proteins expression, p: limma derived significance level. EA = Early adversity group, CG = Control group

Group	Uniprot ID	Gene	Protein	Gene ontolotgy		Q	FC	p
				Biological process	Cellular component			
**EA**	**P02671**	**FGA**	Fibrinogen alpha chain	adaptive immune response [GO:0002250]; blood coagulation [GO:0007596]	cell surface [GO:0009986]; collagen-containing extracellular matrix [GO:0062023]; endoplasmic reticulum lumen [GO:0005788]	0.8	1.90	0.01
	**P02679**	**FGG**	Fibrinogen gamma chain	blood coagulation [GO:0007596], fibrin clot formation [GO:0072378]	cell surface [GO:0009986]; collagen-containing extracellular matrix [GO:0062023]; endoplasmic reticulum lumen [GO:0005788]	0.8	1.85	0.03
	**Q9Y376**	**CAB39**	Calcium-binding protein 39	cell cycle arrest [GO:0007050] intracellular signal transduction [GO:0035556]	cytosol [GO:0005829]; extracellular exosome [GO:0070062]	0.8	1.33	0.02
**CG**	**P02675**	**FGB**	Fibrinogen beta chain	adaptive immune response [GO:0002250]; blood coagulation [GO:0007596]	cell cortex [GO:0005938]; cell surface [GO:0009986]; collagen-containing extracellular matrix [GO:0062023]	0.8	2.05	0.047
	**P02671**	**FGA**	Fibrinogen alpha chain	adaptive immune response [GO:0002250]; blood coagulation [GO:0007596]	cell surface [GO:0009986]; collagen-containing extracellular matrix [GO:0062023]; endoplasmic reticulum lumen [GO:0005788]	0.8	2.04	0.008
	**P19367**	**HK1**	Hexokinase-1	cellular glucose homeostasis [GO:0001678]; establishment of protein localization to mitochondrion [GO:0072655	mitochondrial outer membrane [GO:0005741]; mitochondrion [GO:0005739]	0.8	1.39	0.001
	**Q13418**	**ILK**	Integrin-linked protein kinase	MAPK cascade [GO:0000165], cell-matrix adhesion [GO:0007160]	cytosol [GO:0005829],cell-cell junction [GO:0005911];	0.8	1.36	0.002
	**O00231**	**PSMD11**	26S proteasome non-ATPase regulatory subunit 11	MAPK cascade [GO:0000165]; NIK/NF-kappaB signaling [GO:0038061]	cytosol [GO:0005829]; extracellular region [GO:0005576]	0.8	1.30	0.022
	**P02788**	**LTF**	Lactotransferrin	antimicrobial humoral immune response mediated by antimicrobial peptide [GO:0061844]	cell surface [GO:0009986]; cytoplasm [GO:0005737]	0.8	0.51	0.023

Table S5: Adversity group: Cortisol response proteins. Spearman's correlation was calculated to relate participants cortisol response and stress-related FC in protein abundance. Q: evidence level indicating the percentage of proteins identified by valid value in at least one of the conditions compared in differential analysis (early adversity vs. control). r: Spearman's correlation coefficient, p: significance level.

Uniprot ID	Gene	Protein	Gene ontolotgy		Q	r	p
			GO: Biological process	GO:Cellular component			
**Q92974**	ARHGEF2	Rho guanine nucleotide exchange factor 2	G protein-coupled receptor signaling pathway [GO:0007186] actin filament organization [GO:0007015] asymmetric neuroblast division [GO:0055059]	Golgi apparatus [GO:0005794] bicellular tight junction [GO:0005923] cytoplasm [GO:0005737]	0.8	0.580	0.001
**Q709C8**	VPS13C	Vacuolar protein sorting-associated protein 13C	Golgi to endosome transport [GO:0006895] mitochondrion organization [GO:0007005] negative regulation of parkin-mediated stimulation of mitophagy in response to mitochondrial depolarization [GO:1905090]	cytoplasm [GO:0005737] cytosol [GO:0005829] extracellular exosome [GO:0070062]	0.8	0.465	0.010
**O60613**	SELENOF	15 kDa selenoprotein	'de novo' posttranslational protein folding [GO:0051084]	endoplasmic reticulum lumen [GO:0005788]	0.8	0.456	0.011
**O00231**	PSMD11	26S proteasome non-ATPase regulatory subunit 11	Fc-epsilon receptor signaling pathway [GO:0038095] MAPK cascade [GO:0000165] NIK/NF-kappaB signaling [GO:0038061]	cytosol [GO:0005829] extracellular region [GO:0005576] ficolin-1-rich granule lumen [GO:1904813]	0.8	0.455	0.012
**P23368**	ME2	NAD-dependent malic enzyme, mitochondrial	malate metabolic process [GO:0006108] pyruvate metabolic process [GO:0006090] regulation of NADP metabolic process [GO:1902031]	mitochondrial matrix [GO:0005759] mitochondrion [GO:0005739]	0.8	0.428	0.019
**Q92820**	GGH	Gamma-glutamyl hydrolase	neutrophil degranulation [GO:0043312] response to ethanol [GO:0045471] response to insulin [GO:0032868]	azurophil granule lumen [GO:0035578] cytosol [GO:0005829] extracellular exosome [GO:0070062]	0.8	0.425	0.020
**Q9NT62**	ATG3	Ubiquitin-like-conjugating enzyme ATG3	autophagosome assembly [GO:0000045] autophagy of mitochondrion [GO:0000422] cellular protein modification process [GO:0006464]	cytoplasmic ubiquitin ligase complex [GO:0000153] cytosol [GO:0005829]	0.8	0.414	0.024
**P60903**	S100A10	Protein S100-A10	membrane raft assembly [GO:0001765] positive regulation of GTPase activity [GO:0043547] positive regulation of binding [GO:0051099]	collagen-containing extracellular matrix [GO:0062023] endoplasmic reticulum [GO:0005783] extracellular exosome [GO:0070062]	0.8	0.399	0.030
**P14625**	HSP90B1	Endoplasmin	ATF6-mediated unfolded protein response [GO:0036500] actin rod assembly [GO:0031247] cellular protein metabolic process [GO:0044267]	collagen-containing extracellular matrix [GO:0062023] cytosol [GO:0005829] endocytic vesicle lumen [GO:0071682]	1	0.398	0.030
**Q9Y6N5**	SQRDL	Sulfide:quinone oxidoreductase, mitochondrial	hydrogen sulfide metabolic process [GO:0070813] sulfide oxidation, using sulfide:quinone oxidoreductase [GO:0070221]	mitochondrial inner membrane [GO:0005743]	0.8	0.390	0.034

Uniprot ID	Gene	Protein	Gene ontolotgy		Q	r	p
			GO: Biological process	GO:Cellular component			
**O60506**	SYNCRIP	Heterogeneous nuclear ribonucleoprotein Q	CRD-mediated mRNA stabilization [GO:0070934] RNA processing [GO:0006396] RNA splicing [GO:0008380]	CRD-mediated mRNA stability complex [GO:0070937] GAIT complex [GO:0097452] catalytic step 2 spliceosome [GO:0071013]	0.8	0.385	0.036
**P04083**	ANXA1	Annexin A1	DNA duplex unwinding [GO:0032508] G protein-coupled receptor signaling pathway [GO:0007186] G protein-coupled receptor signaling pathway, coupled to cyclic nucleotide second messenger [GO:0007187]	apical plasma membrane [GO:0016324] basolateral plasma membrane [GO:0016323] cell surface [GO:0009986]	1	0.369	0.045
**O00299**	CLIC1	Chloride intracellular channel protein 1	chloride transport [GO:0006821] platelet aggregation [GO:0070527] positive regulation of osteoblast differentiation [GO:0045669]	blood microparticle [GO:0072562] brush border [GO:0005903] chloride channel complex [GO:0034707]	1	0.366	0.047
**O14880**	MGST3	Microsomal glutathione S-transferase 3	glutathione derivative biosynthetic process [GO:1901687] leukotriene biosynthetic process [GO:0019370] lipid metabolic process [GO:0006629]	endoplasmic reticulum [GO:0005783] endoplasmic reticulum membrane [GO:0005789] integral component of membrane [GO:0016021]	0.8	0.365	0.047
**O75937**	DNAJC8	DnaJ homolog subfamily C member 8	mRNA splicing, via spliceosome [GO:0000398]	cytosol [GO:0005829] intercellular bridge [GO:0045171] nucleoplasm [GO:0005654]	0.8	-0.363	0.049
**P14317**	HCLS1	Hematopoietic lineage cell-specific protein	actin filament polymerization [GO:0030041] cellular response to cytokine stimulus [GO:0071345] erythrocyte differentiation [GO:0030218]	cortical actin cytoskeleton [GO:0030864] cytoplasm [GO:0005737] cytosol [GO:0005829]	1	-0.363	0.049
**O60763**	USO1	General vesicular transport factor p115	COPII vesicle coating [GO:0048208] Golgi organization [GO:0007030] Golgi vesicle docking [GO:0048211]	ER to Golgi transport vesicle membrane [GO:0012507] Golgi apparatus [GO:0005794] Golgi membrane [GO:0000139]	0.8	-0.365	0.048
**P35611**	ADD1	Alpha-adducin	IRE1-mediated unfolded protein response [GO:0036498] actin cytoskeleton organization [GO:0030036] actin filament bundle assembly [GO:0051017]	F-actin capping protein complex [GO:0008290] cell-cell adherens junction [GO:0005913] cytoplasm [GO:0005737]	0.8	-0.370	0.045
**Q13162**	PRDX4	Peroxiredoxin-4	4-hydroxyproline metabolic process [GO:0019471] I-kappaB phosphorylation [GO:0007252] cell redox homeostasis [GO:0045454]	cytosol [GO:0005829] endoplasmic reticulum [GO:0005783] extracellular exosome [GO:0070062]	0.8	-0.372	0.044
**Q13596**	SNX1	Sorting nexin-1	early endosome to Golgi transport [GO:0034498] intracellular protein transport [GO:0006886] lamellipodium morphogenesis [GO:0072673]	Golgi apparatus [GO:0005794] cytoplasm [GO:0005737] cytosol [GO:0005829]	0.8	-0.372	0.044

Uniprot ID	Gene	Protein	Gene ontolotgy		Q	r	p
			GO: Biological process	GO:Cellular component			
**O43865**	AHCYL1	Putative adenosylhomocysteinase 2	S-adenosylmethionine cycle [GO:0033353] angiotensin-activated signaling pathway [GO:0038166] epithelial fluid transport [GO:0042045]	apical plasma membrane [GO:0016324] cytoplasm [GO:0005737] cytosol [GO:0005829]	0.8	-0.373	0.043
**P84103**	SRSF3	Serine/arginine-rich splicing factor 3	RNA export from nucleus [GO:0006405] cellular response to leukemia inhibitory factor [GO:1990830] mRNA 3'-end processing [GO:0031124]	cytoplasm [GO:0005737] nuclear speck [GO:0016607] nucleoplasm [GO:0005654]	0.8	-0.377	0.041
**P30040**	ERP29	Endoplasmic reticulum resident protein 29	activation of MAPK activity [GO:0000187] intracellular protein transport [GO:0006886] negative regulation of gene expression [GO:0010629]	cell surface [GO:0009986] endoplasmic reticulum [GO:0005783] endoplasmic reticulum lumen [GO:0005788]	1	-0.379	0.040
**Q13185**	CBX3	Chromobox protein homolog 3	cellular response to DNA damage stimulus [GO:0006974] chromatin remodeling [GO:0006338] negative regulation of G0 to G1 transition [GO:0070317]	chromatin [GO:0000785] chromosome, centromeric region [GO:0000775] condensed chromosome, centromeric region [GO:0000779]	1	-0.385	0.037
**Q8TD55**	PLEKHO2	Pleckstrin homology domain-containing family O member 2	neutrophil degranulation [GO:0043312]	extracellular region [GO:0005576] ficolin-1-rich granule lumen [GO:1904813]	0.8	-0.385	0.037
**P61160**	ACTR2	Actin-related protein 2	Arp2/3 complex-mediated actin nucleation [GO:0034314] Fc-gamma receptor signaling pathway involved in phagocytosis [GO:0038096] associative learning [GO:0008306]	Arp2/3 protein complex [GO:0005885] actin cap [GO:0030478] actin cortical patch [GO:0030479]	1	-0.388	0.035
**Q12874**	SF3A3	Splicing factor 3A subunit 3	RNA splicing, via transesterification reactions [GO:0000375] U2-type prespliceosome assembly [GO:1903241] mRNA 3'-splice site recognition [GO:0000389]	U2 snRNP [GO:0005686] U2-type precatalytic spliceosome [GO:0071005] catalytic step 2 spliceosome [GO:0071013]	0.8	-0.388	0.035
**Q9UKY7**	CDV3	Protein CDV3 homolog	cell population proliferation [GO:0008283]	cytosol [GO:0005829] plasma membrane [GO:0005886]	1	-0.391	0.033
**P11586**	MTHFD1	C-1-tetrahydrofolate synthase, cytoplasmic;Methylenetetrahydrofolate dehydrogenase;	10-formyltetrahydrofolate biosynthetic process [GO:0009257] embryonic neurocranium morphogenesis [GO:0048702] embryonic viscerocranium morphogenesis [GO:0048703]	cytoplasm [GO:0005737] cytosol [GO:0005829] extracellular exosome [GO:0070062]	0.8	-0.404	0.028

Uniprot ID	Gene	Protein	Gene ontolotgy		Q	r	p
			GO: Biological process	GO:Cellular component			
**P36871**	PGM1	Phosphoglucomutase-1	galactose catabolic process [GO:0019388] gluconeogenesis [GO:0006094] glucose metabolic process [GO:0006006]	actin cytoskeleton [GO:0015629] cytoplasm [GO:0005737] cytosol [GO:0005829]	0.8	-0.406	0.027
**O95571**	ETHE1	Persulfide dioxygenase ETHE1, mitochondrial	glutathione metabolic process [GO:0006749] hydrogen sulfide metabolic process [GO:0070813] sulfide oxidation, using sulfide:quinone oxidoreductase [GO:0070221]	cytoplasm [GO:0005737] mitochondrial matrix [GO:0005759] mitochondrion [GO:0005739]	0.8	-0.407	0.026
**Q9H4A4**	RNPEP	Aminopeptidase B	negative regulation of blood pressure [GO:0045776] proteolysis [GO:0006508]	extracellular exosome [GO:0070062] extracellular region [GO:0005576] plasma membrane [GO:0005886]	1	-0.411	0.025
**Q14847**	LASP1	LIM and SH3 domain protein 1	ion transport [GO:0006811]	cortical actin cytoskeleton [GO:0030864] cytoplasm [GO:0005737] focal adhesion [GO:0005925]	0.8	-0.415	0.023
**P55795**	HNRNPH2	Heterogeneous nuclear ribonucleoprotein H2	RNA metabolic process [GO:0016070] mRNA splicing, via spliceosome [GO:0000398]	cytosol [GO:0005829] membrane [GO:0016020] nucleoplasm [GO:0005654]	0.8	-0.418	0.022
**O43684**	BUB3	Mitotic checkpoint protein BUB3	anaphase-promoting complex-dependent catabolic process [GO:0031145] attachment of spindle microtubules to kinetochore [GO:0008608] cell division [GO:0051301]	bub1-bub3 complex [GO:1990298] condensed chromosome kinetochore [GO:0000777] cytosol [GO:0005829]	0.8	-0.423	0.021
**P19878**	NCF2	Neutrophil cytosol factor 2	antigen processing and presentation of exogenous peptide antigen via MHC class I, TAP-dependent [GO:0002479] cell redox homeostasis [GO:0045454] cellular defense response [GO:0006968]	NADPH oxidase complex [GO:0043020] acrosomal vesicle [GO:0001669] cytosol [GO:0005829]	1	-0.430	0.018
**P28066**	PSMA5	Proteasome subunit alpha type-5	Fc-epsilon receptor signaling pathway [GO:0038095] MAPK cascade [GO:0000165] NIK/NF-kappaB signaling [GO:0038061]	cytoplasm [GO:0005737] cytosol [GO:0005829] extracellular exosome [GO:0070062]	1	-0.438	0.016
**P49757**	NUMB	Protein numb homolog	adherens junction organization [GO:0034332] axonogenesis [GO:0007409] lateral ventricle development [GO:0021670]	apical part of cell [GO:0045177] basolateral plasma membrane [GO:0016323] clathrin-coated vesicle [GO:0030136]	0.8	-0.438	0.016
**Q9Y2W1**	THRAP3	Thyroid hormone receptor-associated protein 3	RNA splicing [GO:0008380] circadian rhythm [GO:0007623] mRNA processing [GO:0006397]	extracellular exosome [GO:0070062] mediator complex [GO:0016592] nuclear speck [GO:0016607]	0.8	-0.438	0.016

Uniprot ID	Gene	Protein	Gene ontolotgy		Q	r	p
			GO: Biological process	GO:Cellular component			
**Q15233**	NONO	Non-POU domain-containing octamer-binding protein	DNA recombination [GO:0006310] DNA repair [GO:0006281] RNA splicing [GO:0008380]	RNA polymerase II transcription factor complex [GO:0090575] membrane [GO:0016020] nuclear matrix [GO:0016363]	1	-0.447	0.014
**P18859**	ATP5J	ATP synthase-coupling factor 6, mitochondrial	ATP biosynthetic process [GO:0006754] cristae formation [GO:0042407] mitochondrial ATP synthesis coupled proton transport [GO:0042776]	cell surface [GO:0009986] extracellular space [GO:0005615] mitochondrial inner membrane [GO:0005743]	0.8	-0.448	0.014
**O60234**	GMFG	Glia maturation factor gamma	actin filament debranching [GO:0071846] negative regulation of Arp2/3 complex-mediated actin nucleation [GO:0034316] neutrophil degranulation [GO:0043312]	actin cortical patch [GO:0030479] extracellular region [GO:0005576] ficolin-1-rich granule lumen [GO:1904813]	0.8	-0.452	0.013
**P62826**	RAN	GTP-binding nuclear protein Ran	DNA metabolic process [GO:0006259] GTP metabolic process [GO:0046039] androgen receptor signaling pathway [GO:0030521]	RNA nuclear export complex [GO:0042565] centriole [GO:0005814] cytoplasm [GO:0005737]	1	-0.455	0.012
**P62158**	CALM1	Calmodulin			1	-0.460	0.011
**Q9UNZ2**	NSFL1C	NSFL1 cofactor p47	Golgi organization [GO:0007030] autophagosome assembly [GO:0000045] establishment of mitotic spindle orientation [GO:0000132]	Golgi stack [GO:0005795] VCP-NSFL1C complex [GO:1990730] chromosome [GO:0005694]	0.8	-0.462	0.011
**P01889**	HLA-B	HLA class I histocompatibility antigen, B-7 alpha chain	antigen processing and presentation of exogenous peptide antigen via MHC class I, TAP-dependent [GO:0002479] antigen processing and presentation of exogenous peptide antigen via MHC class I, TAP-independent [GO:0002480] antigen processing and presentation of peptide antigen via MHC class I [GO:0002474]	ER to Golgi transport vesicle membrane [GO:0012507] Golgi apparatus [GO:0005794] Golgi membrane [GO:0000139]	0.8	-0.500	0.005
**Q8IXM2**	BAP18	Chromatin complexes subunit BAP18	chromatin organization [GO:0006325]	MLL1 complex [GO:0071339] NURF complex [GO:0016589] cytosol [GO:0005829]	0.8	-0.502	0.005
**Q99497**	PARK7	Protein deglycase DJ-1	DNA repair [GO:0006281] Ras protein signal transduction [GO:0007265] activation of protein kinase B activity [GO:0032148]	PML body [GO:0016605] axon [GO:0030424] cell body [GO:0044297]	1	-0.505	0.005
**P62942**	FKBP1A	Peptidyl-prolyl cis-trans isomerase FKBP1A	'de novo' protein folding [GO:0006458] SMAD protein complex assembly [GO:0007183] T cell activation [GO:0042110]	Z disc [GO:0030018] cytoplasm [GO:0005737] cytoplasmic side of membrane [GO:0098562]	1	-0.507	0.005
**P14866**	HNRNPL	Heterogeneous nuclear ribonucleoprotein L	RNA metabolic process [GO:0016070] RNA processing [GO:0006396] mRNA splicing, via spliceosome [GO:0000398]	cytoplasm [GO:0005737] extracellular exosome [GO:0070062] membrane [GO:0016020]	1	-0.512	0.004

Uniprot ID	Gene	Protein	Gene ontolotgy		Q	r	p
			GO: Biological process	GO:Cellular component			
**P53999**	SUB1	Activated RNA polymerase II transcriptional coactivator p15	SMAD protein signal transduction [GO:0060395] positive regulation of transcription initiation from RNA polymerase II promoter [GO:0060261] regulation of transcription by RNA polymerase II [GO:0006357]	extracellular exosome [GO:0070062] nucleolus [GO:0005730] nucleus [GO:0005634]	0.8	-0.519	0.004
**P33176**	KIF5B	Kinesin-1 heavy chain	anterograde axonal protein transport [GO:0099641] anterograde neuronal dense core vesicle transport [GO:1990048] cellular response to interferon-gamma [GO:0071346]	axon cytoplasm [GO:1904115] axonal growth cone [GO:0044295] ciliary rootlet [GO:0035253]	0.8	-0.549	0.002
**Q9UBR2**	CTSZ	Cathepsin Z	COPII vesicle coating [GO:0048208] angiotensin maturation [GO:0002003] endoplasmic reticulum to Golgi vesicle-mediated transport [GO:0006888]	COPII-coated ER to Golgi transport vesicle [GO:0030134] Golgi membrane [GO:0000139] cell cortex region [GO:0099738]	1	-0.674	0.000

Table S6: Control group: Cortisol response proteins. Spearman's correlation was calculated to relate participants' cortisol response and stress-related FC in protein abundance. Q: evidence level indicating the percentage of proteins identified by valid value in at least one of the conditions compared in differential analysis (early adversity vs. control). r: Spearman's correlation coefficient, p: significance level.

Uniprot ID	Gene	Protein	Gene ontolotgy		Q	r	p
			Biological process	Cellular component			
**Q12905**	**ILF2**	Interleukin enhancer-binding factor 2	Fc-gamma receptor signaling pathway involved in phagocytosis [GO:0038096] T cell meandering migration [GO:0120117] T cell mediated immunity [GO:0002456]	extracellular exosome [GO:0070062] filopodium [GO:0030175] lamellipodium [GO:0030027]	0.8	0.6369	0.0003
**P54920**	**NAPA**	Alpha-soluble NSF attachment protein	antigen processing and presentation of exogenous peptide antigen via MHC class I, TAP-dependent [GO:0002479] exocytosis [GO:0006887] histamine secretion by mast cell [GO:0002553]	SNARE complex [GO:0031201] azurophil granule [GO:0042582] cell-cell adherens junction [GO:0005913]	0.8	0.6310	0.0003
**O43776**	**NARS**	Asparagine--tRNA ligase, cytoplasmic	translation[GO:0006412] tRNA aminoacylation for protein translation[GO:0006418] asparaginyl-tRNA aminoacylation[GO:0006421]	cytoplasm[GO:0005737] cytosol[GO:0005829] extracellular exosome[GO:0070062]	0.8	0.6300	0.0003
**63208**	**SKP1**	S-phase kinase-associated protein 1	IRES-dependent viral translational initiation [GO:0075522] formation of cytoplasmic translation initiation complex [GO:0001732] protein deubiquitination [GO:0016579]	cytosol [GO:0005829] eukaryotic 43S preinitiation complex [GO:0016282] eukaryotic 48S preinitiation complex [GO:0033290]	0.8	0.6271	0.0004
**Q08945**	**SSRP1**	FACT complex subunit SSRP1	NLS-bearing protein import into nucleus [GO:0006607] cellular response to amino acid stimulus [GO:0071230] negative regulation of cyclin-dependent protein serine/threonine kinase activity [GO:0045736]	cytoplasm [GO:0005737] membrane [GO:0016020] nuclear pore [GO:0005643]	0.8	0.6252	0.0003
**Q8WVM8**	**SCFD1**	Sec1 family domain-containing protein 1	COPII vesicle coating [GO:0048208] G1/S transition of mitotic cell cycle [GO:0000082] innate immune response [GO:0045087]	Golgi membrane [GO:0000139] cytosol [GO:0005829] mitochondrion [GO:0005739]	0.8	0.6161	0.0004

**O75643**	**SNRNP200**	U5 small nuclear ribonucleoprotein 200 kDa helicase	RNA metabolic process [GO:0016070] circadian rhythm [GO:0007623] mRNA destabilization [GO:0061157]	axon terminus [GO:0043679] catalytic step 2 spliceosome [GO:0071013] dendrite [GO:0030425]	0.8	0.60540.0006
**B0I1T2**	**MYO1G**	Unconventional myosin-Ig;Minor histocompatibility antigen HA-2	regulation of cell population proliferation [GO:0042127]	cytoplasm [GO:0005737] perinuclear region of cytoplasm [GO:0048471]	0.8	0.60200.0007
**P35754**	**GLRX**	Glutaredoxin-1	asparaginyl-tRNA aminoacylation [GO:0006421] tRNA aminoacylation for protein translation [GO:0006418]	cytoplasm [GO:0005737] cytosol [GO:0005829] extracellular exosome [GO:0070062]	0.8	0.59950.0007
**Q13045**	**FLII**	Protein flightless-1 homolog	CRD-mediated mRNA stabilization [GO:0070934] RNA processing [GO:0006396] RNA splicing [GO:0008380]	CRD-mediated mRNA stability complex [GO:0070937] GAIT complex [GO:0097452] catalytic step 2 spliceosome [GO:0071013]	0.8	0.59560.0008
**Q9NQR4**	**NIT2**	Omega-amidase NIT2	chromatin organization [GO:0006325] dosage compensation [GO:0007549] establishment of protein localization to chromatin [GO:0071169]	Barr body [GO:0001740] condensed chromosome [GO:0000793] extracellular exosome [GO:0070062]	0.8	0.58470.0011
**P50897**	**PPT1**	Palmitoyl-protein thioesterase 1	carbohydrate metabolic process [GO:0005975] citrate metabolic process [GO:0006101] tricarboxylic acid cycle [GO:0006099]	extracellular exosome [GO:0070062] mitochondrial matrix [GO:0005759] mitochondrion [GO:0005739]	0.8	0.58130.0012
**Q7L1Q6**	**BZW1**	Basic leucine zipper and W2 domain-containing protein 1	Wnt signaling pathway [GO:0016055] intracellular protein transport [GO:0006886] regulation of macroautophagy [GO:0016241]	cytosol [GO:0005829] early endosome [GO:0005769] endosome [GO:0005768]	0.8	0.57990.0010
**P09769**	**FGR**	Tyrosine-protein kinase Fgr	cis assembly of pre-catalytic spliceosome [GO:0000354] mRNA splicing, via spliceosome [GO:0000398] osteoblast differentiation [GO:0001649]	U2-type catalytic step 1 spliceosome [GO:0071006] U2-type precatalytic spliceosome [GO:0071005] U4/U6 x U5 tri-snRNP complex [GO:0046540]	0.8	0.57680.0013

**O95782**	**AP2A1**	AP-2 complex subunit alpha-1	2-oxoglutarate metabolic process [GO:0006103] NADP metabolic process [GO:0006739] NADPH regeneration [GO:0006740]	cytoplasm [GO:0005737] cytosol [GO:0005829] extracellular exosome [GO:0070062]	0.8	0.57340.0014
**Q9UFN0**	**NIPSNAP3A**	Protein NipSnap homolog 3A	actin filament severing [GO:0051014] cortical actin cytoskeleton organization [GO:0030866] regulation of cell shape [GO:0008360]	bleb [GO:0032059] cell cortex [GO:0005938] cytosol [GO:0005829]	0.8	0.56950.0015
**P48735**	**IDH2**	Isocitrate dehydrogenase [NADP], mitochondrial	activation of protein kinase activity [GO:0032147] cellular hypotonic response [GO:0071476] cellular response to chemokine [GO:1990869]	cytoplasm [GO:0005737] cytosol [GO:0005829] extracellular exosome [GO:0070062]	1	0.56550.0017
**P07384**	**CAPN1**	Calpain-1 catalytic subunit	Golgi to endosome transport [GO:0006895] Wnt signaling pathway, planar cell polarity pathway [GO:0060071] antigen processing and presentation of exogenous peptide antigen via MHC class II [GO:0019886]	AP-2 adaptor complex [GO:0030122] apical plasma membrane [GO:0016324] basolateral plasma membrane [GO:0016323]	1	0.56450.0017
**O75390**	**CS**	Citrate synthase, mitochondrial	NAD metabolic process [GO:0019674] glycolytic process [GO:0006096] lactate metabolic process [GO:0006089]	cytosol [GO:0005829] extracellular exosome [GO:0070062] membrane [GO:0016020]	0.8	0.56060.0019
**Q92608**	**DOCK2**	Dedicator of cytokinesis protein 2	membrane raft polarization[GO:0001766] establishment of T cell polarity[GO:0001768] immunological synapse formation [GO:0001771]	extracellular region[GO:0005576] cytoplasm[GO:0005737] cytosol[GO:0005829]	0.8	0.55520.0021
**P25098**	**ADRBK1**	Beta-adrenergic receptor kinase 1	immune response [GO:0006955] inosine catabolic process [GO:0006148] interleukin-2 secretion [GO:0070970]	cytoplasm [GO:0005737] cytoskeleton [GO:0005856] cytosol [GO:0005829]	0.8	0.55420.0021

**P22695**	**UQCRC2**	Cytochrome b-c1 complex subunit 2, mitochondrial	T cell receptor signaling pathway [GO:0050852] adaptive immune response [GO:0002250] antigen processing and presentation of exogenous peptide antigen via MHC class II [GO:0019886]	ER to Golgi transport vesicle membrane [GO:0012507] Golgi membrane [GO:0000139] MHC class II protein complex [GO:0042613]	0.8	0.55070.0023
**Q53EL6**	**PDCD4**	Programmed cell death protein 4	cellular protein modification process [GO:0006464] protein N-linked glycosylation via asparagine [GO:0018279]	cytosol [GO:0005829] endoplasmic reticulum [GO:0005783] endoplasmic reticulum membrane [GO:0005789]	0.8	0.54680.0025
**O00303**	**EIF3F**	Eukaryotic translation initiation factor 3 subunit F	alcohol metabolic process [GO:0006066] carbohydrate metabolic process [GO:0005975] ethanol catabolic process [GO:0006068]	extracellular exosome [GO:0070062] mitochondrial matrix [GO:0005759]	0.8	0.54190.0024
**Q9HC38**	**GLOD4**	Glyoxalase domain-containing protein 4	fibrinolysis [GO:0042730] interleukin-12-mediated signaling pathway [GO:0035722] negative regulation of apoptotic process [GO:0043066]	cytoplasm [GO:0005737] extracellular region [GO:0005576] extracellular space [GO:0005615]	0.8	0.54140.0028
**Q92841**	**DDX17**	Probable ATP-dependent RNA helicase DDX17	SRP-dependent cotranslational protein targeting to membrane [GO:0006614] nuclear-transcribed mRNA catabolic process, nonsense-mediated decay [GO:0000184] translation [GO:0006412]	cytosol [GO:0005829] cytosolic large ribosomal subunit [GO:0022625] extracellular exosome [GO:0070062]	1	0.53500.0032
**P53004**	**BLVRA**	Biliverdin reductase A	heme catabolic process[GO:0042167] oxidation-reduction process[GO:0055114]	cytoplasm[GO:0005737] cytosol[GO:0005829] extracellular exosome[GO:0070062]	0.8	0.53450.0032
**P54136**	**RARS**	Arginine--tRNA ligase, cytoplasmic	G1/S transition of mitotic cell cycle [GO:0000082] RNA export from nucleus [GO:0006405] behavioral fear response [GO:0001662]	P-body [GO:0000932] RISC complex [GO:0016442] chromatoid body [GO:0033391]	0.8	0.53400.0033

**P27635**	**RPL10**	60S ribosomal protein L10	5-phosphoribose 1-diphosphate biosynthetic process [GO:0006015] glucose homeostasis [GO:0042593] glycogen catabolic process [GO:0005980]	cytoplasm [GO:0005737] cytosol [GO:0005829] extracellular exosome [GO:0070062]	0.8	0.52860.0036
**Q14152**	**EIF3A**	Eukaryotic translation initiation factor 3 subunit A	carbohydrate metabolic process [GO:0005975] chondroitin sulfate catabolic process [GO:0030207] glycosaminoglycan biosynthetic process [GO:0006024]	azurophil granule [GO:0042582] extracellular exosome [GO:0070062] lysosomal lumen [GO:0043202]	0.8	0.52660.0038
**O95747**	**OXSR1**	Serine/threonine-protein kinase OSR1	UV protection [GO:0009650] angiogenesis involved in wound healing [GO:0060055] blood vessel endothelial cell migration [GO:0043534]	cytoplasm [GO:0005737] cytosol [GO:0005829] mitochondrial matrix [GO:0005759]	0.8	0.52080.0038
**P26368**	**U2AF2**	Splicing factor U2AF 65 kDa subunit	nucleic acid binding[GO:0003676] RNA binding[GO:0003723] protein binding [GO:0005515]	commitment complex[GO:0000243] colocalizes_with Prp19 complex[GO:0000974] nucleus[GO:0005634]	0.8	0.51970.0043
**P15880**	**RPS2**	40S ribosomal protein S2	cornification [GO:0070268] extracellular matrix disassembly [GO:0022617] mammary gland involution [GO:0060056]	cytoplasm [GO:0005737] cytosol [GO:0005829] extracellular exosome [GO:0070062]	0.8	0.51720.0046
**Q15631**	**TSN**	Translin	B cell homeostasis [GO:0001782] B cell receptor signaling pathway [GO:0050853] Fc receptor mediated inhibitory signaling pathway [GO:0002774]	Golgi apparatus [GO:0005794] cell-cell adherens junction [GO:0005913] cytoplasm [GO:0005737]	0.8	0.51360.0044
**Q92974**	**ARHGEF2**	Rho guanine nucleotide exchange factor 2	Fc-gamma receptor signaling pathway involved in phagocytosis [GO:0038096] cell adhesion [GO:0007155] cell differentiation [GO:0030154]	Golgi apparatus [GO:0005794] caveola [GO:0005901] cell projection [GO:0042995]	0.8	0.51160.0046
**P60228**	**EIF3E**	Eukaryotic translation initiation factor 3 subunit E	Fc-gamma receptor signaling pathway involved in phagocytosis [GO:0038096] bone mineralization [GO:0030282] cell differentiation [GO:0030154]	actin cytoskeleton [GO:0015629] aggresome [GO:0016235] cytosol [GO:0005829]	0.8	0.50840.0054

**O75367**	**H2AFY**	Core histone macro-H2A.1	negative regulation of transcription by RNA polymerase II[GO:0000122] chromatin organization[GO:0006325] nucleosome assembly [GO:0006334]	nuclear chromosome [GO:0000228] nuclear chromosome, telomeric region[GO:0000784] chromatin[GO:0000785}	0.8	0.49900.0064
**P30044**	**PRDX5**	Peroxiredoxin-5, mitochondrial	5-phosphoribose 1-diphosphate biosynthetic process [GO:0006015] nucleobase-containing compound metabolic process [GO:0006139] nucleoside metabolic process [GO:0009116]	cytoplasm [GO:0005737] ribose phosphate diphosphokinase complex [GO:0002189]	0.8	0.49900.0064
**P17858**	**PFKL**	ATP-dependent 6-phosphofructokinase, liver type	actin filament polymerization [GO:0030041] cellular response to cytokine stimulus [GO:0071345] erythrocyte differentiation [GO:0030218]	cortical actin cytoskeleton [GO:0030864] cytoplasm [GO:0005737] cytosol [GO:0005829]	0.8	0.49800.0066
**P05388;Q8NHW5**	**RPLP0;RPLP0P6**	60S acidic ribosomal protein P0;60S acidic ribosomal protein P0-like	antigen processing and presentation of exogenous peptide antigen via MHC class I, TAP-dependent [GO:0002479] apoptotic process [GO:0006915] cell redox homeostasis [GO:0045454]	Golgi apparatus [GO:0005794] NADPH oxidase complex [GO:0043020] cytosol [GO:0005829]	0.8	0.49750.0066
**Q08211**	**DHX9**	ATP-dependent RNA helicase A	SRP-dependent cotranslational protein targeting to membrane [GO:0006614] nuclear-transcribed mRNA catabolic process, nonsense-mediated decay [GO:0000184] positive regulation of ubiquitin-protein transferase activity [GO:0051443]	cytosol [GO:0005829] cytosolic small ribosomal subunit [GO:0022627] extracellular exosome [GO:0070062]	0.8	0.49310.0072
**P63162;P14678**	**SNRPN;SNRPB**	Small nuclear ribonucleoprotein-associated protein N;Small nuclear ribonucleoprotein-associated proteins B and B	cyclooxygenase pathway [GO:0019371] drug metabolic process [GO:0017144] epithelial cell differentiation [GO:0030855]	cytosol [GO:0005829] extracellular exosome [GO:0070062] extracellular vesicle [GO:1903561]	0.8	0.49190.0067
**Q8NBQ5**	**HSD17B11**	Estradiol 17-beta-dehydrogenase 11	B cell differentiation [GO:0030183] B cell receptor signaling pathway [GO:0050853] Fc-epsilon receptor signaling pathway [GO:0038095]	cytosol [GO:0005829] extracellular exosome [GO:0070062] plasma membrane [GO:0005886]	0.8	0.49060.0075
**Q99729**	**HNRNPAB**	Heterogeneous nuclear ribonucleoprotein A/B	canonical glycolysis [GO:0061621] fructose 1,6-bisphosphate metabolic process [GO:0030388] fructose 6-phosphate metabolic process [GO:0006002]	6-phosphofructokinase complex [GO:0005945] cytosol [GO:0005829] extracellular exosome [GO:0070062]	0.8	0.49060.0075

**O00743**	**PPP6C**	Serine/threonine-protein phosphatase 6 catalytic subunit;Serine/threonine-protein phosphatase 6 catalytic subunit, N-terminally processed	formation of cytoplasmic translation initiation complex [GO:0001732] formation of translation preinitiation complex [GO:0001731] in utero embryonic development [GO:0001701]	cytoplasm [GO:0005737] cytosol [GO:0005829] eukaryotic translation initiation factor 2 complex [GO:0005850]	0.8	0.49010.0076
**P61247**	**RPS3A**	40S ribosomal protein S3a	animal organ morphogenesis [GO:0009887] cell adhesion [GO:0007155] cytokine-mediated signaling pathway [GO:0019221]	cell surface [GO:0009986] ficolin-1-rich granule membrane [GO:0101003] integrin complex [GO:0008305]	0.8	0.48970.0077
**P46459**	**NSF**	Vesicle-fusing ATPase	cellular response to DNA damage stimulus [GO:0006974] protein modification by small protein conjugation [GO:0032446] protein ubiquitination [GO:0016567]	cytoplasm [GO:0005737] cytosol [GO:0005829] extracellular exosome [GO:0070062]	1	0.48920.0077
**Q9GZN8**	**C20orf27**	UPF0687 protein C20orf27	aerobic respiration [GO:0009060] mitochondrial electron transport, ubiquinol to cytochrome c [GO:0006122] oxidative phosphorylation [GO:0006119]	mitochondrial inner membrane [GO:0005743] mitochondrial processing peptidase complex [GO:0017087] mitochondrial respiratory chain complex III [GO:0005750]	0.8	0.48870.0078
**Q15393**	**SF3B3**	Splicing factor 3B subunit 3	malate metabolic process [GO:0006108] pyruvate metabolic process [GO:0006090] regulation of NADP metabolic process [GO:1902031]	mitochondrial matrix [GO:0005759] mitochondrion [GO:0005739]	0.8	0.48770.0079
**Q6P4A8**	**PLBD1**	Phospholipase B-like 1;Phospholipase B-like 1 chain A;Phospholipase B-like 1 chain B;Phospholipase B-like 1 chain C	antimicrobial humoral response [GO:0019730] blood coagulation [GO:0007596] cell-cell junction maintenance [GO:0045217]	azurophil granule lumen [GO:0035578] cytosol [GO:0005829] extracellular exosome [GO:0070062]	0.8	0.48770.0079
**P53396**	**ACLY**	ATP-citrate synthase	G protein-coupled acetylcholine receptor signaling pathway [GO:0007213] G protein-coupled receptor signaling pathway [GO:0007186] cardiac muscle contraction [GO:0060048]	cilium [GO:0005929] cytoplasm [GO:0005737] cytosol [GO:0005829]	0.8	0.48720.0080

**P61088**	**UBE2N**	Ubiquitin-conjugating enzyme E2 N	RNA export from nucleus [GO:0006405] mRNA 3'-end processing [GO:0031124] mRNA export from nucleus [GO:0006406]	U2-type prespliceosome [GO:0071004] U2AF [GO:0089701] commitment complex [GO:0000243]	0.8	0.48520.0083
**Q96C86**	**DCPS**	m7GpppX diphosphatase	SRP-dependent cotranslational protein targeting to membrane [GO:0006614] embryonic brain development [GO:1990403] negative regulation of apoptotic process [GO:0043066]	cytosol [GO:0005829] cytosolic large ribosomal subunit [GO:0022625] endoplasmic reticulum [GO:0005783]	0.8	0.48280.0087
**Q9NR31**	**SAR1A**	GTP-binding protein SAR1a	purine-containing compound salvage [GO:0043101] pyrimidine nucleoside salvage [GO:0043097] pyrimidine nucleotide metabolic process [GO:0006220]	cytoplasm [GO:0005737] cytosol [GO:0005829] nucleus [GO:0005634]	0.8	0.48210.0081
**Q92688**	**ANP32B**	Acidic leucine-rich nuclear phosphoprotein 32 family member B	NADPH oxidation [GO:0070995] cell redox homeostasis [GO:0045454] cellular response to oxidative stress [GO:0034599]	cytoplasm [GO:0005737] cytoplasmic vesicle [GO:0031410] cytosol [GO:0005829]	0.8	0.47780.0094
**Q9NZK5**	**CECR1**	Adenosine deaminase CECR1	adenosine catabolic process [GO:0006154] signal transduction[GO:0007165] multicellular organism development[GO:0007275]	extracellular region[GO:0005576] extracellular space[GO:0005615] azurophil granule lumen[GO:0035578]	0.8	0.47590.0098
**P07203**	**GPX1**	Glutathione peroxidase 1	antigen processing and presentation of exogenous peptide antigen via MHC class I, TAP-dependent [GO:0002479] antigen processing and presentation of peptide antigen via MHC class I [GO:0002474] cell redox homeostasis [GO:0045454]	MHC class I peptide loading complex [GO:0042824] cell surface [GO:0009986] endoplasmic reticulum [GO:0005783]	1	0.47190.0105
**P61326;Q96A72**	**MAGOH;MAGOHB**	Protein mago nashi homolog;Protein mago nashi homolog 2	'de novo' IMP biosynthetic process [GO:0006189] animal organ regeneration [GO:0031100] brainstem development [GO:0003360]	cytosol [GO:0005829] extracellular exosome [GO:0070062] membrane [GO:0016020]	0.8	0.47110.0099
**P20702**	**ITGAX**	Integrin alpha-X	T cell proliferation [GO:0042098] activation of MAPK activity [GO:0000187] cell redox homeostasis [GO:0045454]	cytoplasm [GO:0005737] cytosol [GO:0005829] extracellular exosome [GO:0070062]	0.8	0.47090.0106

**P62318**	**SNRPD3**	Small nuclear ribonucleoprotein Sm D3	cell redox homeostasis [GO:0045454] nucleobase-containing small molecule interconversion [GO:0015949] positive regulation of membrane potential [GO:0045838]	cytosol [GO:0005829] extracellular exosome [GO:0070062] nucleus [GO:0005634]	0.8	0.46750.0113
**Q09028**	**RBBP4**	Histone-binding protein RBBP4	aging [GO:0007568] chromatin organization [GO:0006325] glutathione metabolic process [GO:0006749]	cytosol [GO:0005829] extracellular exosome [GO:0070062] mitochondrion [GO:0005739]	1	0.46750.0113
**P23368**	**ME2**	NAD-dependent malic enzyme, mitochondrial	T cell costimulation [GO:0031295] T cell receptor signaling pathway [GO:0050852] adaptive immune response [GO:0002250]	cell-cell junction [GO:0005911] cytoplasm [GO:0005737] cytosol [GO:0005829]	0.8	0.46650.0115
**Q14204**	**DYNC1H1**	Cytoplasmic dynein 1 heavy chain 1	COPII vesicle coating [GO:0048208] Golgi to plasma membrane protein transport [GO:0043001] Golgi vesicle docking [GO:0048211]	Golgi membrane [GO:0000139] Golgi stack [GO:0005795] cytosol [GO:0005829]	0.8	0.46650.0115
**P31939**	**ATIC**	Bifunctional purine biosynthesis protein PURH;Phosphoribosylaminoimidazolecarboxamide formyltransferase;IMP cyclohydrolase	2-oxoglutarate metabolic process [GO:0006103] NADP biosynthetic process [GO:0006741] NADP metabolic process [GO:0006739]	cytosol [GO:0005829] extracellular exosome [GO:0070062] mitochondrial matrix [GO:0005759]	0.8	0.46600.0116
**Q9Y262**	**EIF3L**	Eukaryotic translation initiation factor 3 subunit L	cellular response to estrogen stimulus [GO:0071391] immune response [GO:0006955] mast cell mediated immunity [GO:0002448]	Golgi apparatus [GO:0005794] collagen-containing extracellular matrix [GO:0062023] cytoplasm [GO:0005737]	0.8	0.45670.0136
**P36969**	**GPX4**	Phospholipid hydroperoxide glutathione peroxidase, mitochondrial	adult locomotory behavior [GO:0008344] associative learning [GO:0008306] brain development [GO:0007420]	Golgi apparatus [GO:0005794] axon [GO:0030424] cytosol [GO:0005829]	0.8	0.45600.0129
**P00491**	**PNP**	Purine nucleoside phosphorylase	Rab protein signal transduction [GO:0032482] antigen processing and presentation of exogenous peptide antigen via MHC class II [GO:0019886] autophagosome assembly [GO:0000045]	Golgi apparatus [GO:0005794] autophagosome membrane [GO:0000421] cytosol [GO:0005829]	0.8	0.45470.0140

**P11908**	**PRPS2**	Ribose-phosphate pyrophosphokinase 2	0	Sec61 translocon complex [GO:0005784] extracellular exosome [GO:0070062] integral component of membrane [GO:0016021]	0.8	0.45190.0139
**Q6P2Q9**	**PRPF8**	Pre-mRNA-processing-splicing factor 8	IRE1-mediated unfolded protein response [GO:0036498] cellular response to interleukin-7 [GO:0098761] negative regulation of transcription by RNA polymerase II [GO:0000122]	collagen-containing extracellular matrix [GO:0062023] cytoplasm [GO:0005737] extracellular region [GO:0005576]	0.8	0.45070.0149
**O00410**	**IPO5**	Importin-5	Rho protein signal transduction [GO:0007266] negative regulation of apoptotic process [GO:0043066] negative regulation of axonogenesis [GO:0050771]	cytoskeleton [GO:0005856] cytosol [GO:0005829] extracellular exosome [GO:0070062]	0.8	0.45020.0151
**P62195**	**PSMC5**	26S protease regulatory subunit 8	heme catabolic process [GO:0042167] oxidation-reduction process [GO:0055114]	cytosol [GO:0005829] extracellular exosome [GO:0070062]	0.8	0.44830.0155
**P30046;A6NHG4**	**DDT;DDTL**	D-dopachrome decarboxylase;D-dopachrome decarboxylase-like protein	acetyl-CoA biosynthetic process [GO:0006085] cholesterol biosynthetic process [GO:0006695] citrate metabolic process [GO:0006101]	azurophil granule lumen [GO:0035578] citrate lyase complex [GO:0009346] cytosol [GO:0005829]	0.8	0.44780.0148
**P06865**	**HEXA**	Beta-hexosaminidase subunit alpha	arginyl-tRNA aminoacylation [GO:0006420] tRNA aminoacylation for protein translation [GO:0006418]	aminoacyl-tRNA synthetase multienzyme complex [GO:0017101] cytoplasm [GO:0005737] cytosol [GO:0005829]	0.8	0.44630.0160
**Q16718**	**NDUFA5**	NADH dehydrogenase [ubiquinone] 1 alpha subcomplex subunit 5	COPII vesicle coating [GO:0048208] SNARE complex disassembly [GO:0035494] apical protein localization [GO:0045176]	Golgi membrane [GO:0000139] SNARE complex [GO:0031201] cytosol [GO:0005829]	0.8	0.44600.0153
**O75874**	**IDH1**	Isocitrate dehydrogenase [NADP] cytoplasmic	cardiolipin acyl-chain remodeling [GO:0035965] fatty acid beta-oxidation [GO:0006635]	endoplasmic reticulum [GO:0005783] mitochondrial envelope [GO:0005740] mitochondrial inner membrane [GO:0005743]	1	0.44290.0170

**P00367;P49448**	**GLUD1;GLUD2**	Glutamate dehydrogenase 1, mitochondrial;Glutamate dehydrogenase 2, mitochondrial	dopaminergic neuron differentiation [GO:0071542] neuron projection development [GO:0031175] platelet degranulation [GO:0002576]	cytosol [GO:0005829] endoplasmic reticulum [GO:0005783] endoplasmic reticulum lumen [GO:0005788]	1	0.44190.0172
**P01903**	**HLA-DRA**	HLA class II histocompatibility antigen, DR alpha chain	formation of cytoplasmic translation initiation complex [GO:0001732] negative regulation of translational initiation [GO:0045947] nuclear-transcribed mRNA catabolic process, nonsense-mediated decay [GO:0000184]	PML body [GO:0016605] cytoplasm [GO:0005737] cytosol [GO:0005829]	1	0.44190.0172
**P52565**	**ARHGDIA**	Rho GDP-dissociation inhibitor 1	DNA double-strand break processing [GO:0000729] JNK cascade [GO:0007254] T cell receptor signaling pathway [GO:0050852]	UBC13-MMS2 complex [GO:0031372] UBC13-UEV1A complex [GO:0035370] cytoplasm [GO:0005737]	1	0.44090.0175
**P41240**	**CSK**	Tyrosine-protein kinase CSK	SRP-dependent cotranslational protein targeting to membrane [GO:0006614] cell differentiation [GO:0030154] negative regulation of apoptotic process [GO:0043066]	cytoplasm [GO:0005737] cytosol [GO:0005829] cytosolic small ribosomal subunit [GO:0022627]	0.8	0.44040.0176
**P50453**	**SERPINB9**	Serpin B9	mast cell mediated immunity[GO:0002448] apoptotic process[GO:0006915] immune response [GO:0006955]	extracellular space [GO:0005615]nucleus[GO:0005634] nucleoplasm[GO:0005654]	0.8	0.43740.0185
**P08631**	**HCK**	Tyrosine-protein kinase HCK	RNA metabolic process [GO:0016070] RNA processing [GO:0006396] mRNA splicing, via spliceosome [GO:0000398]	catalytic step 2 spliceosome [GO:0071013] cell projection [GO:0042995] cytoplasm [GO:0005737]	0.8	0.43690.0186
**Q92835**	**INPP5D**	Phosphatidylinositol 3,4,5-trisphosphate 5-phosphatase 1	Fc-epsilon receptor signaling pathway [GO:0038095] MAPK cascade [GO:0000165] NIK/NF-kappaB signaling [GO:0038061]	blood microparticle [GO:0072562] cytoplasm [GO:0005737] cytoplasmic vesicle [GO:0031410]	0.8	0.43650.0188

**Q14498**	**RBM39**	RNA-binding protein 39	SRP-dependent cotranslational protein targeting to membrane [GO:0006614] maturation of SSU-rRNA from tricistronic rRNA transcript (SSU-rRNA, 5.8S rRNA, LSU-rRNA) [GO:0000462] nuclear-transcribed mRNA catabolic process, nonsense-mediated decay [GO:0000184]	cytosol [GO:0005829] cytosolic small ribosomal subunit [GO:0022627] endoplasmic reticulum [GO:0005783]	0.8	0.43450.0194
**Q9Y376**	**CAB39**	Calcium-binding protein 39	RNA splicing [GO:0008380] histone mRNA metabolic process [GO:0008334] import into nucleus [GO:0051170]	SMN-Sm protein complex [GO:0034719] U1 snRNP [GO:0005685] U12-type spliceosomal complex [GO:0005689]	0.8	0.43450.0194
**P06737**	**PYGL**	Glycogen phosphorylase, liver form	actin filament organization [GO:0007015] cytoplasmic sequestering of NF-kappaB [GO:0007253] negative regulation of NF-kappaB transcription factor activity [GO:0032088]	cytoplasm [GO:0005737] cytoskeleton [GO:0005856] cytosol [GO:0005829]	0.8	0.43350.0197
**Q8N163**	**CCAR2**	Cell cycle and apoptosis regulator protein 2	Wnt signaling pathway, planar cell polarity pathway [GO:0060071] antigen processing and presentation of exogenous peptide antigen via MHC class II [GO:0019886] aorta development [GO:0035904]	AP-2 adaptor complex [GO:0030122] clathrin-coated endocytic vesicle [GO:0045334] clathrin-coated endocytic vesicle membrane [GO:0030669]	0.8	0.43250.0200
**Q14683**	**SMC1A**	Structural maintenance of chromosomes protein 1A	mitotic sister chromatid segregation[GO:0000070] DNA repair[GO:0006281] cellular response to DNA damage stimulus [GO:0006974]	chromosome, centromeric region[GO:0000775] kinetochore[GO:0000776] condensed chromosome kinetochore [GO:0000777]	0.8	0.43100.0204
**P24158**	**PRTN3**	Myeloblastin	Fc-epsilon receptor signaling pathway [GO:0038095] G2/M transition of mitotic cell cycle [GO:0000086] NIK/NF-kappaB signaling [GO:0038061]	Cul7-RING ubiquitin ligase complex [GO:0031467] SCF ubiquitin ligase complex [GO:0019005] centrosome [GO:0005813]	1	0.43000.0207
**Q9NR45**	**NANS**	Sialic acid synthase	CRD-mediated mRNA stabilization [GO:0070934] RNA localization to chromatin [GO:1990280] RNA metabolic process [GO:0016070]	CRD-mediated mRNA stability complex [GO:0070937] catalytic step 2 spliceosome [GO:0071013] cell surface [GO:0009986]	0.8	0.43000.0207

**O95466**	**FMNL1**	Formin-like protein 1	substrate-dependent cell migration[GO:0006929] cytoskeleton organization[GO:0007010] regulation of cell shape[GO:0008360]	lamin filament [GO:0005638] nuclear inner membrane [GO:0005637] nuclear membrane [GO:0031965]	0.8	0.42910.0211
**P07948**	**LYN**	Tyrosine-protein kinase Lyn	CRD-mediated mRNA stabilization [GO:0070934] DNA duplex unwinding [GO:0032508] DNA replication [GO:0006260]	CRD-mediated mRNA stability complex [GO:0070937] RISC complex [GO:0016442] RISC-loading complex [GO:0070578]	0.8	0.42910.0211
**Q9NUV9**	**GIMAP4**	GTPase IMAP family member 4	DNA repair [GO:0006281] DNA replication [GO:0006260] regulation of signal transduction by p53 class mediator [GO:1901796]	FACT complex [GO:0035101] nuclear chromatin [GO:0000790] nucleolus [GO:0005730]	0.8	0.42910.0211
**P16152**	**CBR1**	Carbonyl reductase [NADPH] 1	ATP-dependent chromatin remodeling [GO:0043044] CENP-A containing nucleosome assembly [GO:0034080] DNA replication [GO:0006260]	CAF-1 complex [GO:0033186] ESC/E(Z) complex [GO:0035098] NURF complex [GO:0016589]	0.8	0.42760.0215
**P27707**	**DCK**	Deoxycytidine kinase	angiogenesis [GO:0001525] apoptotic process [GO:0006915] cell adhesion [GO:0007155]	Golgi apparatus [GO:0005794] aminoacyl-tRNA synthetase multienzyme complex [GO:0017101] cell surface [GO:0009986]	0.8	0.42710.0217
**O00182;Q6DKI2;Q3B8N2**	**LGALS9;LGALS9C;LGALS9B**	Galectin-9;Galectin-9C;Galectin-9B	neutrophil degranulation [GO:0043312] positive regulation of transcription, DNA-templated [GO:0045893] transcription, DNA-templated [GO:0006351]	extracellular region [GO:0005576] ficolin-1-rich granule lumen [GO:1904813] membrane [GO:0016020]	0.8	0.42570.0213
**P04843**	**RPN1**	Dolichyl-diphosphooligosaccharide--protein glycosyltransferase subunit 1	actin cytoskeleton organization [GO:0030036] actin filament severing [GO:0051014] multicellular organism development [GO:0007275]	brush border [GO:0005903] cytosol [GO:0005829] focal adhesion [GO:0005925]	0.8	0.42560.0222

**Q9NUQ9**	**FAM49B**	Protein FAM49B	RNA export from nucleus [GO:0006405] RNA secondary structure unwinding [GO:0010501] RNA splicing [GO:0008380]	cytoplasm [GO:0005737] nuclear matrix [GO:0016363] nuclear speck [GO:0016607]	1	0.42170.0236
**P05091**	**ALDH2**	Aldehyde dehydrogenase, mitochondrial	IRES-dependent viral translational initiation [GO:0075522] formation of cytoplasmic translation initiation complex [GO:0001732] negative regulation of ERK1 and ERK2 cascade [GO:0070373]	cytoplasm [GO:0005737] cytosol [GO:0005829] eukaryotic 43S preinitiation complex [GO:0016282]	0.8	0.41970.0243
**O60506**	**SYNCRIP**	Heterogeneous nuclear ribonucleoprotein Q	G2/M transition of mitotic cell cycle [GO:0000086] antigen processing and presentation of exogenous peptide antigen via MHC class II [GO:0019886] cell division [GO:0051301]	axon cytoplasm [GO:1904115] azurophil granule lumen [GO:0035578] cell cortex [GO:0005938]	0.8	0.41870.0246
**P20042**	**EIF2S2**	Eukaryotic translation initiation factor 2 subunit 2	RNA processing [GO:0006396] RNA splicing [GO:0008380] mRNA processing [GO:0006397]	microtubule cytoskeleton [GO:0015630] microtubule organizing center [GO:0005815] nuclear speck [GO:0016607]	0.8	0.41770.0250
**P16885**	**PLCG2**	1-phosphatidylinositol 4,5-bisphosphate phosphodiesterase gamma-2	DNA repair [GO:0006281] cell division [GO:0051301] meiotic cell cycle [GO:0051321]	chromosome [GO:0005694] chromosome, centromeric region [GO:0000775] cohesin complex [GO:0008278]	0.8	0.41430.0263
**Q00839**	**HNRNPU**	Heterogeneous nuclear ribonucleoprotein U	ERK1 and ERK2 cascade [GO:0070371] activation of MAPK activity [GO:0000187] basophil activation involved in immune response [GO:0002276]	aminoacyl-tRNA synthetase multienzyme complex [GO:0017101] cytosol [GO:0005829] extracellular space [GO:0005615]	1	0.41280.0269
**Q99829**	**CPNE1**	Copine-1	RNA splicing, via transesterification reactions [GO:0000375] mRNA splicing, via spliceosome [GO:0000398] negative regulation of protein catabolic process [GO:0042177]	U12-type spliceosomal complex [GO:0005689] U2-type precatalytic spliceosome [GO:0071005] catalytic step 2 spliceosome [GO:0071013]	0.8	0.40970.0273

**O75436**	**VPS26A**	Vacuolar protein sorting-associated protein 26A	DNA recombination [GO:0006310] production of siRNA involved in RNA interference [GO:0030422]	cytoplasm [GO:0005737] cytosol [GO:0005829] nucleus [GO:0005634]	0.8	0.40450.0295
**P55084**	**HADHB**	Trifunctional enzyme subunit beta, mitochondrial;3-ketoacyl-CoA thiolase	mitochondrial electron transport, NADH to ubiquinone [GO:0006120] mitochondrial respiratory chain complex I assembly [GO:0032981]	mitochondrial inner membrane [GO:0005743] mitochondrial respiratory chain complex I [GO:0005747]	1	0.40390.0306
**P14598**	**NCF1**	Neutrophil cytosol factor 1	BMP signaling pathway [GO:0030509] apoptotic process [GO:0006915] cell aging [GO:0007569]	cytoplasm [GO:0005737] cytosol [GO:0005829] nucleus [GO:0005634]	1	0.40340.0309
**P22314**	**UBA1**	Ubiquitin-like modifier-activating enzyme 1	cellular hyperosmotic response [GO:0071474] skeletal muscle contraction [GO:0003009]	actin filament [GO:0005884]	1	0.40150.0317
**P63010**	**AP2B1**	AP-2 complex subunit beta	RNA splicing [GO:0008380] RNA splicing, via transesterification reactions [GO:0000375] cellular response to lipopolysaccharide [GO:0071222]	U2-type catalytic step 1 spliceosome [GO:0071006] U2-type catalytic step 2 spliceosome [GO:0071007] U2-type precatalytic spliceosome [GO:0071005]	0.8	0.40100.0320
**P51149**	**RAB7A**	Ras-related protein Rab-7a	lipid catabolic process [GO:0016042] phosphatidylcholine acyl-chain remodeling [GO:0036151] phosphatidylethanolamine acyl-chain remodeling [GO:0036152]	cytosol [GO:0005829] extracellular space [GO:0005615] lysosome [GO:0005764]	1	0.40050.0322
**P05120**	**SERPINB2**	Plasminogen activator inhibitor 2	Golgi to endosome transport [GO:0006895] mitochondrion organization [GO:0007005] negative regulation of parkin-mediated stimulation of mitophagy in response to mitochondrial depolarization [GO:1905090]	cytoplasm [GO:0005737] cytosol [GO:0005829] extracellular exosome [GO:0070062]	0.8	0.40000.0324
**P0DMV9;P0DMV8**	**HSPA1B;HSPA1A**	Heat shock 70 kDa protein 1B;Heat shock 70 kDa0 protein 1A		cytoplasm [GO:0005737] membrane [GO:0016020]	1	0.39950.0327
**P07355;A6NMY6**	**ANXA2;ANXA2P2**	Annexin A2;Putative annexin A2-like protein	RNA splicing [GO:0008380] Wnt signaling pathway [GO:0016055] cell cycle [GO:0007049]	DBIRD complex [GO:0044609] cytoplasm [GO:0005737] mitochondrial matrix [GO:0005759]	1	0.39800.0333

**Q9BSJ8**	**ESYT1**	Extended synaptotagmin-1	androgen catabolic process [GO:0006710] estrogen biosynthetic process [GO:0006703]	cytoplasm [GO:0005737] cytosol [GO:0005829] endoplasmic reticulum [GO:0005783]	0.8	0.39410.0353
**O43390**	**HNRNPR**	Heterogeneous nuclear ribonucleoprotein R	COPII vesicle coating [GO:0048208] cell morphogenesis [GO:0000902] negative regulation of autophagosome assembly [GO:1902902]	Golgi cisterna membrane [GO:0032580] Golgi-associated vesicle [GO:0005798] cis-Golgi network [GO:0005801]	0.8	0.39360.0355
**Q99613;B5ME19**	**EIF3C;EIF3CL**	Eukaryotic translation initiation factor 3 subunit C;Eukaryotic translation initiation factor 3 subunit C-like protein	actin cytoskeleton organization [GO:0030036] alpha-beta T cell proliferation [GO:0046633] chemotaxis [GO:0006935]	cytoskeleton [GO:0005856] cytosol [GO:0005829] extracellular exosome [GO:0070062]	0.8	0.39260.0360
**Q12904**	**AIMP1**	Aminoacyl tRNA synthase complex-interacting multifunctional protein 1;Endothelial monocyte-activating polypeptide 2	activation of cysteine-type endopeptidase activity involved in apoptotic process [GO:0006919] inner ear development [GO:0048839] negative regulation of cell differentiation [GO:0045596]	cytoplasm [GO:0005737] extracellular exosome [GO:0070062] nucleus [GO:0005634]	0.8	0.39040.0363
**Q13838**	**DDX39B**	Spliceosome RNA helicase DDX39B	T cell receptor signaling pathway [GO:0050852] apoptotic process [GO:0006915] cytokine-mediated signaling pathway [GO:0019221]	cytoskeleton [GO:0005856] cytosol [GO:0005829] membrane raft [GO:0045121]	1	0.39010.0373
**P51571**	**SSR4**	Translocon-associated protein subunit delta	RNA processing [GO:0006396] alternative mRNA splicing, via spliceosome [GO:0000380] androgen receptor signaling pathway [GO:0030521]	cytoplasm [GO:0005737] cytosol [GO:0005829] membrane [GO:0016020]	0.8	0.38690.0381
**Q9UKK9**	**NUDT5**	ADP-sugar pyrophosphatase	G protein-coupled receptor signaling pathway [GO:0007186] actin filament organization [GO:0007015] asymmetric neuroblast division [GO:0055059]	Golgi apparatus [GO:0005794] bicellular tight junction [GO:0005923] cytoplasm [GO:0005737]	0.8	0.38520.0399
**P62241**	**RPS8**	40S ribosomal protein S8	nuclear-transcribed mRNA catabolic process, nonsense-mediated decay[GO:0000184] maturation of SSU-rRNA from tricistronic rRNA transcript (SSU-rRNA, 5.8S rRNA, LSU-rRNA)[GO:0000462] translation[GO:0006412]	cytoplasm [GO:0005737] endoplasmic reticulum [GO:0005783] endoplasmic reticulum membrane [GO:0005789]	0.8	0.38350.0400

**Q709C8**	**VPS13C**	Vacuolar protein sorting-associated protein 13C	cellular response to menadione [GO:0036245] deadenylation-dependent decapping of nuclear-transcribed mRNA [GO:0000290] exonucleolytic catabolism of deadenylated mRNA [GO:0043928]	P-body [GO:0000932] cytoplasm [GO:0005737] cytosol [GO:0005829]	0.8	0.38250.0406
**Q15046**	**KARS**	Lysine--tRNA ligase	DNA recombination [GO:0006310] DNA repair [GO:0006281] DNA replication [GO:0006260]	cytoplasm [GO:0005737] mediator complex [GO:0016592] nuclear speck [GO:0016607]	0.8	0.37730.0444
**Q9BZZ5**	**API5**	Apoptosis inhibitor 5	T cell receptor signaling pathway [GO:0050852] integrin-mediated signaling pathway [GO:0007229] protein localization to plasma membrane [GO:0072659]	plasma membrane [GO:0005886] protein-containing complex [GO:0032991]	0.8	0.37560.0447
**O43399**	**TPD52L2**	Tumor protein D54	-	-	0.8	0.37490.0459
**Q03252**	**LMNB2**	Lamin-B2	epithelial to mesenchymal transition [GO:0001837] positive regulation of transcription, DNA-templated [GO:0045893]	RNA polymerase II transcription factor complex [GO:0090575] cytoplasm [GO:0005737] nucleoplasm [GO:0005654]	0.8	0.37490.0459
**Q96AG4**	**LRRC59**	Leucine-rich repeat-containing protein 59	cellular response to calcium ion [GO:0071277] glycerophospholipid biosynthetic process [GO:0046474] lipid metabolic process [GO:0006629]	azurophil granule membrane [GO:0035577] cytoplasm [GO:0005737] cytosol [GO:0005829]	0.8	0.37440.0462
**P05387**	**RPLP2**	60S acidic ribosomal protein P2	endoplasmic reticulum-plasma membrane tethering [GO:0061817] glycosphingolipid metabolic process [GO:0006687] lipid transport [GO:0006869]	endoplasmic reticulum [GO:0005783] endoplasmic reticulum membrane [GO:0005789] extrinsic component of cytoplasmic side of plasma membrane [GO:0031234]	0.8	0.37390.0465
**P00338**	**LDHA**	L-lactate dehydrogenase A chain	apoptotic process [GO:0006915] negative regulation of apoptotic process [GO:0043066] negative regulation of fibroblast apoptotic process [GO:2000270]	cytoplasm [GO:0005737] membrane [GO:0016020] nuclear speck [GO:0016607]	1	0.37340.0468

**P55145**	**MANF**	Mesencephalic astrocyte-derived neurotrophic factor	platelet degranulation[GO:0002576] response to unfolded protein[GO:0006986] signal transduction[GO:0007165]	extracellular region[GO:0005576] extracellular space[GO:0005615] nucleus[GO:0005634]	0.8	-0.3759	0.0453
**P61978**	**HNRNPK**	Heterogeneous nuclear ribonucleoprotein K	mRNA splicing, via spliceosome[GO:0000398] regulation of transcription by RNA polymerase II [GO:0006357] RNA processing [GO:0006396]	extracellular exosome [GO:0070062] mitochondrion [GO:0005739]	1	-0.3808	0.0424
**Q96PK6**	**RBM14**	RNA-binding protein 14	asparagine metabolic process [GO:0006528] glutamine metabolic process [GO:0006541] neutrophil degranulation [GO:0043312]	centrosome [GO:0005813] cytosol [GO:0005829] extracellular exosome [GO:0070062]	0.8	-0.3847	0.0402
**Q96QH2**	**PRAM1**	PML-RARA-regulated adapter molecule 1	COPII-coated vesicle cargo loading [GO:0090110] endoplasmic reticulum to Golgi vesicle-mediated transport [GO:0006888] intracellular protein transport [GO:0006886]	COPII vesicle coat [GO:0030127] Golgi membrane [GO:0000139] endoplasmic reticulum [GO:0005783]	1	-0.4054	0.0300
**P51858**	**HDGF**	Hepatoma-derived growth factor	CMP-N-acetylneuraminate biosynthetic process [GO:0006055] carbohydrate biosynthetic process [GO:0016051]	cytoplasm [GO:0005737] cytosol [GO:0005829] extracellular exosome [GO:0070062]	1	-0.4158	0.0258
**O00161**	**SNAP23**	Synaptosomal-associated protein 23	platelet degranulation [GO:0002576] positive regulation of T cell activation [GO:0050870] positive regulation of T cell mediated cytotoxicity [GO:0001916]	cilium [GO:0005929] extracellular exosome [GO:0070062] extracellular region [GO:0005576]	0.8	-0.4276	0.0215
**Q9UBR2**	**CTSZ**	Cathepsin Z	COPII vesicle coating [GO:0048208] angiotensin maturation [GO:0002003] endoplasmic reticulum to Golgi vesicle-mediated transport [GO:0006888]	cytosol [GO:0005829]	1	-0.4291	0.0211
**P06730**	**EIF4E**	Eukaryotic translation initiation factor 4E	adenosine catabolic process [GO:0006154] cellular protein metabolic process [GO:0044267] inosine biosynthetic process [GO:0046103]	azurophil granule lumen [GO:0035578] extracellular region [GO:0005576] extracellular space [GO:0005615]	0.8	-0.4380	0.0175

**P30101**	**PDIA3**	Protein disulfide-isomerase A3	COPII vesicle coating [GO:0048208] angiotensin maturation [GO:0002003] endoplasmic reticulum to Golgi vesicle-mediated transport [GO:0006888]	COPII-coated ER to Golgi transport vesicle [GO:0030134] Golgi membrane [GO:0000139] cell cortex region [GO:0099738]	1	-0.4483	0.0155
**Q6JBY9**	**RCSD1**	CapZ-interacting protein	skeletal muscle contraction[GO:0003009] cellular hyperosmotic response[GO:0071474]	cytosol [GO:0005829] mitochondrion [GO:0005739] nucleus [GO:0005634]	0.8	-0.4542	0.0141
**P14317**	**HCLS1**	Hematopoietic lineage cell-specific protein	ATP generation from poly-ADP-D-ribose [GO:1990966] D-ribose catabolic process [GO:0019303] chromatin remodeling [GO:0006338]	cytosol [GO:0005829] extracellular exosome [GO:0070062] nucleus [GO:0005634]	1	-0.5079	0.0055
**P62328**	**TMSB4X**	Thymosin beta-4;Hematopoietic system regulatory peptide	formation of cytoplasmic translation initiation complex [GO:0001732] translational initiation [GO:0006413] viral translational termination-reinitiation [GO:0075525]	cytosol [GO:0005829] eukaryotic 43S preinitiation complex [GO:0016282] eukaryotic 48S preinitiation complex [GO:0033290]	1	-0.5108	0.0052
**P32119**	**PRDX2**	Peroxiredoxin-2	cell cycle arrest [GO:0007050] cellular hypotonic response [GO:0071476] intracellular signal transduction [GO:0035556]	cytosol [GO:0005829] extracellular exosome [GO:0070062] extracellular region [GO:0005576]	0.8	-0.5481	0.0021

Table S7: DEP$_A$ as referenced in MitoCarta3.0. Shown are all MitoCarta3.0 entries for DEP$_A$ identified at the baseline (T0), post-stress (T1) or at both measures (T0T1). MIM = mitochondrial inner membrane, MOM= mitochondrial outer membrane.

Protein (Gene symbol))	Time	SubMito Localization	MitoPathways
**COX5A**	T0	MIM	OXPHOS > Complex IV > CIV subunits | OXPHOS > OXPHOS subunits
**LRPPRC**	T0	Matrix	Mitochondrial central dogma > mtRNA metabolism > mtRNA stability and decay | Mitochondrial central dogma > Translation
**VDAC1**	T0	MOM	Signaling > Calcium homeostasis > Mitochondrial permeability transition pore | Mitochondrial dynamics and surveillance > Organelle contact sites | Small molecule transport
**MTCH2**	T0	MOM	Small molecule transport > SLC25A family | Mitochondrial dynamics and surveillance > Fusion
**ACSL1**	T0	MOM	Metabolism > Lipid metabolism > Fatty acid oxidation
**HADH**	T0	Matrix	Metabolism > Lipid metabolism > Fatty acid oxidation | Metabolism > Amino acid metabolism > Lysine metabolism
**NDUFA9**	T1	MIM	OXPHOS > Complex I > CI subunits | OXPHOS > OXPHOS subunits
**BID**	T1	MOM	Mitochondrial dynamics and surveillance > Apoptosis
**SLC25A6**	T1	MIM	Metabolism > Nucleotide metabolism > Nucleotide import | Signaling > Calcium homeostasis > Mitochondrial permeability transition pore | Small molecule transport > SLC25A family
**ACAA1**	T1	unknown	Metabolism > Lipid metabolism
**TXNRD1**	T1	unknown	Metabolism > Detoxification > ROS and glutathione metabolism | Metabolism > Detoxification > Selenoproteins
**NNT**	T1	MIM	Metabolism > Metals and cofactors > NAD biosynthesis and metabolism
**ATP5J2**	T1	MIM	OXPHOS > Complex V > CV subunits | OXPHOS > OXPHOS subunits
**PHB2**	T1	MIM	Protein import, sorting and homeostasis > Protein homeostasis > Chaperones
**MT-CO2**	T0T1	MIM	OXPHOS > Complex IV > CIV subunits | OXPHOS > OXPHOS subunits
**COX6C**	T0T1	MIM	OXPHOS > Complex IV > CIV subunits | OXPHOS > OXPHOS subunits
**VDAC2**	T0T1	MOM	Small molecule transport
**SQRDL**	T0T1	MIM	Metabolism > Electron carriers > Q-linked reactions, other | Metabolism > Sulfur metabolism
**MGST3**	T0T1	MOM	Metabolism > Lipid metabolism > Eicosanoid metabolism
**NDUFS1**	T0T1	MIM	OXPHOS > Complex I > CI subunits | Metabolism > Metals and cofactors > Fe-S-containing proteins | OXPHOS > OXPHOS subunits
**COMT**	T0T1	Membrane	Metabolism > Amino acid metabolism > Catechol metabolism
**ATP5H**	T0T1	MIM	OXPHOS > Complex V > CV subunits | OXPHOS > OXPHOS subunits
**SLC25A3**	T0T1	MIM	Metabolism > Metals and cofactors > Copper metabolism | Signaling > Calcium homeostasis > Calcium cycle | Small molecule transport > SLC25A family
**CYC1**	T0T1	MIM	OXPHOS > Complex III > CIII subunits | Metabolism > Metals and cofactors > Heme-containing proteins | Metabolism > Electron carriers > Cytochromes | OXPHOS > OXPHOS subunits
**ATP5F1B**	T0T1	MIM	OXPHOS > Complex V > CV subunits | OXPHOS > OXPHOS subunits
**GPD2**	T0T1	MIM	Metabolism > Carbohydrate metabolism > Glycerol phosphate shuttle | Metabolism > Electron carriers > Q-linked reactions, other

Table S8: HUB proteins as referenced in MitoCarta3.0. Shown are all MitoCarta3.0 entries for HUB proteins from baseline co-expression modules. MIM = mitochondrial inner membrane, MOM= mitochondrial outer membrane.

Hubprotein (gene symbol)	Module	SubMitoLocalization	MitoPathways
**UQCRH**	Salmon	MIM	OXPHOS > Complex III > CIII subunits | OXPHOS > OXPHOS subunits
**SLC25A5**	Purple	MIM	Metabolism > Nucleotide metabolism > Nucleotide import | Signaling > Calcium homeostasis > Mitochondrial permeability transition pore | Small molecule transport > SLC25A family
**CYC1**	Purple	MIM	OXPHOS > Complex III > CIII subunits | Metabolism > Metals and cofactors > Heme-containing proteins | Metabolism > Electron carriers > Cytochromes | OXPHOS > OXPHOS subunits
**VDAC1**	Purple	MOM	Signaling > Calcium homeostasis > Mitochondrial permeability transition pore | Mitochondrial dynamics and surveillance > Organelle contact sites | Small molecule transport
**ATP5F1**	Purple	MIM	OXPHOS > Complex V > CV subunits | OXPHOS > OXPHOS subunits
**VDAC2**	Purple	MOM	Small molecule transport

S12 References

1. Bernstein DP, Stein JA, Newcomb MD, Walker E, Pogge D, Ahluvalia T*, et al.* (2003): Development and validation of a brief screening version of the Childhood Trauma Questionnaire. *Child abuse & neglect* 27: 169–190.
2. Wingenfeld K, Spitzer C, Mensebach C, Grabe HJ, Hill A, Gast U*, et al.* (2010): Die deutsche Version des Childhood Trauma Questionnaire (CTQ): Erste Befunde zu den psychometrischen Kennwerten. *Psychotherapie, Psychosomatik, medizinische Psychologie* 60: e13.
3. Bremner JD, Vermetten E, Mazure CM (2000): Development and preliminary psychometric properties of an instrument for the measurement of childhood trauma: The early trauma inventory. *Depress. Anxiety* 12: 1–12.
4. Wingenfeld K, Driessen M, Mensebach C, Rullkoetter N, Schaffrath C, Spitzer C*, et al.* (2011): Die deutsche Version des „Early Trauma Inventory" (ETI). *Diagnostica* 57: 27–38.
5. Wittchen HU, Wunderlich U, Gruschwitz S, Zaudig M (1996): Strukturiertes Klinisches Interview für DSM-IV (SKID). Göttingen: Belz.
6. Schumacher J, Leppert K, Gunzelrnann T, Strauß B, Brähler E (2005): Die Resilienzskala - Ein Fragebogen zur Erfassung der psychischen Widerstandsfähigkeit als Personmerkmal. [The Resilience Scale - A questionnaire to assess resilience as a personality characteristic.]. *Zeitschrift für Klinische Psychologie, Psychiatrie und Psychotherapie* 53: 16–39.
7. Franke G (2000): *Brief Symptom Inventory (BSI).* Göttingen: Beltz.
8. Kirschbaum C, Pirke KM, Hellhammer DH (1993): The 'Trier Social Stress Test'--a tool for investigating psychobiological stress responses in a laboratory setting. *Neuropsychobiology* 28: 76–81.
9. Kirschbaum C, Wüst S, Hellhammer D (1992): Consistent sex differences in cortisol responses to psychological stress. *Psychosomatic medicine* 54: 648–657.
10. Dickerson SS, Kemeny ME (2004): Acute stressors and cortisol responses: a theoretical integration and synthesis of laboratory research. *Psychological bulletin* 130: 355–391.
11. Kudielka BM, Hellhammer DH, Kirschbaum C (2007): Ten Years of Research with the Trier Social Stress Test--Revisited. In: Harmon-Jones E, Winkielman P. *Social neuroscience: Integrating biological and psychological explanations of social behavior*. New York: The Guilford Press, pp 56–83.
12. Plum S, Steinbach S, Abel L, Marcus K, Helling S, May C (2015): Proteomics in neurodegenerative diseases: Methods for obtaining a closer look at the neuronal proteome. *Proteomics. Clinical applications* 9: 848–871.
13. Kil YJ, Becker C, Sandoval W, Goldberg D, Bern M (2011): Preview: a program for surveying shotgun proteomics tandem mass spectrometry data. *Analytical chemistry* 83: 5259–5267.
14. Cox J, Neuhauser N, Michalski A, Scheltema RA, Olsen JV, Mann M (2011): Andromeda: a peptide search engine integrated into the MaxQuant environment. *Journal of proteome research* 10: 1794–1805.
15. Cox J, Hein MY, Luber CA, Paron I, Nagaraj N, Mann M (2014): Accurate proteome-wide label-free quantification by delayed normalization and maximal peptide ratio extraction, termed MaxLFQ. *Molecular & cellular proteomics : MCP* 13: 2513–2526.
16. Karpievitch YV, Dabney AR, Smith RD (2012): Normalization and missing value imputation for label-free LC-MS analysis. *BMC bioinformatics* 13 Suppl 16: S5.
17. Lazar C, Gatto L, Ferro M, Bruley C, Burger T (2016): Accounting for the Multiple Natures of Missing Values in Label-Free Quantitative Proteomics Data Sets to Compare Imputation Strategies. *Journal of proteome research* 15: 1116–1125.
18. Wang JZ, Du Z, Payattakool R, Yu PS, Chen C-F (2007): A new method to measure the semantic similarity of GO terms. *Bioinformatics (Oxford, England)* 23: 1274–1281.
19. Charrad M, Ghazzali N, Boiteau V, Niknafs A (2014): NbClust : An R Package for Determining the Relevant Number of Clusters in a Data Set. *J. Stat. Soft.* 61.
